# Changes in Fungal Community Composition in Response to Elevated Atmospheric CO_2_ and Nitrogen Fertilization Varies with Soil Horizon

**DOI:** 10.3389/fmicb.2013.00078

**Published:** 2013-04-09

**Authors:** Carolyn F. Weber, Rytas Vilgalys, Cheryl R. Kuske

**Affiliations:** ^1^Bioscience Division, Los Alamos National LaboratoryLos Alamos, NM, USA; ^2^Department of Biology, Duke UniversityDurham, NC, USA

**Keywords:** soil fungi, elevated CO_2_, nitrogen fertilization, forest floor, soil chemistry, Ascomycota, basidiomycota, agaricales

## Abstract

Increasing levels of atmospheric carbon dioxide (CO_2_) and rates of nitrogen (N)-deposition to forest ecosystems are predicted to alter the structure and function of soil fungal communities, but the spatially heterogeneous distribution of soil fungi has hampered investigations aimed at understanding such impacts. We hypothesized that soil physical and chemical properties and fungal community composition would be differentially impacted by elevated atmospheric CO_2_ (eCO_2_) and N-fertilization in spatially separated field samples, in the forest floor, 0–2, 2–5, and 5–10 cm depth intervals in a loblolly pine Free-Air Carbon Dioxide Enrichment (FACE) experiment. In all soils, quantitative PCR-based estimates of fungal biomass were highest in the forest floor. Fungal richness, based on pyrosequencing of the fungal ribosomal large subunit gene, increased in response to N-fertilization in 0–2 cm and forest floor intervals. Composition shifted in forest floor, 0–2 and 2–5 cm intervals in response to N-fertilization, but the shift was most distinct in the 0–2 cm interval, in which the largest number of statistically significant changes in soil chemical parameters (i.e., phosphorus, organic matter, calcium, pH) was also observed. In the 0–2 cm interval, increased recovery of sequences from the Thelephoraceae, Tricholomataceae, Hypocreaceae, Clavicipitaceae, and Herpotrichiellaceae families and decreased recovery of sequences from the Amanitaceae correlated with N-fertilization. In this same depth interval, Amanitaceae, Tricholomataceae, and Herpotriciellaceae sequences were recovered less frequently from soils exposed to eCO_2_ relative to ambient conditions. These results demonstrated that vertical stratification should be taken into consideration in future efforts to elucidate environmental impacts on fungal communities and their feedbacks on ecosystem processes.

## Introduction

In forested ecosystems, fungi dominate soil biomass and carry out essential biogeochemical processes (Ehrlich, 2006). Saprotrophic fungi are prolific degraders of plant material deposited to surface soils as litter and root biomass. Mycorrhizal fungi, which associate with roots of greater than 86% of plant species (Tedersoo et al., 2010), are abundant in forest ecosystems where they participate in decomposition of organic matter (OM) and transfer nitrogen and phosphorus to their plant partners.

Atmospheric carbon (C) and nitrogen (N) inputs to terrestrial ecosystems may be mediated through direct changes in the soil chemical conditions that impact microbial activities and through altered plant growth that changes the amount or quality (e.g., C:N ratio) of plant-derived substrates deposited on or in the soil. The first is intimately tied to both substrate availability and soil nutrient status (e.g., Fitter et al., 2000; Treseder and Allen, 2000; Zak et al., 2006; Parrent and Vilgalys, 2009; Edwards et al., 2011). Changes in plant biomass and physiology that occur under elevated atmospheric CO_2_ (eCO_2_) indirectly impact soil fungal communities (Treseder, 2005; Chung et al., 2006), by increasing plant productivity (up to 30%, Poorter, 1993) and altering the amount and quality of carbon inputs to soil in the form of plant litter and root exudates (e.g., Zak et al., 1993; Couteaux et al., 1995; Weatherly et al., 2003; Hall et al., 2005; Parsons et al., 2008; Phillips et al., 2009). Simultaneously, rates of N-deposition to terrestrial ecosystems are increasing (e.g., Neff et al., 2002a; Holtgrieve et al., 2011), which, alone, is predicted to increase primary productivity of the N-limited forests in the Northern Hemisphere. As with eCO_2_, N-deposition indirectly impacts soil microbes both directly (e.g., Ramirez et al., 2010) and indirectly *via* changes in plant growth and physiology (e.g., Rastetter et al., 1991).

Prior studies have provided mixed results, demonstrating the complexity of soil community responses to eCO_2_ or N-deposition alone or combined (Hungate et al., 1997; Billings and Ziegler, 2005, 2008; Janssens et al., 2010). For instance, in forested ecosystems, increased N-deposition is predicted to increase soil C-sequestration by slowing degradation of recalcitrant carbon; a mechanism postulated for this is repression of fungal genes encoding ligninolytic enzymes (Kowalenko et al., 1978; Bowden et al., 2004; Waldrop et al., 2004; Edwards et al., 2011; Fornara and Tilman, 2012). However, other studies demonstrate that N-deposition stimulates microbial activity and is associated with decreased soil C-sequestration (e.g., Khan et al., 2007; Allison et al., 2009). Shifts in soil fungal community composition in response to eCO_2_ or N-deposition in forests have been documented (Parrent et al., 2006; Allison et al., 2010, Edwards et al., 2011; Weber et al., 2011) but the nature of the identified shifts has been influenced by the approach and remains ambiguous. For example, in response to chronic N-deposition, an increase in relative abundance of Ascomycota ITS sequences in small clone libraries was reported in a boreal forest (Allison et al., 2010), but an increase in relative abundance of Basidiomycota functional gene sequences was reported in a maple forest (Edwards et al., 2011). Such discrepancies among the above studies illustrate the difficulty in ascribing mechanisms underlying field observations. It is increasingly evident that altered C and/or N conditions in the soil affect community composition and activities *via* changes in soil chemical and/or physical properties that may alter activities and competitive dynamics between species (e.g., Zhang and Han, 2012; Eisenlord et al., 2013); however, the explicit mechanisms underlying these processes remain to be fully understood. A first step in deciphering such mechanisms is to determine how soil physical and chemical properties and fungal communities have changed in response to altered C and N inputs to terrestrial ecosystems.

Defining community responses to changing C and N regimens and deciphering the underlying mechanisms is complicated by the spatially heterogeneous distribution of fungi in forest soils (e.g., Weber et al., 2011; Davison et al., 2012). The scale at which fungal taxa colonize litter and soil differs tremendously; some fungi form extensive hyphal networks that extend horizontally for meters, while others remain localized to small niches within the soil. The physical and chemical conditions in forest soils differ greatly across shallow (a few centimeters) depth intervals and fungal community composition is stratified accordingly with distinct functional groups occupying the forest floor and underlying organic (O) and mineral (A) soil horizons (Neville et al., 2002; Fierer et al., 2003; Oehl et al., 2005; O’Brien et al., 2005; Jumpponen et al., 2010). Although it is conceivable that the physical and chemical parameters of the various soil horizons as well as the fungal communities harbored within them are differentially impacted by altered C and N regimens, most studies that have examined such phenomena have homogenized soil samples across the upper 10–15 cm or deeper (e.g., Janus et al., 2005; Lipson et al., 2005; Lesaulnier et al., 2008; Castro et al., 2010). This practice may mask different responses occurring in different shallow horizons in which local soil geochemistry varies.

In this study, we tested the hypothesis that soil physical and chemical parameters in shallow soil horizons would be differentially influenced by eCO_2_ and N-fertilization, and that the resident fungal communities in those horizons would respond differently to the experimental factors. We conducted a replicated pyrosequencing investigation of the fungal ribosomal large subunit (LSU) gene, quantitative PCR (qPCR) of the fungal ribosomal small subunit (SSU) gene, and soil physical and chemical analyses across four depth intervals in a U.S. Department of Energy Free-Air Carbon Dioxide Enrichment (FACE) site in a loblolly pine forest (NC, USA).

## Materials and Methods

### Site description and sample collection

All soil cores were collected from a FACE site located in a *Pinus taeda* L. plantation in the Blackwood Division of the Duke Forest (Chapel Hill, NC, USA), which was established in 1983 (McCarthy et al., 2010). The soil is an acidic clay loam of moderately low fertility in the Enon series (McCarthy et al., 2010). Circular experimental FACE plots (30 m diameter) received fumigation with eCO_2_ (571 ppm or about 200 ppm above ambient) from August 1996 to October 2010; control plots received fumigation with ambient air during this same time interval. In 2005, the plots were quartered and annual N-fertilization began in two randomly selected quadrants in the form of ammonium nitrate pellets (11.2 g N m^−2^)^1^. Soil sampling in this study was constrained by the design of the longer-term field experiment and the desire to minimally disturb soils in the FACE plots. Therefore soil sampling was focused within the fertilized and unfertilized quadrants of a pair of FACE plots that were positioned within the same soil block based on soil physical and chemical parameters.

In July 2010, three 10 cm soil cores were collected from an N-fertilized and an unfertilized quadrant within an eCO_2_ FACE plot (plot number three) and an N-fertilized and an unfertilized quadrant within an ambient CO_2_ FACE plot (aCO_2_; plot number five), resulting in 12 cores across the four CO_2_ and N-fertilization conditions. Immediately after collection, each core was sectioned into the forest floor (the upper fraction of the O horizon, technically denoted the Oi horizon, ∼4 cm deep), 0–2 cm (the lower fraction of the O horizon, technically denoted the Oa horizon), 2–5 cm (upper A horizon), and 5–10 cm (lower A horizon) soil intervals. Each of the 48 soil fractions was homogenized in individual zip-top bags. A representative sample was removed from each bag and placed into a 50 ml Falcon tube, which was then immediately frozen in liquid nitrogen. Frozen soil samples were transported back to the laboratory on dry ice and stored at −70°C. Soils remaining in the bags were tightly sealed and transported back to the laboratory under ambient conditions for soil chemistry analysis and water content measurement (described below).

### Soil physical and chemical analyses

Percent water content was determined in each of the 48 soil samples by drying each soil fraction under ambient laboratory conditions until they stopped losing mass (about 3 days). Soil pH; total OM; nitrate and phosphorus (ppm); the cations calcium, sodium, and magnesium (meq/l), were determined in each of the 48 soil samples using standard protocols by the Soil Water and Air Testing Laboratory of New Mexico State University (Las Cruces, NM, USA).

### Nucleic acid extractions

A 10 g subsample from each of the 48 flash-frozen soil samples was individually ground in liquid nitrogen using a mortar and pestle and stored at −70 to −80°C prior to nucleic acid extraction. DNA was extracted from 0.5 g of soil using the FastDNA SPIN Kit (MP Biomedicals, LLC; Solon, OH, USA) according to the manufacturer’s instructions. DNA extracts were further purified using the MoBio PowerSoil DNA Isolation Kit (MoBio Laboratories, Inc., Solana, CA, USA) using steps 14–22 of the manufacturer’s protocol (PowerSoil DNA Isolation Kit, MoBio Laboratories, version 05182007), which are designed to remove humic acids from the DNA extracts. Final DNA concentrations and 260/280 ratios were determined using the Nanodrop 2000c (Thermo Scientific, Wilmington, DE, USA).

### Fungal SSU rRNA quantitative polymerase chain reaction

Quantitative PCR (qPCR) of the fungal SSU rRNA gene was performed in triplicate on each of the 48 DNA extracts using SYBR green detection and primers nu-SSU-1196-F and nu-SSU-1536-R under the conditions described by Castro et al. (2010). The primers utilized capture Ascomycota, Basidiomycota, Chytridiomycota, and Zygomycota, but not related non-fungal members of the oomycetes (Borneman and Hartin, 2000). Reactions were carried out in a total of three 96-well plates using triplicate sets of the same standards each time to ensure that plate-to-plate variability was minimized. Standards were generated by amplifying a fragment of the fungal SSU rRNA gene from one soil sample, cloning into *E. coli* using the TOPO TA Cloning Kit (Invitrogen, Carlsbad, CA, USA) and randomly selecting a clone. The plasmid containing the SSU rRNA gene was extracted from a Luria Broth culture (50 μg ml^−1^ carbenicillin) using the Qiaquick Plasmid Prep Kit (Qiagen, Valencia, CA, USA) according to the manufacturer instructions. Plasmid DNA (1 μg) was digested with *Sca*I (New England Biolabs, Ipswich, MA, USA) and subsequently quantified with a Nanodrop 6000c (Thermo Scientific, Wilmington, DE, USA). A dilution series of the plasmid in TE buffer ranging in copy number from 5 × 10^2^ to 5 × 10^8^ was used to standardize each set of reactions. For each of the runs, standard curves were plotted as Ct vs. log of the calculated gene copy number. Ct values for standards ranged from 10 to 34 and *R*-squared values for linear regression of the three standard curves ranged from 0.994 to 0.997. Gene copy numbers were calculated per ng DNA and per gram of dry weight (gdw) of soil.

### PCR of a fragment of the fungal LSU gene and pyrosequencing of amplicons

From each of the 48 DNA extracts, a fragment of LSU was PCR-amplified in triplicate using primers that were a concatenation of bead-adaptor sequences for the 454 GS FLX Titanium Sequencing Platform (454 Life Sciences, Branford, CT, USA) and the gene-specific primer sequences LR 3 (5′-CCGTGTTTCAAGACGGG-3′) and LR0R (5′-ACCCGCTGAACTTAAGC-3′)^2^. Each of the reverse primers also contained one of 24-unique, 5-bp barcode, or MID sequences between the adaptor sequence and gene-specific primer sequence (Table S1 in Supplementary Material). Gene fragments were amplified in 25 μl reactions containing the following: 1 × PCR Buffer (Invitrogen, Carlsbad, CA, USA), 0.2 mM dNTPs, 0.05 μM LR0R primer, 0.05 μM LR3 barcoded-primer, 15 μg of Bovine Serum Albumin (Roche Diagnostics, Indianapolis, IN, USA), 1.5 mM MgCl_2_, 1.5 U Invitrogen Taq Polymerase and 10 ng of template DNA. Reactions were carried out in an Eppendorf Mastercycler Pro (Eppendorf North America, Hauppauge, NY, USA) with the following program: initial denaturation at 95°C for 3 min, followed by 30 cycles of 95°C for 1 min, 55°C for 1 min and 72°C for 1 min, and a final extension at 72°C for 10 min.

Triplicate PCR products from each of the 48 DNA extracts were visualized on a 1% TBE agarose gel stained with ethidium bromide, pooled, and purified using the Qiaquick PCR Cleanup Kit (Qiagen, Valencia, CA, USA). The 48-amplicon pools were then combined into three larger pools for sequencing; each sequencing pool contained equal masses (about 75 ng per amplicon) of each of 16 amplicons. The three sequencing pools were sequenced on three separate halves of pico titer plates on the 454 GS FLX Titanium Sequencing Platform; this enabled use of the same set of barcodes for each set of replicate fractions (i.e., each PCR product from the replicate soil cores for a given depth interval and field treatment were labeled with the same barcode, but were sequenced on different pico titer plate halves).

### Sequence analysis

Sequences were checked for quality and parsed into 48 libraries using trim.seqs command in the mothur software package (Schloss et al., 2009) and searching only for exact matches to the respective bar codes and gene-specific primer sequences. Sequences having average quality scores <25, homopolymers >7 bases, lengths <200 bp, or with length >400 bp were removed from the datasets. Libraries were aligned against the Silva LSU database using the webserver^3^. Alignments were imported into the mothur software package (Schloss et al., 2009), where the remainder of the analyses described below were completed except where noted. Chimeric sequences were identified and removed using the pintail algorithm using aligned LSU sequences from the AFTOL database^4^ as a reference alignment. Positions in the alignment having gaps in every sequence were eliminated using the filter.seqs command. Unique sequences were then identified and identical sequences not aligned over the same region were removed from the data set prior to pre-clustering the sequences. Column-formatted distance matrices were created for operational taxonomic unit (OTU)-based analyses and normalized datasets were compared. Representative sequences of unique OTUs were classified using the naïve Bayesian classifier for fungal LSU developed by Liu et al. (2012), which is accessible on the Ribosomal Database Project website^5^. Compositional shifts in response to field treatment were displayed at the phylum and family levels based on percent composition of total sequence numbers in each library using agglomerative hierarchical clustering analysis performed in *R*^6^ based on the Bray–Curtis distance metric (taking relative abundance into account).

### Statistical analyses

Statistical analyses were performed using the JMP Statistical Discovery Software version 5.1 (SAS, Cary, NC, USA). Initial analysis of variance (ANOVA) analyses showed that differences due to the depth variable were much greater than the other two treatment effects. For this reason, and with the exception of soil nitrate concentration, depth comparisons for each soil chemical or physical variable, and for SSU rRNA gene copy number and sequence richness, were conducted using values averaged across the treatments (*n* = 12 per depth interval). Comparison of treatment means (for measurements having *F* test statistics of *p* = 0.05 or less) was conducted using Tukey’s HSD test. Within each soil depth interval, the effects of eCO_2_, N-fertilization, and the combination on soil chemical and physical measures (*n* = 3 spatial field replicate observations for each measure at each soil depth interval), were tested using ANOVA with a full factorial model [(CO_2_) × (N) × (CO_2_ × N)]. For each soil chemical or physical measure showing an ANOVA *F* test of *p* = 0.10 or less, the least square means student’s *t*-test (effects test) having at least 95% confidence (*p* = 0.05) is presented in the tabulated results. For the forest floor and 0–2 cm soil intervals, the same factorial model and pairwise *t* tests were used to test for differences in relative abundance of families within the Ascomycota and Basidiomycota.

## Results

### Soil physical and chemical properties differed across the four soil horizons

Table 1 shows the average values for each measured soil parameter for each depth in each of the four experimental treatments (*n* = 3 spatial replicates per treatment per depth interval). For all treatments except N-fertilization alone, the soil pH generally increased with depth interval, from ca. 4 to 5. OM and phosphorus contents were between four and five times higher in the forest floor than in the lower depth intervals (Table 1; *n* = 12 per depth interval, averaged across treatments, ANOVA *p* < 0.0001 for each variable measure). Similarly, water content was statistically higher (2.5–3 times) in the forest floor than the underlying soil fractions examined, where water content did not differ (Table 1; *n* = 12 per depth interval, averaged across treatments, ANOVA *p* < 0.0001). Sodium was significantly lower in the forest floor than in the 0–2 cm depth interval, which was not significantly different than the 2–5 and 5–10 cm depth intervals (Table 1; *n* = 12 per depth interval, averaged across treatments, ANOVA *p* < 0.0096). In contrast, potassium was between 9 and 20 times higher in the forest floor than in the underlying depth intervals (Table 1; *n* = 12 per depth interval, averaged across treatments, ANOVA *p* < 0.0001). Magnesium and calcium content did not vary significantly across depth intervals (Table 1).

**Table 1:** **Soil chemical or physical measurements**.

Soil depth interval	Experiment treatment	NO_3_ (ppm)	pH	Organic matter (%)	Water content (%)	P (ppm)	Ca (meq/l)	Na (meq/l × 10)	K (ppm)	Mg (meq/l)
FF	aCO_2_	0.9 (0.4)	4.07 (0.23)	38.7 (16.5)	45.5 (4.1)	39.3 (12.3)	3.5 (0.4)	3.4 (0.6)	208.3 (64.7)	1.3 (0.2)
	eCO_2_	0.9 (0.2)	3.87 (0.15)	39.8 (9.6)	43.5 (1.8)	37.4 (16.5)	2.5 (0.6)	4.6 (1.7)	121.7 (17.0)	1.3 (0.3)
	N fert	65.1 (77.1)	4.40 (0.28)	39.5 (4.3)	37.1 (3.5)	40.5 (2.8)	4.9 (1.3)	3.1 (0.3)	91.5 (46.0)	1.2 (0.0)
	eCO_2_ + N fert	18.6 (10.6)	3.50 (0.17)	49.5 (6.7)	39.7 (7.8)	33.6 (8.8)	2.3 (0.9)	5.3 (0.5)	99.3 (4.5)	1.0 (0.2)
0–2 cm	aCO_2_	0.6 (0.3)	4.60 (0.10)	4.2 (0.3)	14.4 (1.8)	2.6 (0.5)	2.4 (0.2)	6.0 (1.2)	22.0 (3.0)	0.8 (0.1)
	eCO_2_	3.4 (3.9)	4.47 (0.15)	3.8 (0.5)	16.0 (0.6)	3.2 (0.4)	2.2 (0.3)	6.4 (0.9)	16.3 (4.9)	1.0 (0.1)
	N fert	32.6 (40.7)	4.40 (0.14)	6.8 (0.6)	12.6 (0.9)	5.7 (0.1)	5.4 (2.8)	6.6 (0.1)	25.5 (2.1)	1.1 (0.5)
	eCO_2_ + N fert	27.3 (6.9)	4.10 (0.0)	4.6 (0.5)	15.0 (0.5)	2.9 (0.9)	4.1 (0.5)	4.6 (1.0)	19.7 (3.1)	1.1 (0.2)
2–5 cm	aCO_2_	0.2 (0.0)	5.30 (0.20)	3.2 (1.0)	12.7 (1.4)	1.8 (0.2)	2.4 (0.1)	3.9 (0.3)	13.0 (2.7)	0.9 (0.1)
	eCO_2_	1.2 (0.6)	4.93 (0.21)	2.5 (0.4)	15.1 (1.0)	2.4 (0.3)	2.1 (0.1)	4.3 (0.8)	10.0 (1.0)	1.0 (0.0)
	N fert	25.1 (24.6)	4.37 (0.35)	2.8 (0.1)	11.9 (3.4)	2.5 (0.6)	4.3 (3.1)	4.9 (0.3)	13.7 (3.8)	1.0 (0.5)
	eCO_2_ + N fert	27.3 (18.3)	4.63 (0.32)	2.7 (0.2)	14.1 (0.0)	2.2 (0.2)	4.3 (1.5)	4.7 (1.4)	12.3 (2.3)	1.1 (0.4)
5–10 cm	aCO_2_	0.2 (0.0)	5.67 (0.12)	1.7 (0.5)	12.6 (1.5)	1.5 (0.4)	2.9 (0.6)	5.1 (1.2)	10.7 (2.5)	1.0 (0.1)
	eCO_2_	1.0 (0.2)	5.17 (0.06)	1.6 (0.1)	13.6 (0.7)	2.0 (0.8)	1.7 (0.2)	4.8 (1.9)	6.7 (1.5)	0.7 (0.1)
	N fert	19.0 (14.4)	4.53 (0.31)	2.2 (0.3)	10.9 (2.1)	2.4 (0.7)	3.9 (2.5)	4.8 (1.3)	11.7 (3.1)	0.9 (0.4)
	eCO_2_ + N fert	18.7 (14.9)	4.93 (0.31)	4.0 (4.2)	10.9 (0.1)	1.0 (0.5)	4.0 (1.8)	3.4 (0.6)	7.0 (1.0)	1.0 (0.5)
**AVERAGED ACROSS TREATMENTS**										
FF		na	3.92 c (0.37)	42.1 a (10.3)	42.2 a (4.9)	37.4 a (10.4)	3.2 (1.2)	4.21 b (1.25)	133.7 a (59.5)	1.2 (0.2)
0–2		na	4.39 b (0.22)	4.7 b (1.2)	14.7 b (1.5)	3.4 b (1.2)	3.3 (1.6)	5.82 a (1.16)	20.5 b (4.5)	1.0 (0.2)
2–5		na	4.81 ab (0.27)	2.8 b (0.5)	13.5 b (2.1)	2.2 b (0.4)	3.3 (1.8)	4.46 b (0.82)	12.3 b (2.7)	1.0 (0.3)
5–10		na	5.08 a (0.47)	2.4 b (2.1)	12.0 b (1.7)	1.9 b (0.6)	3.1 (1.3)	4.54 ab (1.33)	9.0 b (3.0)	0.9 (0.3)
	ANOVA F test(p value)		0.0001	0.0001	0.0001	0.0001	0.9883 (nsd)	0.0096	0.0001	0.087 (nsd)

*Values are means (standard deviation) of three replicate field measurements. Comparisons between depth intervals for each measurement are shown in the lower section of the table, where the values averaged across treatments are means (standard deviation) of 12 field measurements. The ANOVA F test statistic is listed below the comparison groups and values within a comparison group followed by the same letter were not significantly different (p < 0.05) by Tukey’s HSD test. “FF” denotes forest floor*.

**Table 2:** **Changes in soil chemical and physical measures after long-term elevated atmospheric CO_2_ conditions, N-fertilization or the combination of treatments, at four soil depth intervals**.

Soil depth interval	Soil factor	ANOVA *F* test	Effects of CO_2_	Effects of N-fertilization	CO_2_ × N fert interaction
FF	Nitrate (ppm)	0.2546	…	↑ (0.0646)	…
	pH	0.0319	↓ (0.0071)	…	(0.0451)
	Organic matter (%)	0.7959	…	…	…
	Water content (%)	0.1680	…	…	…
	P (ppm)	0.7699	…	…	…
	Ca (meq/l)	0.0885	↓ (0.0148)	↑ (0.0094)	…
	K (meq/l)	0.0891	…	↓ (0.0418)	…
0–2 cm	Nitrate (ppm)	0.1944	…	↑ (0.2270)	…
	pH	0.0183	↓ (0.0245)	↓ (0.0091)	…
	Organic matter (%)	0.0016	↓ (0.0028)	↑ (0.0006)	…
	Water content (%)	0.0487	↑ (0.0154)	…	…
	P (ppm)	0.0077	↓ (0.0314)	↑ (0.0087)	(0.0038)
	Ca (meq/l)	0.0721	…	↑ (0.0094)	…
	K (meq/l)	0.2359	…	…	…
2–5 cm	Nitrate (ppm)	0.1463	…	↑ (0.0228)	…
	pH	0.0167	…	↓ (0.0037)	(0.0649)
	Organic matter (%)	0.3501	…	…	…
	Water content (%)	0.2442	…	…	…
	P (ppm)	0.2513	…	…	…
	Ca (meq/l)	0.4084	…	…	…
	K (meq/l)	0.5252	…	…	…
5–10 cm	Nitrate (ppm)	0.0952	…	↑ (0.0150)	…
	pH	0.0016	…	↓ (0.0004)	(0.0047)
	Organic matter (%)	0.7071	…	…	…
	Water content (%)	0.2123	…	…	…
	P (ppm)	0.5932	…	…	…
	Ca (meq/l)	0.3446	…	…	…
	K (meq/l)	0.1108	…	…	…

*Values in each row are the *p* value from the factorial ANOVA *F* test, followed by the direction of the effect and the least square means pairwise *t* text *p* value for that factor. Treatment effects are only shown for ANOVA *F* test statistics where *p* ≤ 0.10, and the treatment effect was *p* ≤ 0.05. Although not significantly different in the top three soil depth intervals, nitrate analysis is shown since this was an experimentally manipulated variable*.

### Soil physical and chemical properties were altered by eCO_2_ and N-fertilization within soil horizons

The impact of soil horizon on soil physical and chemical parameters was generally greater than that of experimental treatment, but statistically significant differences in some parameters were observed between experimental treatments (Table 2). Significant effects of eCO_2_ were detected only in the forest floor and in the 0–2 cm depth interval (ANOVA *F* test values and *p* values for pairwise least square means student’s *t*-tests in Table 2). In the forest floor, pH and calcium content were significantly lower under eCO_2_ conditions compared to ambient conditions. In the 0–2 cm depth interval, pH, OM, and phosphorus were significantly reduced, and water content was significantly increased under eCO_2_ conditions compared to ambient conditions.

As expected, nitrate levels in all soil depth intervals were numerically higher in N-fertilized plots (means 18.6–65.1 ppm, Table 1) than in unfertilized plots (means 0.2–3.4 ppm, Table 1). Variability among field replicate samples was surprisingly high for the nitrate measurement, which was a manipulated variable in this field experiment, and differences were only noted in the 5–10 cm depth interval (ANOVA *p* = 0.0952; Table 2). Soil pH in the 0–2, 2–5, and 5–10 cm soil intervals decreased significantly with N-fertilization. Calcium content increased with N-fertilization in the forest floor and 0–2 cm interval, and potassium content decreased with N-fertilization in the forest floor relative to unfertilized controls (Tables 1 and 2). Within the 0–2 cm depth interval, OM content was greater in N-fertilized than in unfertilized soils. Sodium and magnesium contents were the only two measured soil properties that did not differ among treatments in any of the depth intervals (means in Table 1, not included in Table 2).

### Relative fungal biomass differed with soil horizon and N-fertilization but not with eCO_2_ conditions

**Figure 1:**
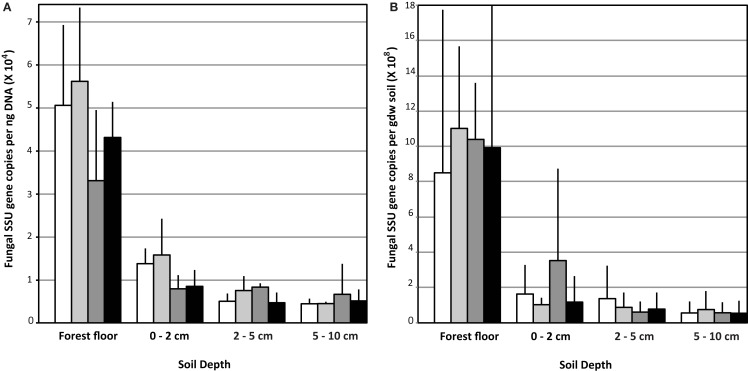
**Fungal ribosomal small subunit gene copy number: Data are represented as the mean copy number (*n* = 9) with the standard deviation per ng DNA (A) and per gram dry weight (gdw) (B)**.

Copy numbers of fungal SSU rRNA gene per ng extracted soil DNA (Figure 1A) or per gdw (Figure 1B) were significantly greater in the forest floor than in lower depth intervals (Figure 1; *n* = 12 per depth interval, averaged across treatments, ANOVA *p* < 0.0001). Values measured in the forest floor were highly variable. No impact of eCO_2_ on relative copy numbers in any of the soil horizons was detected (Figure 1). In the forest floor and the 0–2 cm depth intervals, gene copy number per ng DNA in the N-fertilized soils was lower than in the unfertilized soils. This trend was marginally significant (ANOVA *p* = 0.08) in the 0–2 cm depth interval (Figure 1; *n* = 6 N-fertilized vs. *n* = 6 unfertilized samples, However, gene copy number normalized per gdw was not significantly different among treatments in the 0–2 cm depth interval (data not shown).

### Fungal community richness was affected by soil horizon and N-fertilization

A total of 283,136 pyrotag sequences meeting the length and quality criteria described in the Materials and Methods were obtained from the 48 forest floor and soil fractions (Table 3). The pyrotag libraries generated for the 2–5 cm soil fractions from the eCO_2_ treatment were excluded from comparative analyses because of poor sequence yields (average number of sequences per library = 322; Table 3). The number of sequence reads in the libraries for the other 45-forest floor and soil fractions averaged 6,270 (Table 3).

**Table 3:** **Average number of fungal LSU sequences, (followed by the standard deviation, *n* = 3), generated from each experimental sample**.

Experimental treatment	Soil depth interval	Number sequences
aCO_2_	Forest floor	7076 (1025)
aCO_2_	0–2 cm	8448 (929)
aCO_2_	2–5 cm	6995 (390)
aCO_2_	5–10 cm	5858 (1420)
eCO_2_	Forest floor	8289 (1409)
eCO_2_	0–2 cm	6078 (883)
eCO_2_	2–5 cm	322 (44)*
eCO_2_	5–10 cm	6804 (2110)
aCO_2_ + Nfert	Forest floor	5732 (1422)
aCO_2_ + Nfert	0–2 cm	4415 (755)
aCO_2_ + Nfert	2–5 cm	5105 (803)
aCO_2_ + Nfert	5–10 cm	5517 (1325)
eCO_2_ + Nfert	Forest floor	6363 (439)
eCO_2_ + Nfert	0–2 cm	5459 (1836)
eCO_2_ + Nfert	2–5 cm	6341 (657)
eCO_2_ + Nfert	5–10 cm	6193 (2816)

*Due to low sequence yields, this treatment was not included in the data analyses.

Each pyrotag library contained ≥900 OTUs based on an OTU definition of ≥97% sequence similarity and normalization of all libraries to 3,900 sequences. Across the treatments, richness was significantly lower (*n* = 12 observations per factor in each depth interval, ANOVA *F* test *p* = 0.0017, Tukey’s HSD test) in the 0–2 cm fraction (1004 ± 133) relative to the 2–5 cm (1177 ± 114) and 5–10 cm (1237 ± 168) depth intervals. Richness in the forest floor was intermediate (1102 ± 137) and did not differ significantly from any of the other depth intervals.

**Figure 2:**
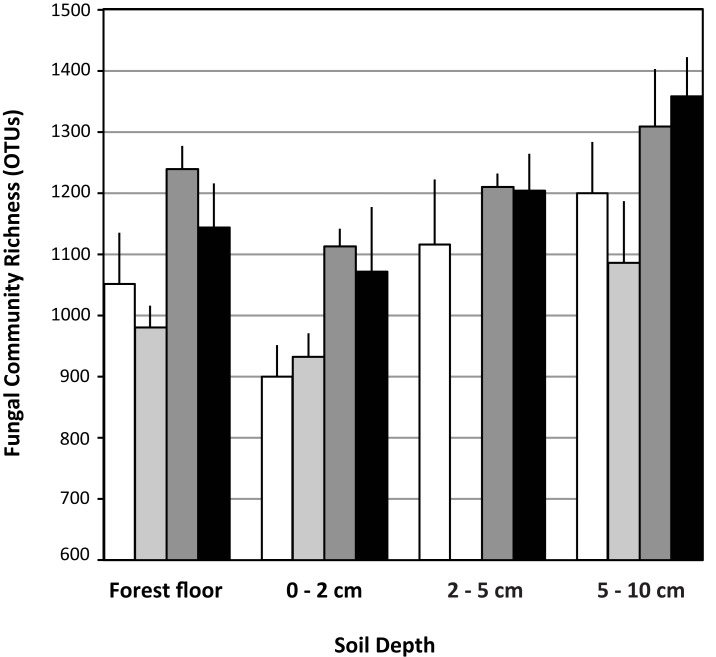
**Fungal ribosomal large subunit gene richness in libraries (OTU = 97% sequence similarity)**. Data are means with standard deviation of richness in three pyrotag libraries per field treatment for each soil depth interval examined. White bars: aCO_2_, light gray bars: eCO_2_, dark gray bars: N-fertilization, black bars: eCO_2_ + N-fertilization.

Within each depth interval, the N-fertilized soils contained an average of 180 more OTUs than the respective unfertilized fractions (Figure 2). This trend was statistically significant (*n* = 6 observations per treatment, ANOVA *F* test *p* < 0.05) in the forest floor (ANOVA least square means pairwise *t* test *p* = 0.0025) and 0–2 cm (least square means pairwise *t* test *p* = 0.0104) depth intervals (Figure 2). The trend was the same, but not significantly different for the 5–10 cm depth interval. Although the trend appeared the same for the 2–5 cm interval, conclusions cannot be drawn because of the low sequence yields from the eCO_2_ libraries at this depth and their subsequent elimination from OTU-based analyses (Table 3). In the forest floor, richness in the eCO_2_ libraries (*n* = 6 observations per treatment) was lower than that in the aCO_2_ libraries (*n* = 6, ANOVA *F* test *p* = 0.0052, least square means pairwise *t* test *p* = 0.0681).

### Basidiomycota and ascomycota were the dominant phyla present in pyrotag libraries and were differentially affected by N-fertilization

At the phylum-level, the majority of the pyrotag sequences were classified as Ascomycota and Basidiomycota (over 95% in the forest floor, reducing with depth to slightly greater than 75% at 5–10 cm depth; Table 4). Most sequences (40–94% of each library depending on depth) were classified into the Basidiomycota (Table 4). The remaining sequences were classified as Blastocladiomycota, Chytridiomycota, Glomeromycota, Neocallimastigomycota, and Zygomycota. Less than 12% of any library could not be classified at the phylum-level and were classified as Eukaryota incertae sedis or Fungi incertae sedis. The Basidiomycota families that comprised ≥1% of the libraries were mostly those known to include ectomycorrhizal fungi within the order Agaricales: Amanitaceae, Atheliaceae, Boletaceae, Cortinariaceae, Hyaloriaceae, Hysterangiaceae, Russulaceae, Sclerodermataceae, Suillaceae, Thelephoraceae, and Tricholomataceae. Members of the Tremellaceae (Basidiomycota, Tremellales) were also abundant. The dominant Ascomycota taxa observed were classified as Helotiales incertae sedis and Pezizaceae within the Pezizomycotina, and Trichomonascaceae within the Saccharomycotina. A complete listing of the Basidiomycota and Ascomycota families and their percent representation in each library is listed as Table S2 in Supplementary Material.

**Table 4:** **Proportion of Basidiomycota, Ascomycota, and Other Phyla in sequence libraries constructed from four soil depth intervals, in samples from each of four experimental treatments**.

Soil depth interval	Experimental treatment	Basidiomycota	Ascomycota	Other phyla
FF	aCO2	86.7 (6.4)	11.1 (6.0)	2.2 (0.4)
	eCO2	93.9 (3.2)	3.4 (2.2)	2.6 (1.1)
	N-fertilization	67.5 (17.6)	22.6 (9.5)	9.9 (8.1)
	eCO2 + N fert	75.4 (12.1)	20.1 (10.5)	4.5 (2.0)
	Avg. all trts treatments	80.9 (11.8)	14.3 (8.8)	4.8 (3.5)
0–2 cm	aCO2	92.7 (1.6)	5.1 (1.2)	2.3 (0.5)
	eCO2	86.5 (10.0)	5.1 (2.5)	8.4 (7.5)
	N-fertilization	64.1 (3.8)	16.3 (1.4)	19.6 (3.8)
	eCO2 + N fert	67.8 (9.3)	20.5 (5.1)	11.7 (4.2)
	Avg. all trts treatments	77.8 (14.0)	11.7 (7.9)	10.5 (7.2)
2–5 cm	aCO2	68.1 (6.2)	22.0 (5.2)	9.9 (1.7)
	eCO2	63.4 (12.5)	17.3 (6.3)	19.3 (9.1)
	N-fertilization	57.8 (10.2)	17.8 (4.8)	24.4 (6.1)
	eCO2 + N fert	47.5 (19.0)	28.7 (6.5)	23.8 (12.5)
	Avg. all trts treatments	59.2 (8.8)	21.4 (5.3)	19.3 (6.7)
5–10 cm	aCO2	63.2 (7.6)	20.2 (4.5)	16.6 (6.8)
	eCO2	60.4 (24.1)	14.6 (8.5)	25.0 (15.6)
	N-fertilization	46.3 (15.3)	28.3 (16.4)	25.4 (14.1)
	eCO2 + N fert	39.9 (17.5)	32.2 (7.2)	27.9 (10.7)
	Avg. all trts treatments	52.5 (11.2)	23.8 (7.9)	23.7 (4.9)

*Data are the mean (standard error) percent representation in three replicate sequence libraries per treatment, or 12 replicate libraries per soil depth interval (avg. all trts.)*.

Comparison of the proportion of Ascomycota and Basidiomycota in sequence libraries showed that in the forest floor and the 0–2 cm depth interval, the proportion of Ascomycota increased under N-fertilized conditions, with a corresponding decrease in Basidiomycota representation (Table 5). Statistical support for this trend was strongest (ANOVA *F* test *p* = 0.0028 and 0.0004, for Basidiomycota and Ascomycota, respectively) in the 0–2 depth interval.

**Table 5:** **Changes in proportion of Ascomycota and Basidiomycota fungi after long-term elevated atmospheric CO_2_ conditions, N-fertilization or the combination of treatments, at four soil depth intervals**.

Soil depth interval	Fungal phylum	ANOVA *F* test	Effects of CO_2_	Effects of N-fertilization	CO_2_ × N fert interaction
**FF**					
0	Ascomycota	0.0570	0.2873	0.0138 F > NF	0.5824
	Basidiomycota	0.0793	0.2765	0.0195 NF > F	0.9643
0–2 cm	Ascomycota	0.0004	0.2525	0.0001 F > NF	0.2525
	Basidiomycota	0.0028	0.7685	0.0004 NF > F	0.2695
2–5 cm	Ascomycota	0.2264	0.4553	0.3894	0.0826
	Basidiomycota	0.3483	0.3688	0.1343	0.7302
5–10 cm	Ascomycota	0.2519	0.8909	0.0701	0.4645
	Basidiomycota	0.3821	0.6706	0.1084	0.8678

*Values in each row are the *p* value from the factorial ANOVA *F* test, followed by the least square means pairwise *t* text value for that factor. F, fertilized, NF, not fertilized*.

### Fungal community composition was differentially affected by N-fertilization and eCO_2_ in soil depth intervals

**Figure 3:**
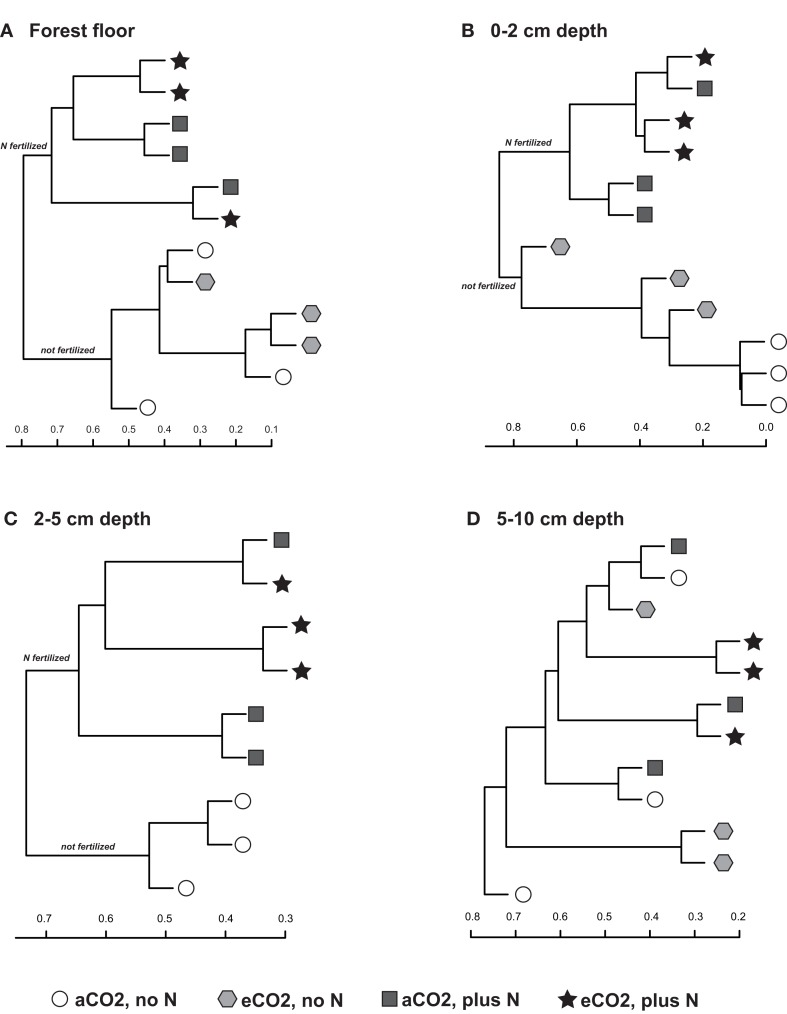
**Agglomerative hierarchical clustering dendrograms based on the Bray–Curtis distance metric (taking relative abundance into account) and the percent representation of fungal family in each library for the (A) forest floor, (B) 0–2 cm, (C) 2–5 cm, and (D) 5–10 cm depth intervals**.

Sequences classified to family level and comprising at least 1% of each replicate library for each treatment, on average, were examined for their contributions to community shifts correlating with the field treatments. Due to the difference in chemical and physical parameters across the depth profiles as well as their responses to field treatment, each depth interval was examined separately. Agglomerative hierarchical clustering dendrograms based on the family level composition (% of total sequence number in each library) detected consistent compositional responses among replicate libraries to N-fertilization in the forest floor, 0–2 and 2–5 cm depth intervals but not in the 5–10 cm depth interval (Figure 3). In the forest floor and 0–2 cm depth interval, fungal community composition shifted in response to N-fertilization under both CO_2_ conditions (Figures 3A,B). In the 2–5 cm depth interval, the family level composition of the fungal communities in N-fertilized soils was distinct from unfertilized soils under aCO_2_ (Figure 3C). In addition, community shifts correlating with eCO_2_ were noted in the 0–2 cm depth interval (Figure 3B). Among the unfertilized libraries, aCO_2_ libraries clustered closely while the composition of the eCO_2_ libraries was more variable and distinct from that of the aCO_2_ libraries (Figure 3B). No discernible response to either eCO_2_ or N-fertilization was detected in the 5–10 cm soil depth interval (Figure 3D).

**Figure 4:**
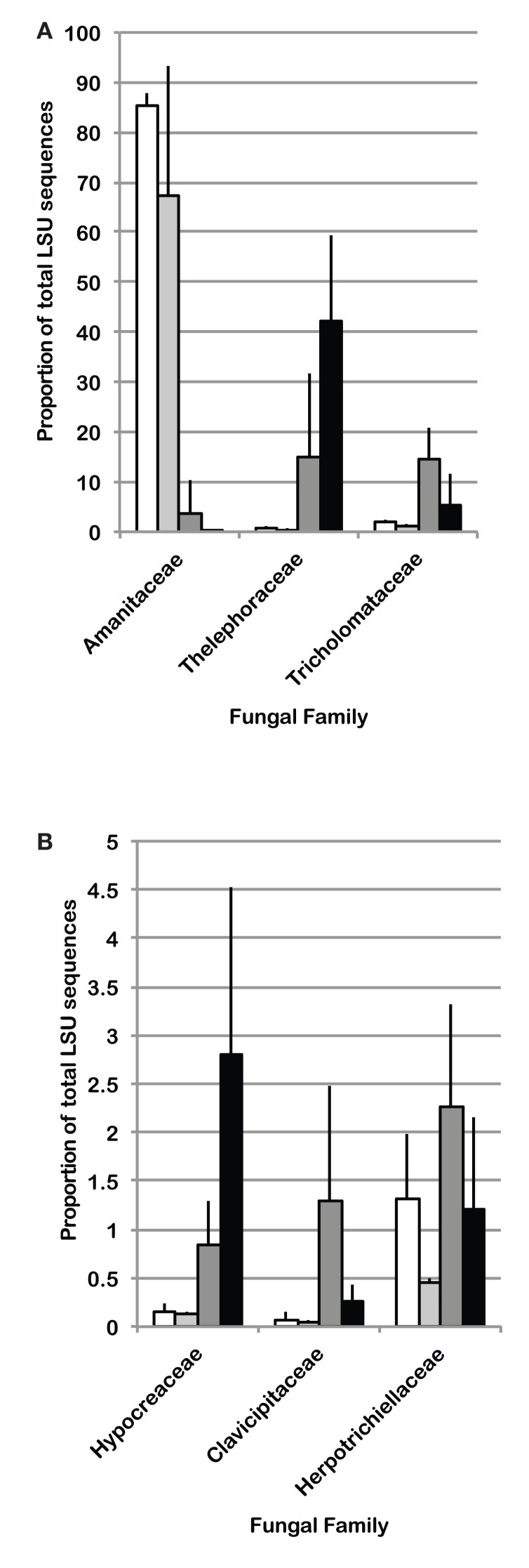
**Average percent of sequences in LSU 454 pyrotag libraries classified as (A) Basidiomycota or (B) Ascomycota, for which statistically significant differences (ANOVA *p* < 0.10 using a factorial model) were identified among field treatments in the 0–2 cm depth interval (*n* = 3)**. White bars: aCO_2_, light gray bars: eCO_2_, dark gray bars: N-fertilization, black bars: eCO_2_ + N-fertilization.

Because cohesive compositional shifts were observed among replicate 0–2 cm soil samples in response to N-fertilization and eCO_2_, and the proportional shift in Ascomycota vs. Basidiomycota was most strongly supported at the 0–2 cm depth interval (Table 5), statistical comparisons were performed on the percent composition of fungal families in this depth interval to identify potentially responsive families. Comparisons were performed on families within the Basidiomycota and Ascomycota that made up at least 1%, on average, of the sequences recovered from replicate soil samples from at least one of the four field treatments. Taxonomic comparisons were resolved at the family level where our expected identification accuracy was about 95% (Liu et al., 2012). Significant shifts and their direction are summarized in Table 6 (only families with ANOVA *F* test and least square mean pairwise *t* test *p* < 0.10 are shown), and the relative abundance of six responsive families is shown in Figure 4. The proportion of sequences classified as members of two families within the Agaricales (Thelephoraceae, Tricholomataceae), and three Ascomycota families (Hypocreaceae, Clavicipitaceae, Herpotrichiellaceae) were significantly higher in libraries generated from N-fertilized soils under both CO_2_ conditions than those generated from unfertilized control soils. In contrast, members of the Amanitaceae, which made up very large proportions of the aCO_2_ (85.2 ± 1.7%) and eCO_2_ (44 ± 15.1%) libraries from unfertilized soils, were significantly reduced in libraries generated from N-fertilized soils (Figure 4; eCO_2_ + N fert = 0.04 ± 0.02%, aCO_2_ + N fert = 3.8 ± 3.7%). Significant responses to eCO_2_ alone were evident in two families of Basidiomycota; sequence representation of the Tricholomataceae and Amanitaceae was lower in soils from eCO_2_ plots than aCO_2_ plots. The interaction term was significant for the Amanitaceae, and the significant result for eCO_2_ was strongly influenced by the negative impact of N-fertilization in this factorial analysis.

**Table 6:** **Basidiomycota and Ascomycota families showing significant differences in representation in LSU 454 pyrosequence libraries in the 0–2 cm soil depth interval, under long-term elevated atmospheric CO_2_ conditions, N-fertilization, or the combination of treatments**.

Phylum/order	Family	ANOVA *F* test	Effects of eCO_2_	Effects of N-fertilization	eCO_2_ + N fert interaction
**BASIDIOMYCOTA**					
Agaricales	Amanitaceae	0.0002	↓ (0.0214)	↓ (0.0001)	↓ (0.0444)
	Thelephoraceae	0.0081	…	↑ (0.0036)	↑ (0.0771)
	Tricholomataceae	0.0155	↓ (0.0600)	↑ (0.0079)	…
**ASCOMYCOTA**					
Sordariomycetes	Hypocreaceae	0.0218	…	↑ (0.0100)	…
	Clavicipitaceae	0.0972	…	↑ (0.0700)	…
Chaetothryiales	Herpotrichiellaceae	0.0741	↓ (0.0455)	↑ (0.0647)	…

*Values in each row are the *p* value from the factorial ANOVA *F* test, followed by the direction of the effect and the least square means pairwise *t* test value for that factor. Treatment effects are only shown where the ANOVA *F* test statistic and the treatment effect *t* test *p* value was ≤0.10*.

## Discussion

Soil physical and chemical properties differed across shallow soil horizons examined in this study (Table 1), and the observed changes in soil chemical parameters after several years of eCO_2_ or N-fertilization conditions were specific to these depth intervals and were most dramatic in the forest floor and the 0–2 cm depth intervals, while very few consistent changes occurred below 2 cm depth (Table 2). We found that the relative biomass, richness, and composition of the fungal community also differed across these shallow depth intervals, with a decrease in biomass, an increase in richness, and large compositional shifts associated with depth. Within this highly stratified soil structure, fungal community composition was differentially impacted by eCO_2_ and N-fertilization treatments. In all soil depth intervals, the fungal community was dominated primarily by Basidiomycota and secondarily by Ascomycota phyla, and the proportion of these two phyla shifted significantly between N-fertilized and unfertilized soils in the forest floor and 0–2 cm depth intervals (Table 5). In the 0–2 cm depth interval, where phylum-level shifts were greatest, we also identified responsive families that warrant further study. In summary, our study shows that natural soil chemistry and the resident fungal community are highly stratified across shallow depth gradients, and that alterations to soil chemical conditions and fungal community responses after long-term eCO_2_ and/or N-fertilization conditions are also highly stratified. These findings show that climate change-induced changes in soil conditions and biological activities are most evident in very shallow surface horizons, and imply that processes occurring at these shallow horizons may be very important for determining the direction and extent of feedbacks to climate change. They also highlight the need to examine fungal community responses to climate change parameters at finer spatial scales.

Long-term N-fertilization reduced the pH of all soil depth intervals beneath the forest floor and increased available calcium in the 0–2 cm soil depth interval. These results are consistent with prior studies where ammonium nitrate additions have been found to lower pH (Alben, 1961) and increase availability of calcium in soil (Edwards et al., 2005). Elevated CO_2_ appeared to affect soil chemical and physical parameters less than N-fertilization. However, significant reductions in pH, OM, water content and phosphorus, which correlated with the CO_2_ treatment, were identified in the 0–2 cm soil depth interval (Table 2). Opposing effects of eCO_2_ and N-fertilization were noted for calcium and phosphorus, which highlights the difficulty in predicting fungal community shifts to environmental perturbations that occur in concert.

The greatest number of statistically significant differences in physical and chemical parameters among treatments occurred in the 0–2 cm soil depth interval (Table 2), and fungal community composition differed with both the eCO_2_ and N-fertilization treatments in this depth interval (Figure 4B). Although we observed statistically significant reductions in pH with eCO_2_ treatment and several studies document this as a primary factor driving microbial community composition, most of these studies have focused on bacteria (e.g., Fierer and Jackson, 2006; Lauber et al., 2009). A more recent study that compared the responses of bacteria and fungi across a soil pH gradient ranging from 4.0 to 8.3 that controlled for other confounding factors documented significant changes in bacterial community, but not for fungi, which is consistent with culture studies (Rousk et al., 2010). Therefore, pH is not suspected as a strong driver of community structure in this study. However, it must be noted that we cannot definitively identify mechanisms for abiotic and biotic shifts in soils occurring under eCO_2_ and N-fertilization conditions. Nonetheless our findings are consistent with plant-mediated alterations and feedbacks in soil environments that have been previously noted (Hobbie, 1995). Parrent and Vilgalys (2009) previously noted that fine root biomass and mycorrhizal fungi are abundant in the top 5 cm of soil at this site; while we did not measure fine root biomass in this study and cannot make any direct correlations between fungal community composition and structure and fine root biomass, it is conceivable that the 0–2 cm interval within this zone is impacted not only by the by the quality and quantity of plant litter, but also by root exudates which may be altered in response to field treatment and have an impact on soil microbial communities (Norby et al., 1987; Zak et al., 1993; Grayston et al., 1998; Parrent and Vilgalys, 2009). For instance, Parrent and Vilgalys (2009) noted that 18S rRNA gene expression in eCO_2_ plots at this study site was higher than in the aCO_2_ plots. They hypothesized that greater C allocation to and greater metabolic activity by ectomycorrhizal fungi may have occurred, or that C was being selectively allocated to ectomycorrhizal fungi and hence greater fungal biomass associated with roots.

Fungal community responses to long-term N-fertilization were manifest broadly at the phylum-level, and included multiple families within each of the two dominant phyla. This implies a complex community shift rather than a straightforward reduction or increase in a few genera or species. Classification of fungal families contributing to fungal compositional shifts in the 0–2 cm depth interval identified some of the major responsive taxa (Figure 4). Consistent with previous studies that examined the response of soil fungal community composition to N-fertilization, we recovered increased numbers of Ascomycota sequences and decreased numbers of Basidiomycota sequences from N-fertilized soils in all depth intervals (e.g., Nemergut et al., 2008; Allison et al., 2010). This was accompanied by increased richness in N-fertilized fractions, which additionally suggests that reduction in Basidiomycota abundance may reduce some of the competitive pressures on Ascomycota for resources. Three different families within the Ascomycota (Hypocreaceae, Clavicipitaceae, Herpotrichiellaceae) increased in relative abundance under N-fertilized conditions (Table 6; Figure 4) and many more Ascomycota representatives contributed to the phylum-level composition shift. In addition to increasing the proportion of total Ascomycota, N-fertilized conditions resulted in increased richness among the Basidiomycota. Dominance by Amanitaceae sequences was replaced by a significantly increased proportion of two different Agaricales families (Thelephoraceae, Tricholomataceae; Table 6; Figure 4). Although studies show variable impacts of nitrogen fertilization on decomposition rates, in some cases rates are enhanced (Neff et al., 2002b), which would lead to increased carbon turnover. It is possible that availability of additional labile carbon and may lead to the increased abundance of certain groups of saprotrophs. These interactions are complex and warrant further study using representatives of the family groups identified as responsive in this field experiment.

In addition to the compositional changes, fungal SSU gene copy number per ng extracted DNA was significantly reduced in the 0–2 cm depth, N-fertilized soils relative to the unfertilized controls. Although copy number per cell varies among fungal species and a direct relationship between copy number and fungal biomass cannot be assumed (Fierer et al., 2005), it is possible that total fungal biomass was indeed reduced with N-fertilization. If a reduction in total fungal biomass were correlated with reduced Basidiomycota abundance, particularly for taxa encompassing mycorrhizal fungi, this would corroborate previous findings that N-fertilization adversely impacts mycorrhizal taxa at this site (Parrent et al., 2006) and others (e.g., Lilleskov et al., 2002). Evidence supporting this notion includes the observed proportion of sequences recovered from all four C and N combinations. Amanitaceae were a major fraction of the sequences in all libraries (as much as 85%), but their abundance was significantly decreased in the N-fertilized, 0–2 cm interval under both CO_2_ conditions (Figure 4). Because many of the members of the Amanitaceae are mycorrhizal, this dynamic may support the hypothesis that increased N-deposition will reduce vegetation dependence on mycorrhizae to facilitate N-uptake and plant hosts will invest less in mycorrhizal associations (Treseder, 2005). In contrast, members of the Thelephoraceae, Tricholomataceae, and Russulaceae showed the opposite trend. Not all members of these families exist solely in symbiosis with plants and may play important roles as free-living saprotrophs (Hibbett et al., 2000). This demonstrates the need for future studies to connect sequence surveys with validated, curated databases at finer taxonomic resolution (e.g., genus) that would provide information on taxa having similar lifestyles and potential ecological functions to provide deeper insights into mechanisms underlying compositional shifts in response to environmental perturbation.

The dynamics of mycorrhizal fungi may play an important role in controlling the overall richness and composition of fungal species in the soil horizons examined. In each of the CO_2_ and N-fertilization conditions, fungal richness increased with soil depth despite a decrease in biomass estimated by qPCR (Figures 1 and 2). Data presented in this study are consistent with Parrent and Vilgalys (2009), who demonstrated that mycorrhizal fungi are abundant in the upper soil horizons at the field site studied. Here, they may outcompete a diversity of fungi for resources and reduce overall richness. This notion is supported by the recent work of Lindahl et al. (2010), who examined soil fungal community composition before and after severing mycorrhizal connections with plant hosts and found that severing the mycorrhizal connections favored an increased abundance of free-living Ascomycota. This phenomenon may be happening in our field site as the presence of Basidiomycota, many of which are mycorrhizal, appear to decrease in abundance with N-fertilization while the overall richness of the fungal community increases.

A decrease in the proportion of Tricholomataceae sequences at 0–2 cm soil depth correlated with eCO_2_ conditions in the presence or absence of N-fertilization. Many members of this family are known to be ectomycorrhizal. The percent of roots colonized by ectomycorrhizal fungi (by counting ectomycorrhizal initials) has been shown to increase under eCO_2_ conditions at this field site (Garcia et al., 2008), but the fungal species contributing to that colonization increase remain unknown.

In summary, across the shallow soil horizons in this loblolly pine soil, the N-fertilization treatment exerted larger effects on the fungal communities than the eCO_2_ conditions. For both the eCO_2_ and N-fertilization treatments, the most substantial and consistent shifts in fungal community composition among replicate soil samples occurred in the 0–2 cm depth interval which indicates that community responses to environmental perturbations can be a function of soil depth at the centimeter scale, and treatment differences may have been masked in previous field studies that homogenized soils over the upper 10–15 cm (i.e., Janus et al., 2005; Lipson et al., 2005; Lesaulnier et al., 2008; Castro et al., 2010). The experimental design of future field studies to examine climate change impacts on terrestrial ecosystems should consider the differences in geochemical conditions and fungal community composition across shallow soil horizons. In the long-term, it will also be important to consider the influences of different soil types, plant cover, spatial heterogeneity, as well as seasonal patterns of plant and soil community growth, to enable accurate tracking and prediction of climate change responses in forest ecosystems.

## Conflict of Interest Statement

The authors declare that the research was conducted in the absence of any commercial or financial relationships that could be construed as a potential conflict of interest.

## Supplementary Material

The Supplementary Material for this article can be found online at: http://www.frontiersin.org/Terrestrial_Microbiology/10.3389/fmicb.2013.00078/abstract

## Footnotes

^1^http://face.env.duke.edu/fertilization.cfm; accessed in November 2012

^2^http://www.biology.duke.edu/fungi/mycolab/primers.htm

^3^http://www.arb-silva.de/

^4^http://www.aftol.org/data.php

^5^http://rdp.cme.msu.edu/

^6^http://www.r-project.org/
