# Supplementary material for: Changes in Fungal Community Composition in Response to Elevated Atmospheric CO2 and Nitrogen Fertilization Varies with Soil Horizon
Source: Front Microbiol. 2013 Apr 9;4:78. doi: 10.3389/fmicb.2013.00078 (PMC3621283; doi:10.3389/fmicb.2013.00078)
Supplement: Supplementary file 1 [file 42910_Kuske_DataSheet1.PDF]

Table SI3.
Family level classification of sequences (% library composition) using a Baysian Fungal LSU Classifier (Liu *et al.* 2012). EF= elevated CO_2, N-fertilized; E=elevated CO_2; AF=ambient CO_2, N-fertilized; A=ambient CO_2.

Family	EF 0-2 cm 1	EF 0-2 cm 2	EF 0-2 cm 3	E 0-2 cm 1	E 0-2 cm 2	E 0-2 cm 3	AF 0-2 cm 1	AF 0-2 cm 2	AF 0-2 cm 3	A 0-2 cm 1	A 0-2 cm 2	A 0-2 cm 3	F forest floor	F forest floor	F forest floor
Acarosporaceae	0.1	0.0	0.0	0.0	0.0	0.0	0.4	0.1	0.1	0.0	0.0	0.0	0.2	0.1	0.0
Acaulosporaceae	0.0	0.0	0.0	0.1	0.0	0.0	0.1	0.0	0.1	0.0	0.0	0.0	0.0	0.0	0.0
Agaricaceae	0.2	0.0	0.3	0.0	0.0	0.0	0.0	0.1	0.4	0.3	0.0	0.3	3.6	0.4	0.0
Agaricomycetes incertae sedis	0.0	0.0	0.0	0.0	0.0	0.0	0.0	0.0	0.0	0.0	0.0	0.0	0.0	0.0	0.0
Agaricostilbaceae	0.0	0.0	0.0	0.0	0.0	0.0	0.0	0.1	0.0	0.0	0.0	0.0	0.0	0.0	0.0
Agyriaceae	0.0	0.0	0.0	0.0	0.0	0.0	0.1	0.2	0.0	0.0	0.0	0.0	0.3	0.2	0.0
Albatrellaceae	0.0	0.0	0.0	0.0	0.0	0.0	0.0	0.0	0.1	0.0	0.0	0.0	0.1	0.0	0.0
Amanitaceae	0.0	0.0	0.1	52.1	65.3	14.6	11.2	0.0	0.0	83.0	88.5	84.0	3.1	0.3	0.0
Amoebidiaceae	0.2	0.1	0.4	0.2	0.0	0.0	1.9	0.2	0.4	0.0	0.1	0.1	0.1	0.1	0.0
Amphisphaeriaceae	0.0	0.0	0.0	0.0	0.0	0.0	0.0	0.0	0.1	0.0	0.0	0.0	0.0	0.0	0.0
Amplistromataceae	0.0	0.0	0.0	0.0	0.0	0.0	0.0	0.0	0.0	0.0	0.0	0.0	0.0	0.0	0.0
Annulatascaceae	0.0	0.0	0.0	0.0	0.0	0.0	0.1	0.0	0.0	0.0	0.0	0.0	0.1	0.0	0.0
Apiosporaceae	0.0	0.0	0.0	0.0	0.0	0.0	0.0	0.0	0.0	0.0	0.0	0.0	0.0	0.0	0.0
Aporpiaceae	0.0	0.0	0.0	0.0	0.0	0.0	0.0	0.0	0.0	0.0	0.0	0.0	0.1	0.0	0.0
Arachnomycetaceae	0.0	0.5	0.4	0.0	0.0	0.0	0.0	0.1	0.0	0.1	0.0	0.1	0.0	0.0	0.0
Arthoniaceae	0.0	0.0	0.0	0.0	0.0	0.0	0.0	0.0	0.0	0.0	0.0	0.0	0.0	0.0	0.1
Arthrodermataceae	0.0	0.0	0.0	0.0	0.0	0.0	0.0	0.0	0.0	0.0	0.0	0.0	0.0	0.0	0.0
Ascobolaceae	0.0	0.0	0.0	0.0	0.0	0.0	0.0	0.0	0.0	0.0	0.0	0.0	0.0	0.0	0.0
Ascodesmidaceae	0.0	0.0	0.0	0.0	0.0	0.0	0.0	0.0	0.0	0.0	0.0	0.0	0.0	0.0	0.0
Ascoideaceae	0.0	0.0	0.0	0.0	0.0	0.0	0.0	0.0	0.0	0.0	0.0	0.0	0.0	0.0	0.0
Ascomycota incertae sedis	0.0	0.0	0.4	0.1	0.0	0.0	0.7	0.4	0.3	0.6	0.1	0.0	0.8	0.7	0.6
Asterinaceae	0.0	0.0	0.0	0.0	0.0	0.0	0.0	0.0	0.0	0.0	0.0	0.0	0.0	0.0	0.0
Asterothyriaceae	0.0	0.0	0.0	0.1	0.0	0.0	0.2	0.0	0.3	0.1	0.0	0.0	0.1	0.1	0.0
Atheliaceae	0.4	0.3	0.7	0.0	0.0	0.5	0.0	0.9	0.1	1.1	0.4	0.2	4.6	8.5	0.8
Auriculariales incertae sedis	0.0	0.0	0.0	0.0	0.0	0.0	0.0	0.0	0.3	0.0	0.0	0.0	0.0	0.0	0.0
Auriscalpiaceae	0.0	0.0	0.0	0.0	0.0	0.0	0.0	0.0	0.0	0.0	0.0	0.0	0.0	0.0	0.0
Bankeraceae	0.0	0.0	0.0	0.0	0.0	0.0	0.0	0.0	0.0	0.0	0.0	0.0	0.0	0.0	0.0
Basidiomycota incertae sedis	0.0	0.0	0.0	0.0	0.0	0.0	0.0	0.0	0.0	0.0	0.0	0.0	0.0	0.0	0.0
Batistiaceae	0.2	0.4	0.1	0.0	0.0	0.0	0.1	0.1	0.0	0.0	0.0	0.0	0.0	0.0	0.0
Battarreaceae	0.0	0.0	0.0	0.0	0.0	0.0	0.0	0.0	0.0	0.1	0.0	0.0	0.0	0.0	0.0
Bertiaceae	0.0	0.0	0.0	0.0	0.0	0.0	0.0	0.0	0.0	0.0	0.0	0.0	0.0	0.0	0.0
Bionectriaceae	0.0	0.1	0.0	0.0	0.0	0.0	0.2	0.4	0.0	0.0	0.0	0.0	0.0	0.1	0.1
Blastocladiaceae	0.5	0.7	0.5	0.2	0.0	0.1	0.6	0.6	1.0	0.1	0.1	0.1	1.0	0.2	0.1
Bolbitiaceae	0.0	0.0	0.0	0.0	0.0	0.0	0.0	0.0	0.0	0.0	0.0	0.0	0.0	0.0	0.0
Boletaceae	2.0	8.9	0.3	0.1	0.2	0.5	0.0	0.0	0.3	0.0	0.1	0.1	24.9	8.8	5.7
Botryobasidiaceae	0.0	0.0	0.0	0.0	0.0	0.0	0.0	0.0	0.0	0.0	0.0	0.0	0.0	0.0	0.2
Botryosphaeriaceae	0.0	0.0	0.0	0.0	0.1	0.0	0.0	0.0	0.0	0.0	0.0	0.0	0.0	0.1	0.0
Bulgariaceae	0.0	0.0	0.0	0.0	0.0	0.0	0.1	0.1	0.0	0.1	0.0	0.1	0.0	0.0	0.0
Caloscyphaceae	0.0	0.0	0.0	0.0	0.0	0.0	0.0	0.0	0.0	0.0	0.0	0.0	0.0	0.0	0.0
Calosphaeriaceae	0.0	0.0	0.0	0.0	0.0	0.0	0.0	0.0	0.0	0.0	0.0	0.0	0.0	0.0	0.0
Candelariaceae	0.0	0.0	0.0	0.0	0.0	0.0	0.0	0.0	0.0	0.0	0.0	0.1	0.0	0.0	0.0
Cantharellaceae	0.0	0.0	0.0	0.0	0.0	0.0	0.0	0.0	0.0	0.0	0.0	0.0	0.0	0.0	0.0
Capnodiaceae	0.0	0.0	0.0	0.0	0.0	0.0	0.0	0.0	0.0	0.0	0.0	0.0	0.0	0.0	0.0
Capnodiales incertae sedis	0.0	0.0	0.0	0.0	0.0	0.0	0.0	0.0	0.0	0.0	0.0	0.0	0.1	0.0	0.1
Capnodiales incertae sedis	0.0	0.0	0.0	0.0	0.0	0.0	0.0	0.0	0.0	0.0	0.0	0.0	0.1	0.0	0.1
Catabotrydaceae	0.1	0.0	0.1	0.3	0.0	0.1	0.0	0.1	0.1	0.0	0.0	0.0	0.0	0.1	0.1
Catenariaceae	0.3	0.2	0.6	0.6	0.2	0.3	0.9	0.5	0.4	0.0	0.1	0.0	0.2	0.1	0.0
Catillariaceae	0.0	0.0	0.0	0.0	0.0	0.0	0.0	0.0	0.0	0.0	0.0	0.0	0.0	0.0	0.0
Cephaloascaceae	0.0	0.3	0.4	0.0	0.0	0.0	0.0	0.1	2.5	0.1	0.0	0.0	0.0	0.4	0.0
Cephalothecaceae	0.0	0.0	0.0	0.0	0.0	0.0	0.0	0.0	0.0	0.0	0.0	0.0	0.0	0.2	0.0
Ceratobasidiaceae	0.0	0.0	0.5	0.0	0.0	0.0	0.2	0.4	0.0	0.0	0.0	0.0	0.0	0.1	0.0
Cetradoniaceae	0.0	0.0	0.0	0.0	0.0	0.1	0.0	0.0	0.0	0.0	0.0	0.0	0.0	0.0	0.0
Chaetomiaceae	0.0	0.0	0.0	0.0	0.0	0.0	0.0	0.0	0.0	0.0	0.0	0.0	0.0	0.0	0.0
Chaetosphaeriaceae	0.1	0.1	0.1	0.0	0.0	0.1	0.2	0.1	1.0	0.0	0.1	0.0	0.1	0.1	0.1
Chaetothyriaceae	0.0	0.0	0.0	0.0	0.0	0.0	0.1	0.0	0.0	0.0	0.0	0.0	0.1	0.1	0.2
Chionosphaeraceae	0.0	0.0	0.0	0.0	0.0	0.0	0.0	0.1	0.0	0.0	0.0	0.0	0.0	0.0	0.0
Chorioactidaceae	0.0	0.0	0.0	0.0	0.0	0.0	0.0	0.0	0.0	0.0	0.0	0.0	0.0	0.0	0.0
Christianseniaceae	0.2	0.5	0.5	0.6	0.1	0.1	0.1	0.0	0.2	0.0	0.0	0.1	0.0	0.0	0.0
Chytridiaceae	1.1	2.3	0.6	0.8	0.2	0.1	1.3	1.2	1.7	0.3	0.2	0.1	1.1	0.7	0.5
Chytridiales incertae sedis	0.0	0.1	0.0	0.0	0.0	0.0	0.4	0.0	1.1	0.2	0.0	0.0	0.0	0.0	0.0
Cladoniaceae	0.0	0.0	0.0	0.0	0.0	0.0	0.1	0.0	0.0	0.0	0.0	0.0	0.0	0.0	0.0
Clathraceae	0.0	0.0	0.0	0.0	0.0	0.0	0.0	0.0	0.5	0.0	0.0	0.0	0.0	0.0	0.0
Clavariaceae	0.0	0.1	0.0	0.0	0.0	0.0	0.1	0.2	0.0	1.4	0.2	0.6	0.0	0.0	0.0
Clavicipitaceae	0.1	0.4	0.3	0.1	0.1	0.1	0.5	0.8	2.7	0.0	0.2	0.0	0.1	0.2	0.0
Clavulinaceae	0.0	0.0	0.0	0.0	0.0	0.0	2.3	0.1	0.0	0.0	0.0	0.0	0.0	0.0	0.0

Clypeosphaeriaceae	0.0	0.0	0.0	0.0	0.0	0.0	0.0	0.0	0.0	0.0	0.0	0.0	0.0	0.0	0.0
Coccodiniaceae	0.0	0.0	0.0	0.0	0.0	0.0	0.0	0.0	0.0	0.0	0.0	0.0	0.1	0.0	0.0
Codonosigidae	0.0	0.2	0.2	1.1	0.2	0.7	1.9	2.4	0.8	0.1	0.1	0.0	0.0	0.0	0.0
Coelomomycetaceae	0.2	0.4	0.8	0.6	0.1	0.3	1.1	0.6	1.5	0.1	0.1	1.1	0.3	0.6	0.2
Coleosporiaceae	0.0	0.0	0.0	0.0	0.0	0.0	0.0	0.2	0.0	0.0	0.0	0.0	0.0	0.0	0.0
Coniochaetaceae	0.1	0.0	0.0	0.0	0.0	0.0	0.1	0.0	0.0	0.0	0.0	0.0	0.0	0.0	0.0
Coniochaetales incertae sedis	0.1	0.2	0.4	0.1	0.0	0.3	0.0	0.0	0.0	0.0	0.0	0.0	0.6	0.5	0.0
Coniophoraceae	0.0	0.0	0.0	0.0	0.0	0.0	0.0	0.0	0.0	0.0	0.0	0.0	2.2	0.0	0.0
Cordycipitaceae	0.0	0.1	0.1	0.0	0.0	0.0	0.2	0.3	0.4	0.0	0.0	0.0	0.6	0.9	0.3
Coriolaceae	0.3	0.0	0.0	0.0	0.0	0.0	0.2	0.5	2.3	0.0	0.0	0.1	6.8	2.3	0.6
Coronophorales incertae sedis	0.0	0.0	0.0	0.0	0.0	0.0	0.1	0.0	0.1	0.1	0.0	0.0	0.0	0.0	0.0
Cortinariaceae	0.0	0.0	0.1	1.3	0.0	0.4	0.6	0.0	1.2	0.6	0.4	0.7	0.0	0.0	0.0
Coryneliaceae	0.0	0.0	0.0	0.0	0.0	0.0	0.0	0.0	0.0	0.0	0.0	0.0	0.0	0.0	0.0
Cronartiaceae	0.4	0.3	0.4	0.0	0.0	0.0	0.0	0.7	1.1	0.0	0.0	0.0	1.3	2.1	0.2
Cryptobasidiaceae	0.0	0.0	0.0	0.0	0.0	0.0	0.0	0.0	0.0	0.0	0.0	0.0	0.0	0.0	0.0
Cryptomycocolacaceae	0.0	0.0	0.0	0.0	0.0	0.0	0.0	0.0	0.0	0.0	0.0	0.0	0.0	0.0	0.0
Cystobasidiomycetes incertae s	0.1	0.3	0.1	0.1	0.1	0.0	0.1	0.0	0.0	0.0	0.2	0.0	0.0	0.0	0.0
Cystofilobasidiaceae	0.0	0.0	0.0	0.0	0.0	0.0	0.0	0.0	0.0	0.0	0.0	0.0	0.0	0.0	0.0
Dacrymycetaceae	0.0	0.0	0.0	0.0	0.0	0.0	0.0	0.0	0.0	0.0	0.0	0.0	0.1	0.0	0.0
Dactylosporaceae	0.0	0.0	0.0	0.0	0.0	0.0	0.2	0.0	0.0	0.0	0.0	0.0	0.0	0.0	0.0
Davidiellaceae	0.0	0.0	0.0	0.0	0.0	0.0	0.0	0.0	0.0	0.0	0.0	0.0	0.0	0.0	0.0
Dermateaceae	0.0	0.0	0.0	0.0	0.0	0.0	0.0	0.0	0.0	0.0	0.0	0.0	0.1	0.1	0.0
Diaporthales incertae sedis	0.0	0.0	0.0	0.0	0.0	0.0	0.0	0.0	0.0	0.0	0.0	0.0	0.0	0.0	0.0
Diatrypaceae	0.0	0.0	0.0	0.0	0.0	0.0	0.0	0.0	0.0	0.0	0.0	0.0	0.0	0.0	0.0
Didymellaceae	0.0	0.0	0.0	0.0	0.0	0.0	0.0	0.0	0.0	0.0	0.0	0.0	0.0	0.0	0.0
Dimargaritaceae	0.0	0.1	0.2	0.3	0.0	0.1	0.2	0.1	0.4	0.0	0.0	0.0	0.0	0.1	0.1
Dipodascaceae	0.3	0.5	0.1	0.5	0.0	0.1	1.7	0.7	0.6	0.1	0.2	0.1	0.9	0.6	0.3
Discinaceae	0.0	0.0	0.0	0.0	0.0	0.0	0.0	0.0	0.0	0.0	0.0	0.0	0.0	0.0	0.0
Doassansiaceae	0.0	0.0	0.1	0.0	0.0	0.0	0.0	0.0	0.0	0.0	0.0	0.0	0.0	0.0	0.0
Doassansiopsaceae	0.0	0.0	0.0	0.0	0.0	0.0	0.0	0.0	0.0	0.0	0.0	0.0	0.0	0.0	0.0
Dothideaceae	0.0	0.0	0.0	0.0	0.0	0.0	0.1	0.0	0.0	0.0	0.0	0.0	0.0	0.0	0.0
Dothideales incertae sedis	0.0	0.0	0.0	0.0	0.0	0.0	0.0	0.0	0.0	0.0	0.0	0.0	0.0	0.0	0.0
Dothideomycetes incertae sedis	0.0	0.3	0.2	0.3	0.1	0.0	0.3	0.3	0.2	0.2	0.0	0.0	0.3	0.7	0.3
Dothioraceae	0.0	0.0	0.0	0.0	0.0	0.0	0.0	0.0	0.0	0.0	0.0	0.0	0.0	0.0	0.0
Echinodontiaceae	0.0	0.0	0.0	0.0	0.0	0.0	0.0	0.0	0.0	0.0	0.0	0.0	0.0	0.0	0.0
Elaphomycetaceae	0.1	0.7	0.1	0.0	0.0	0.0	0.0	0.1	0.0	0.0	0.0	0.0	0.0	0.0	0.0
Elsinoaceae	0.0	0.0	0.0	0.0	0.0	0.0	0.0	0.0	0.0	0.0	0.0	0.0	0.0	0.0	0.0
Endochytriaceae	0.0	0.0	0.0	0.0	0.0	0.0	1.1	0.0	0.3	0.0	0.0	0.0	0.0	0.0	0.0
Entolomataceae	0.1	0.6	0.1	0.0	0.0	0.0	0.1	0.5	0.2	0.0	0.1	0.0	0.3	0.6	0.3
Eocronartiaceae	0.0	0.0	0.0	0.0	0.0	0.0	0.0	0.0	0.0	0.0	0.0	0.0	0.0	0.0	0.0
Erysiphaceae	0.0	0.0	0.0	0.0	0.0	0.0	0.0	0.0	0.0	0.0	0.0	0.0	0.0	0.0	0.0
Eukaryota incertae sedis	0.4	0.8	0.8	0.8	0.0	0.1	2.3	0.3	1.5	0.4	0.1	0.0	0.2	0.5	0.0
Eurotiomycetes incertae sedis	0.0	0.0	0.0	0.0	0.0	0.0	0.0	0.0	0.0	0.0	0.0	0.0	0.0	0.0	0.0
Exidiaceae	0.1	0.1	0.0	0.0	0.0	0.0	0.2	0.0	0.2	0.0	0.0	0.0	0.1	0.0	1.9
Exobasidiaceae	0.0	0.0	0.2	0.0	0.0	0.1	0.4	0.3	0.3	0.2	0.2	0.4	0.0	0.0	0.0
Fungi incertae sedis	0.0	0.0	0.0	0.0	0.0	0.0	0.2	0.3	0.4	0.0	0.0	0.2	0.0	0.0	0.0
Ganodermataceae	0.0	0.0	0.0	0.0	0.0	0.0	0.0	0.0	0.0	0.0	0.0	0.0	0.0	0.0	0.0
Gastrosporiaceae	0.0	0.0	0.0	0.0	0.0	0.0	0.0	0.0	0.0	0.0	0.0	0.0	0.0	0.0	0.0
Georgefischeriaceae	0.0	0.0	0.0	0.0	0.0	0.0	0.0	0.0	0.0	0.0	0.0	0.0	0.0	0.0	0.0
Geosiphonaceae	0.0	0.0	0.0	0.1	0.0	0.0	0.1	0.0	0.3	0.1	0.0	0.0	0.0	0.1	0.0
Gloeocystidiellaceae	0.5	0.0	0.0	0.0	0.0	0.0	0.0	0.9	0.3	0.0	0.0	0.0	0.4	0.5	0.7
Gloeophyllaceae	0.0	0.0	0.0	0.0	0.0	0.0	0.0	0.0	0.0	0.0	0.0	0.0	0.0	0.0	0.0
Glomeraceae	0.0	0.6	0.5	0.1	0.0	0.0	1.2	0.1	0.9	0.2	0.0	0.0	0.0	0.0	0.0
Glomerellaceae	0.0	0.0	0.0	0.0	0.0	0.0	0.0	0.0	0.0	0.0	0.0	0.0	0.0	0.0	0.0
Gomphaceae	0.0	0.0	0.0	0.0	0.0	0.0	0.5	0.3	0.0	0.0	0.0	0.0	0.0	0.0	0.0
Gomphidiaceae	0.0	0.0	0.0	0.0	0.0	0.0	0.0	0.2	0.0	0.0	0.0	0.0	0.0	0.0	0.0
Gomphillaceae	0.0	0.0	0.0	0.0	0.0	0.0	0.0	0.0	0.0	0.0	0.0	0.0	0.0	0.0	0.0
Gonapodyaceae	0.5	0.0	0.1	0.1	0.0	0.0	0.2	0.0	0.1	0.0	0.0	0.0	0.0	0.0	0.0
Graphidaceae	0.2	0.1	0.1	0.1	0.0	0.0	0.3	0.1	0.2	0.1	0.0	0.0	0.1	0.1	0.1
Graphostromataceae	0.0	0.0	0.0	0.0	0.0	0.0	0.0	0.0	0.0	0.0	0.0	0.0	0.0	0.0	0.0
Gyalectaceae	0.0	0.0	0.0	0.2	0.1	0.0	0.1	0.0	0.0	0.0	0.1	0.0	0.0	0.0	0.0
Gyrodontaceae	0.0	0.0	0.0	0.0	0.0	0.4	0.1	0.0	0.0	0.0	0.1	0.0	0.0	0.0	0.0
Halosphaeriaceae	0.0	0.0	0.0	0.0	0.0	0.0	0.1	0.0	0.1	0.1	0.0	0.0	0.0	0.0	0.0
Hapalopilaceae	0.0	0.0	0.0	0.0	0.0	0.0	0.0	0.0	0.0	0.0	0.0	0.0	0.0	0.0	0.0
Helicocephalidaceae	0.0	0.3	0.1	0.3	0.0	0.0	0.3	0.5	0.3	0.0	0.0	0.0	0.0	0.0	0.0
Helotiaceae	0.0	0.4	0.1	0.0	0.0	0.0	0.1	0.1	0.2	0.0	0.0	0.0	0.3	0.9	0.2
Helotiales incertae sedis	0.1	0.2	0.3	0.5	0.1	0.1	0.2	0.3	0.3	0.4	0.2	0.0	0.5	1.1	1.5
Helvellaceae	0.0	0.0	0.0	0.0	0.0	0.0	0.0	0.0	0.0	0.0	0.0	0.0	0.0	0.0	0.0
Hemiphacidiaceae	0.6	0.0	0.0	0.0	0.0	0.0	0.0	1.1	0.8	0.1	0.0	0.0	0.0	0.0	0.0
Hericiaceae	0.0	0.1	0.1	0.1	0.0	0.0	0.5	0.0	0.2	0.0	0.0	0.0	1.4	0.2	0.1
Herpotrichiellaceae	0.5	0.9	2.3	0.3	0.3	0.2	2.2	1.3	3.3	2.1	1.1	0.8	1.6	3.0	0.8

Hoehnelomycetaceae	0.0	0.0	0.0	0.0	0.0	0.0	0.0	0.0	0.0	0.0	0.0	0.0	0.0	0.0	0.0
Hyaloriaceae	0.5	0.3	2.2	0.1	0.0	0.0	0.4	1.1	5.3	0.0	0.0	0.0	0.7	0.7	2.1
Hyaloscyphaceae	0.1	0.0	0.0	0.0	0.1	0.0	0.1	0.0	0.1	0.0	0.0	0.0	0.1	0.0	0.0
Hygrophoraceae	0.0	0.0	0.0	0.0	0.0	0.0	0.0	0.0	0.0	0.0	0.0	0.0	0.0	0.0	0.0
Hygrophoropsidaceae	0.0	0.0	0.0	0.0	0.0	0.0	0.0	0.0	0.0	0.0	0.0	0.0	0.0	0.0	0.0
Hymeneliaceae	0.0	0.0	0.0	0.0	0.0	0.0	0.0	0.0	0.0	0.0	0.0	0.0	0.0	0.0	0.0
Hymenochaetaceae	0.0	0.0	0.0	0.0	0.0	0.0	0.0	0.2	0.0	0.0	0.0	0.0	2.6	0.2	0.2
Hymenochaetales incertae sedis	0.0	0.3	0.0	0.0	0.0	0.0	0.0	0.0	0.0	0.0	0.0	0.0	0.0	3.3	0.2
Hyphodermataceae	0.0	0.0	0.0	0.0	0.0	0.0	0.1	0.0	0.0	0.0	0.0	0.0	0.0	0.0	0.0
Hypocreaceae	0.8	4.1	3.4	0.2	0.1	0.1	0.3	1.2	1.0	0.1	0.2	0.1	1.6	3.9	0.7
Hypocreales incertae sedis	0.0	0.0	0.0	0.0	0.0	0.0	0.0	0.0	0.0	0.0	0.0	0.0	0.0	0.0	0.0
Hyponectriaceae	0.0	0.0	0.0	0.0	0.0	0.0	0.0	0.0	0.0	0.0	0.0	0.0	0.0	0.0	0.0
Hysterangiaceae	0.0	0.0	0.0	0.0	0.0	0.1	0.0	0.0	0.0	0.0	0.0	0.0	0.0	0.0	0.0
Hysteriaceae	0.0	0.0	0.0	0.0	0.0	0.0	0.0	0.0	0.0	0.0	0.0	0.0	0.0	0.0	0.0
Hysteriales incertae sedis	0.0	0.0	0.0	0.0	0.0	0.0	0.1	0.0	0.0	0.0	0.0	0.0	0.0	0.0	0.0
Kappamycetaceae	0.1	0.0	0.1	0.0	0.0	0.0	0.0	0.2	0.1	0.0	0.0	0.0	0.0	0.0	0.0
Lachnocladiaceae	0.0	0.0	0.0	0.0	0.0	0.0	0.0	0.0	0.1	0.0	0.0	0.0	0.0	0.0	0.0
Lasiosphaeriaceae	0.0	0.0	0.0	0.0	0.0	0.0	0.0	0.1	0.0	0.0	0.0	0.0	0.3	0.0	0.0
Lecanoraceae	0.0	0.0	0.0	0.0	0.0	0.0	0.1	0.0	0.0	0.0	0.0	0.0	0.0	0.0	0.0
Lecanoromycetes incertae sedis	0.0	0.0	0.0	0.0	0.0	0.0	0.0	0.0	0.0	0.0	0.0	0.0	0.0	0.0	0.0
Lecideaceae	0.0	0.0	0.0	0.0	0.0	0.0	0.0	0.0	0.0	0.0	0.0	0.0	0.0	0.0	0.0
Lentitheciaceae	0.0	0.0	0.0	0.0	0.0	0.0	0.0	0.0	0.0	0.0	0.0	0.0	0.0	0.0	0.0
Leotiomycetes incertae sedis	0.0	0.1	0.0	0.0	0.0	0.0	0.1	0.0	0.0	0.0	0.0	0.0	0.0	0.1	0.0
Leptosphaeriaceae	0.0	0.0	0.0	0.0	0.0	0.0	0.0	0.0	0.0	0.0	0.0	0.0	0.0	0.0	0.0
Leucogastraceae	0.0	0.0	0.0	0.0	0.0	0.0	0.0	0.0	0.0	0.0	0.0	0.0	0.0	0.0	0.0
Lichinaceae	0.0	0.0	0.0	0.0	0.0	0.0	0.0	0.0	0.0	0.0	0.0	0.0	0.0	0.0	0.0
Lipomycetaceae	0.0	0.0	0.0	0.1	0.0	0.0	0.0	0.0	0.0	0.0	0.0	0.0	0.0	0.0	0.0
Lobulomycetaceae	0.1	0.1	0.3	0.7	0.1	0.1	0.2	1.0	0.1	0.0	0.0	0.0	0.0	0.0	0.0
Lophiostomataceae	0.0	0.0	0.0	0.0	0.0	0.0	0.0	0.0	0.0	0.0	0.0	0.0	0.0	0.0	0.0
Loxosporaceae	0.0	0.0	0.0	0.0	0.0	0.0	0.0	0.0	0.0	0.0	0.0	0.0	0.0	0.0	0.0
Lycoperdaceae	0.0	0.0	0.0	0.0	0.0	0.0	0.0	0.0	0.0	0.0	0.0	0.0	0.0	0.0	0.0
Magnaporthaceae	0.0	0.0	0.0	0.0	0.0	0.0	0.1	0.0	0.1	0.0	0.0	0.0	0.5	0.3	0.3
Malasseziaceae	0.0	0.0	0.0	0.0	0.0	0.0	0.0	0.0	0.0	0.0	0.0	0.0	0.0	0.0	0.0
Marasmiaceae	0.0	0.1	0.9	0.1	0.0	0.0	0.0	0.0	0.0	0.0	0.0	0.0	0.0	0.4	0.0
Massariaceae	0.0	0.0	0.0	0.0	0.0	0.0	0.0	0.0	0.0	0.0	0.0	0.0	0.0	0.0	0.0
Massarinaceae	0.0	0.0	0.0	0.0	0.0	0.0	0.0	0.0	0.0	0.0	0.0	0.0	0.0	0.0	0.0
Megachytriaceae	0.0	0.0	0.1	0.1	0.0	0.0	0.2	0.1	0.0	0.0	0.0	0.0	0.6	0.0	0.0
Megalosporaceae	0.0	0.0	0.0	0.0	0.0	0.0	0.0	0.0	0.0	0.0	0.0	0.0	0.0	0.0	0.0
Melanogastraceae	0.0	0.0	0.0	0.0	0.0	0.0	0.0	0.0	0.0	0.0	0.0	0.0	0.0	0.0	0.0
Meripilaceae	0.0	0.0	0.1	0.0	0.0	0.0	0.3	0.0	0.1	0.7	0.0	0.0	0.0	0.0	0.0
Metschnikowiaceae	0.0	0.0	0.0	0.0	0.0	0.0	0.1	0.0	0.0	0.0	0.0	0.0	0.0	0.0	0.0
Microascaceae	0.0	0.0	0.0	0.0	0.0	0.0	0.0	0.0	0.0	0.0	0.0	0.0	0.0	0.0	0.0
Microbotryomycetes incertae se	0.7	0.4	3.7	0.4	0.9	1.2	0.1	0.2	0.0	0.1	0.2	0.3	0.6	0.8	0.0
Micropeltidaceae	0.0	0.0	0.0	0.0	0.0	0.0	0.0	0.0	0.0	0.0	0.0	0.0	0.0	0.0	0.0
Microthyriaceae	0.0	0.0	0.0	0.0	0.0	0.0	0.0	0.0	0.0	0.0	0.0	0.0	0.0	0.1	0.1
Monoblepharidales incertae sed	0.2	0.5	2.4	0.9	0.1	0.1	0.5	0.1	0.0	0.0	0.1	0.1	0.0	0.0	0.0
Montagnulaceae	0.0	0.0	0.0	0.0	0.0	0.0	0.0	0.0	0.0	0.0	0.0	0.0	0.0	0.0	0.0
Morchellaceae	0.0	0.0	0.0	0.0	0.0	0.0	0.0	0.0	0.0	0.0	0.0	0.0	0.0	0.0	0.0
Mycocaliciaceae	0.0	0.0	0.0	0.0	0.0	0.0	0.0	0.0	0.0	0.0	0.0	0.0	0.0	0.0	0.0
Mycosphaerellaceae	0.0	0.0	0.0	0.0	0.0	0.0	0.0	0.0	0.0	0.0	0.0	0.0	0.0	0.0	0.0
Myriangiaceae	0.0	0.0	0.0	0.0	0.0	0.0	0.0	0.0	0.0	0.0	0.0	0.0	0.0	0.0	0.0
Myxotrichaceae	0.0	0.1	0.1	0.0	0.0	0.0	0.1	0.1	0.1	0.0	0.0	0.1	0.6	0.8	0.0
Naohideales incertae sedis	0.0	0.0	0.0	0.0	0.0	0.0	0.0	0.0	0.0	0.0	0.0	0.0	0.0	0.0	0.0
Nectriaceae	0.1	0.1	0.2	0.0	0.0	0.0	0.3	0.2	0.1	0.0	0.0	0.0	0.0	0.0	0.1
Neocallimastigaceae	0.8	1.2	0.8	1.8	0.1	0.0	2.1	1.2	1.6	0.1	0.1	0.1	0.5	0.2	0.2
Niaceae	0.0	0.0	0.0	0.1	0.0	0.0	0.0	0.0	0.0	0.0	0.0	0.0	0.0	0.0	0.0
Nidulariaceae	0.3	0.9	1.4	0.6	0.0	0.1	0.3	0.2	0.0	0.0	0.0	0.1	0.0	0.0	0.0
Niessliaceae	0.1	0.0	0.0	0.0	0.0	0.0	0.3	0.1	0.0	0.0	0.0	0.0	0.1	0.8	0.4
Nitschkiaceae	0.0	0.0	0.0	0.0	0.0	0.0	0.0	0.0	0.0	0.0	0.0	0.0	0.0	0.1	0.0
Nucleariidae	0.2	0.3	0.0	0.3	0.0	0.0	1.9	0.2	0.5	0.0	0.1	0.0	0.2	0.3	0.0
Oedogoniomycetaceae	0.0	0.1	0.1	0.1	0.0	0.0	0.1	0.0	0.3	0.0	0.0	0.0	0.0	0.0	0.0
Olpidiaceae	0.0	0.4	0.1	0.1	0.0	0.0	0.1	0.1	0.1	0.0	0.0	0.0	0.1	0.2	0.1
Onygenaceae	0.0	0.0	0.0	0.0	0.0	0.0	0.0	0.0	0.0	0.0	0.0	0.0	0.0	0.0	0.0
Ophiocordycipitaceae	0.0	0.0	0.0	0.1	0.0	0.0	0.0	0.0	0.0	0.0	0.0	0.0	0.0	0.0	0.0
Ophioparmaceae	0.0	0.0	0.0	0.0	0.0	0.0	0.0	0.0	0.0	0.0	0.0	0.0	0.0	0.0	0.0
Pacisporaceae	0.0	0.0	0.0	0.1	0.0	0.0	0.0	0.0	0.0	0.0	0.0	0.0	0.0	0.0	0.0
Pannariaceae	0.0	0.0	0.0	0.0	0.0	0.0	0.0	0.0	0.0	0.0	0.0	0.0	0.0	0.0	0.0
Papulosaceae	0.0	0.0	0.0	0.0	0.0	0.0	0.7	0.6	0.2	0.0	0.0	0.0	1.8	0.3	1.0
Paraglomeraceae	0.0	0.0	0.0	0.0	0.0	0.0	0.0	0.0	0.0	0.1	0.1	0.0	0.0	0.0	0.4
Parmeliaceae	0.0	0.0	0.1	0.0	0.0	0.0	0.1	0.0	0.1	0.0	0.0	0.0	0.1	0.1	0.0
Patellariaceae	0.0	0.0	0.0	0.0	0.0	0.1	0.0	0.1	0.0	0.1	0.2	0.2	0.2	0.4	0.0

Paxillaceae	0.0	0.0	0.0	0.0	0.0	0.0	0.0	0.0	0.0	0.0	0.0	0.0	0.5	0.0	0.0
Pertusariaceae	0.0	0.0	0.0	0.0	0.0	0.0	0.0	0.0	0.0	0.0	0.0	0.0	0.0	0.0	0.0
Pezizaceae	0.0	0.0	0.0	0.7	0.0	0.0	0.0	3.0	0.0	0.6	0.1	1.1	0.0	0.0	0.0
Pezizales incertae sedis	0.0	0.0	0.0	0.0	0.0	0.0	0.1	0.0	0.0	0.0	0.0	0.0	0.0	0.0	0.0
Phaeosphaeriaceae	0.0	0.0	0.0	0.0	0.0	0.0	0.0	0.0	0.0	0.0	0.0	0.0	0.0	0.0	0.0
Phaeotrichaceae	0.0	0.0	0.0	0.0	0.0	0.0	0.2	0.1	0.0	0.0	0.0	0.0	0.0	0.0	0.0
Phaffomycetaceae	0.0	0.0	0.0	0.0	0.0	0.0	0.0	0.0	0.1	0.0	0.0	0.0	0.0	0.0	0.0
Phakopsoraceae	0.0	0.0	0.0	0.0	0.0	0.0	0.0	0.0	0.0	0.0	0.0	0.0	0.0	0.0	0.0
Phallaceae	0.1	0.0	0.0	0.0	0.0	0.0	0.0	0.3	0.1	0.0	0.0	0.0	0.0	0.0	0.1
Phanerochaetaceae	0.0	0.0	0.0	0.0	0.0	0.0	0.0	0.0	0.0	0.0	0.0	0.0	0.0	0.0	0.0
Phleogenaceae	0.0	0.0	0.0	0.0	0.0	0.0	0.0	0.0	0.0	0.0	0.0	0.0	0.0	0.0	0.0
Phlyctidaceae	0.0	0.0	0.0	0.0	0.0	0.0	0.3	0.0	0.0	0.0	0.0	0.0	0.0	0.0	0.0
Phragmidiaceae	0.0	0.0	0.0	0.0	0.0	0.0	0.0	0.0	0.0	0.0	0.0	0.0	0.0	0.0	0.0
Phyllachoraceae	0.0	0.0	0.0	0.0	0.0	0.0	0.0	0.0	0.0	0.0	0.0	0.0	0.0	0.0	0.0
Physalacriaceae	0.0	0.0	0.0	0.0	0.0	0.0	0.0	0.0	0.0	0.0	0.0	0.0	0.0	0.0	0.0
Physciaceae	0.0	0.0	0.0	0.0	0.0	0.0	0.0	0.0	0.0	0.0	0.0	0.0	0.0	0.1	0.0
Physodermataceae	0.0	0.0	0.2	0.1	0.1	0.0	0.1	0.1	0.1	0.0	0.1	0.3	0.0	0.1	0.0
Pileolariaceae	0.1	0.1	0.4	0.0	0.1	0.0	0.1	0.1	0.0	0.0	0.0	0.1	0.0	0.1	0.0
Piptocephalidaceae	0.4	0.5	3.1	0.4	0.1	0.1	0.4	0.3	0.5	0.0	0.1	0.2	0.2	0.4	0.1
Placynthiaceae	0.0	0.0	0.0	0.0	0.0	0.0	0.0	0.0	0.0	0.0	0.0	0.0	0.0	0.0	0.0
Platygloeaceae	0.0	0.1	0.0	0.1	0.0	0.0	0.0	0.0	0.0	0.0	0.0	0.0	0.0	0.0	0.0
Pleomassariaceae	0.0	0.0	0.0	0.0	0.0	0.0	0.0	0.0	0.0	0.0	0.0	0.0	0.0	0.0	0.0
Pleosporaceae	0.0	0.0	0.0	0.0	0.0	0.0	0.1	0.0	0.0	0.0	0.0	0.0	0.0	0.0	0.0
Pleosporales incertae sedis	0.0	0.1	0.0	0.0	0.1	0.0	0.0	0.0	0.0	0.0	0.0	0.0	0.0	0.0	0.0
Pleurotaceae	0.0	0.0	0.0	0.0	0.0	0.0	0.0	0.0	0.0	0.0	0.0	0.0	0.0	0.0	0.0
Pluteaceae	0.0	0.0	0.0	0.0	0.0	0.0	0.0	0.0	0.0	0.0	0.0	0.0	0.0	0.0	0.0
Pneumocystidaceae	0.0	0.1	0.0	0.0	0.0	0.0	0.0	0.0	0.0	0.0	0.0	0.0	0.1	0.1	0.0
Podaxaceae	0.0	0.0	0.0	0.0	0.0	0.0	0.0	0.0	0.0	0.0	0.0	0.0	0.3	0.0	0.0
Podoscyphaceae	0.0	0.1	0.2	0.0	0.0	0.0	0.1	0.3	0.0	0.0	0.1	0.0	0.0	0.0	0.1
Polyporaceae	0.0	0.0	0.0	0.0	0.0	0.0	0.8	0.2	0.0	0.0	0.1	0.1	0.0	0.0	0.9
Polyporales incertae sedis	0.0	0.0	0.1	0.0	0.0	0.0	0.1	0.3	0.0	0.0	0.0	0.0	0.0	0.0	0.1
Protomycetaceae	0.7	0.2	0.6	0.8	0.2	0.1	0.3	0.3	0.1	0.0	0.2	0.0	0.1	0.0	0.0
Psathyrellaceae	0.0	0.0	0.0	0.0	0.0	0.0	0.0	0.0	0.0	0.0	0.2	0.0	0.0	0.0	0.0
Psoraceae	0.0	0.0	0.0	0.0	0.0	0.0	0.0	0.0	0.0	0.0	0.0	0.0	0.0	0.0	0.0
Pterulaceae	0.0	0.0	0.0	0.0	0.0	0.0	0.0	0.0	0.0	0.0	0.0	0.0	0.0	0.0	0.0
Pucciniastraceae	0.0	0.0	0.0	0.0	0.0	0.0	0.3	0.0	0.3	0.0	0.1	0.0	0.0	0.0	0.0
Pyronemataceae	0.0	0.0	0.0	0.0	0.0	0.1	0.1	0.0	0.0	0.1	0.0	0.0	0.0	0.0	0.0
Pyxidiophoraceae	0.0	0.0	0.0	0.0	0.0	0.0	0.0	0.0	0.0	0.0	0.0	0.0	0.0	0.0	0.1
Ramalinaceae	0.0	0.0	0.0	0.0	0.0	0.0	0.0	0.0	0.0	0.0	0.0	0.0	0.0	0.0	0.0
Raveneliaceae	0.0	0.0	0.1	0.6	0.0	0.1	0.1	0.0	0.0	0.1	0.0	0.0	0.1	0.1	0.0
Rhizinaceae	0.0	0.0	0.0	0.0	0.0	0.0	0.0	0.0	0.0	0.0	0.0	0.0	0.0	0.0	0.0
Rhizocarpaceae	0.0	0.0	0.0	0.0	0.0	0.0	0.0	0.0	0.0	0.0	0.0	0.0	0.0	0.0	0.0
Rhizophydiaceae	0.0	0.0	0.0	0.0	0.0	0.0	0.0	0.0	0.0	0.0	0.0	0.0	0.0	0.0	0.0
Rhizopogonaceae	0.0	0.0	0.0	0.0	0.0	0.0	0.0	0.0	0.0	0.0	0.0	0.4	0.0	0.0	0.0
Rhynchostomataceae	0.0	0.0	0.0	0.0	0.0	0.0	0.0	0.0	0.0	0.0	0.0	0.0	0.0	0.0	0.0
Roccellaceae	0.0	0.0	0.0	0.0	0.0	0.0	0.0	0.0	0.0	0.0	0.0	0.0	0.0	0.0	0.0
Russulaceae	4.8	3.3	7.3	1.3	26.6	0.1	12.0	3.1	35.5	0.3	0.1	0.1	5.8	13.4	2.6
Rutstroemiaceae	0.0	0.0	0.0	0.0	0.0	0.0	0.0	0.0	0.0	0.0	0.0	0.0	0.0	0.0	0.0
Saccharomycetaceae	0.0	0.0	0.0	0.0	0.0	0.0	0.0	0.0	0.0	0.0	0.0	0.0	0.3	0.5	0.1
Saccharomycetales incertae sec	0.0	0.0	0.0	0.1	0.0	0.0	0.1	0.0	0.0	0.0	0.0	0.0	0.0	0.0	0.0
Saccharomycodaceae	0.0	0.0	0.0	0.0	0.0	0.0	0.0	0.0	0.0	0.0	0.0	0.0	0.0	0.0	0.0
Saccharomycopsidaceae	0.0	0.0	0.0	0.0	0.0	0.0	0.0	0.0	0.0	0.0	0.0	0.0	0.0	0.0	0.0
Saccoblastiaceae	0.0	0.0	0.0	0.0	0.0	0.0	0.0	0.0	0.0	0.0	0.0	0.0	0.0	0.0	0.0
Salpingoecidae	0.2	0.4	0.2	0.9	0.0	0.1	0.2	0.2	0.3	0.1	0.0	0.1	0.3	0.3	0.1
Sarcoscyphaceae	0.0	0.0	0.0	0.0	0.0	0.0	0.0	0.1	0.0	0.0	0.0	0.0	0.0	0.0	0.0
Sarcosomataceae	0.0	0.0	0.0	0.0	0.0	0.0	0.0	0.0	0.0	0.0	0.0	0.0	0.0	0.0	0.0
Schizophyllaceae	0.0	0.0	0.0	0.1	0.0	0.0	0.1	0.0	0.2	0.3	0.1	0.0	0.0	2.6	0.0
Schizoporaceae	0.0	0.0	0.0	0.0	0.0	0.0	0.0	0.0	0.0	0.0	0.0	0.0	2.5	1.7	0.4
Schizosaccharomycetaceae	0.0	0.0	0.0	0.2	0.0	0.0	0.1	0.0	0.0	0.0	0.0	0.0	0.0	0.0	0.0
Sclerodermataceae	0.0	0.0	0.0	0.0	0.0	0.0	2.6	0.1	0.8	0.0	0.0	0.0	0.0	0.0	0.0
Sclerotiniaceae	0.0	0.0	0.0	0.0	0.0	0.0	0.0	0.0	0.1	0.0	0.0	0.0	0.0	0.1	0.0
Scortechiniaceae	0.0	0.0	0.0	0.0	0.0	0.0	0.0	0.0	0.0	0.0	0.0	0.0	0.0	0.0	0.0
Sebacinaceae	0.4	1.0	2.5	14.5	0.0	1.3	3.2	0.1	0.0	0.5	0.8	0.2	0.0	0.0	0.0
Sebacinales incertae sedis	0.0	0.0	0.0	0.0	0.0	0.0	0.0	0.0	0.0	0.0	0.0	0.0	0.0	0.0	0.0
Septobasidiaceae	0.0	0.0	0.0	0.0	0.0	0.0	0.0	0.0	0.0	0.0	0.0	0.0	0.0	0.0	0.0
Serpulaceae	0.0	0.0	0.0	0.0	0.0	0.0	0.0	0.0	0.0	0.0	0.0	0.0	0.0	0.0	0.0
Sirobasidiaceae	0.0	0.0	0.0	0.2	0.0	0.0	0.2	0.1	0.1	0.0	0.1	0.0	0.0	0.0	0.0
Sordariaceae	0.0	0.0	0.0	0.0	0.0	0.0	0.1	0.1	0.0	0.0	0.0	0.0	0.0	0.0	0.0
Sordariales incertae sedis	0.0	0.0	0.0	0.0	0.0	0.0	0.0	0.0	0.0	0.0	0.0	0.0	0.0	0.0	0.0
Sordariomycetes incertae sedis	0.0	0.1	0.0	0.0	0.0	0.0	0.0	0.0	0.2	0.0	0.0	0.0	2.0	0.3	0.1
Sphaerobolaceae	0.0	0.0	0.0	0.0	0.0	0.0	0.0	0.0	0.0	0.0	0.0	0.0	0.0	0.0	0.0

Spiculogloeaceae	0.0	0.0	0.0	0.0	0.0	0.0	0.0	0.1	0.0	0.0	0.0	0.0	0.0	0.0	0.0
Spiculogloeales incertae sedis	0.0	0.0	0.0	0.0	0.0	0.0	0.0	0.0	0.0	0.0	0.0	0.0	0.0	0.0	0.0
Spizellomycetaceae	1.6	2.7	3.0	6.0	1.2	3.2	3.7	5.9	2.8	0.2	0.2	0.2	1.3	0.9	0.2
Spizellomycetales incertae sedi	0.0	0.0	0.0	0.2	0.0	0.0	0.1	0.0	0.2	0.0	0.1	0.0	0.0	0.0	0.0
Sporormiaceae	0.0	0.0	0.0	0.0	0.0	0.0	0.0	0.0	0.0	0.0	0.0	0.0	0.0	0.0	0.0
Stephanosporaceae	0.0	0.0	0.0	0.0	0.0	0.0	0.0	0.0	0.0	0.0	0.0	0.0	0.0	0.0	0.0
Stereaceae	0.0	0.0	0.0	0.0	0.0	0.0	0.0	0.0	0.0	0.0	0.0	0.0	0.0	0.0	1.3
Stictidaceae	0.0	0.0	0.0	0.0	0.0	0.0	0.1	0.0	0.0	0.0	0.0	0.0	0.0	0.0	0.0
Strophariaceae	0.0	0.0	0.0	0.0	0.0	0.0	0.0	0.0	0.0	0.0	0.0	0.0	0.0	0.0	0.0
Suillaceae	0.0	0.0	0.0	0.3	0.0	69.3	0.0	0.0	0.0	0.0	0.0	0.0	0.0	0.0	0.0
Teloschistaceae	0.0	0.0	0.0	0.0	0.0	0.0	0.0	0.0	0.0	0.0	0.0	0.0	0.0	0.0	0.0
Teratosphaeriaceae	0.0	0.0	0.0	0.0	0.0	0.0	0.0	0.0	0.0	0.0	0.0	0.0	0.0	0.0	0.0
Terramycetaceae	0.0	0.0	0.0	0.0	0.0	0.1	0.5	0.1	0.1	0.0	0.0	0.0	0.0	0.0	0.0
Testudinaceae	0.0	0.0	0.0	0.0	0.0	0.0	0.0	0.0	0.0	0.0	0.0	0.0	0.0	0.0	0.0
Thelephoraceae	61.9	33.6	31.6	0.0	0.0	0.6	9.5	33.8	1.3	1.1	0.3	0.8	5.9	0.3	0.8
Thyridiaceae	0.0	0.1	0.0	0.0	0.0	0.0	0.0	0.0	0.0	0.0	0.0	0.0	0.0	0.0	0.0
Tilletiaceae	0.0	0.0	0.0	0.0	0.0	0.0	0.0	0.0	0.0	0.0	0.0	0.0	0.0	0.0	0.0
Tremellaceae	0.9	0.9	4.4	1.0	0.3	0.4	1.4	0.5	0.2	0.1	0.0	0.2	0.1	0.2	0.1
Tremellales incertae sedis	0.0	0.0	0.0	0.0	0.0	0.0	0.0	0.0	0.0	0.0	0.0	0.0	0.0	0.0	0.0
Trichocomaceae	0.3	0.3	0.3	0.2	0.1	0.1	0.1	0.1	0.0	0.0	0.0	0.0	1.1	2.4	0.1
Tricholomataceae	3.2	12.2	0.8	1.2	0.1	0.4	12.2	21.5	10.1	1.7	2.2	2.2	4.1	16.3	68.6
Trichomonascaceae	9.3	3.3	13.7	2.3	1.4	1.6	3.2	1.9	0.5	0.3	0.6	2.1	3.1	6.5	0.5
Tuberaceae	0.0	0.0	0.0	0.0	0.0	0.0	0.0	0.0	0.0	0.0	0.0	0.0	0.0	0.0	0.0
Tulasnellaceae	0.0	0.0	0.0	0.0	0.0	0.0	0.0	0.1	0.2	0.0	0.0	0.0	0.0	0.0	0.0
Typhulaceae	0.0	0.0	0.0	0.0	0.0	0.0	0.0	0.0	0.0	0.0	0.0	0.1	0.0	0.0	0.0
Urocystaceae	0.0	0.0	0.2	0.0	0.0	0.0	0.0	0.0	0.0	0.0	0.0	0.0	0.0	0.0	0.0
Ustilaginaceae	0.0	0.0	0.0	0.0	0.0	0.0	0.0	0.0	0.0	0.0	0.0	0.0	0.0	0.0	0.0
Ustilentylomataceae	0.2	0.9	0.1	0.0	0.0	0.0	0.1	0.1	1.1	0.0	0.0	0.0	0.2	0.3	0.0
Valsaceae	0.0	0.0	0.0	0.0	0.0	0.0	0.1	0.0	0.0	0.0	0.0	0.0	0.0	0.0	0.0
Verrucariaceae	0.0	0.1	0.0	0.0	0.0	0.0	0.0	0.2	0.0	0.0	0.0	0.0	0.0	0.2	0.0
Vibrisseaceae	0.1	0.0	0.0	0.0	0.1	0.1	0.1	0.1	0.1	0.0	0.0	0.0	0.1	0.1	0.0
Volvocisporiaceae	0.0	0.0	0.0	0.0	0.0	0.0	0.0	0.0	0.0	0.0	0.0	0.0	0.0	0.0	0.0
Wickerhamomycetaceae	0.0	0.0	0.0	0.0	0.0	0.0	0.0	0.0	0.0	0.0	0.0	0.0	0.0	0.0	0.0
Xylariaceae	0.0	6.7	0.0	0.1	0.1	0.0	0.1	0.0	0.1	0.1	0.0	0.0	0.0	0.0	0.0
Xylariales incertae sedis	0.1	0.1	0.2	0.0	0.0	0.2	0.1	0.0	0.5	0.0	0.0	0.1	1.2	2.1	0.4

EF 2-5 cm 1	EF 2-5 cm 2	EF 2-5 cm 3	A 5-10 cm 1	A 5-10 cm 2	A 5-10 cm 3	EF 5-10 cm 1	EF 5-1- cm 2	EF 5-10 cm 3	forest floor	forest floor	forest floor	E 5-10 cm 1	E 5-10 cm 2	E 5-10 cm 3	F forest floor	F forest floor	F forest floor
0.0	0.0	0.0	0.1	0.0	0.0	0.0	0.0	0.0	0.0	0.1	0.0	0.0	0.1	0.0	2.0	0.0	0.1
0.1	0.0	0.0	0.0	0.1	0.0	0.1	0.0	0.0	0.0	0.0	0.0	0.0	0.0	0.0	0.0	0.0	0.3
0.2	0.0	0.0	0.0	1.9	0.8	0.0	0.0	0.0	0.1	0.1	0.1	0.0	0.0	0.0	0.0	0.0	0.0
0.0	0.0	0.0	0.1	0.0	0.0	0.0	0.0	0.0	0.0	0.0	0.0	0.0	0.1	0.0	0.0	0.1	0.1
0.2	0.0	0.0	0.0	0.0	0.0	0.0	0.0	0.0	0.0	0.0	0.0	0.0	0.0	0.0	0.0	0.0	0.0
0.0	0.1	0.1	0.7	0.0	0.0	0.0	0.0	0.0	0.0	0.0	0.0	0.0	0.1	0.0	0.1	0.0	0.1
0.0	0.0	0.0	0.0	0.0	0.0	0.0	0.0	0.0	0.0	0.0	0.0	0.0	0.0	0.0	0.0	0.0	0.0
0.1	0.0	0.2	0.0	0.1	61.2	0.6	0.2	0.0	46.8	83.1	79.8	0.1	1.9	0.0	2.5	0.0	0.0
0.1	0.5	0.1	0.2	0.1	0.2	0.1	0.3	0.1	0.0	0.1	0.1	0.1	0.3	0.1	1.5	0.1	0.3
0.0	0.1	0.0	0.0	0.1	0.1	0.0	0.0	0.1	0.1	0.0	0.0	0.0	0.0	0.0	0.3	0.1	0.0
0.0	0.0	0.0	0.0	0.0	0.0	0.1	0.1	0.1	0.1	0.0	0.0	0.0	0.0	0.0	0.0	0.0	0.0
0.5	0.0	0.0	0.0	0.1	0.0	0.0	0.0	0.0	0.0	0.0	0.0	0.0	0.0	0.0	0.1	0.0	0.2
0.0	0.0	0.0	0.0	0.0	0.0	0.0	0.0	0.0	0.0	0.0	0.0	0.0	0.0	0.0	0.0	0.0	0.0
0.0	0.0	0.0	0.0	0.0	0.0	0.0	0.0	0.0	0.0	0.3	0.1	0.0	0.0	0.0	0.0	0.0	0.0
1.4	0.6	0.0	0.7	1.6	0.9	1.6	0.2	0.9	0.0	0.0	0.0	0.4	1.8	0.0	0.0	0.0	0.0
0.0	0.0	0.0	0.0	0.0	0.0	0.0	0.0	0.0	0.0	0.0	0.0	0.0	0.0	0.0	0.0	0.0	0.0
0.0	0.0	0.0	0.0	0.0	0.0	0.0	0.0	0.0	0.0	0.0	0.0	0.0	0.0	0.0	0.0	0.0	0.0
0.0	0.0	0.0	0.0	0.0	0.0	0.0	0.0	0.0	0.0	0.0	0.0	0.0	0.0	0.0	0.0	0.0	0.0
0.0	0.0	0.0	0.0	0.0	0.0	0.0	0.0	0.0	0.0	0.0	0.0	0.0	0.0	0.0	0.0	0.0	0.1
0.0	0.0	0.0	0.0	0.0	0.0	0.0	0.0	0.0	0.0	0.0	0.0	0.0	0.0	0.0	0.0	0.0	0.0
0.7	0.4	0.1	0.3	0.2	0.3	0.8	0.3	0.8	0.7	0.1	0.4	0.0	0.2	0.1	5.7	3.0	4.4
0.0	0.0	0.0	0.0	0.0	0.0	0.0	0.0	0.0	0.0	0.0	0.0	0.0	0.0	0.0	0.0	0.0	0.0
0.0	0.1	0.0	0.3	0.0	0.0	0.0	0.1	0.0	0.0	0.0	0.0	0.0	0.1	0.0	0.1	0.0	0.3
0.4	0.7	0.1	0.0	0.5	0.3	0.5	0.1	0.3	1.3	0.1	0.6	0.1	0.2	0.1	0.8	0.8	0.8
0.0	0.0	0.0	0.0	0.0	0.0	0.0	0.0	0.0	0.0	0.0	0.0	0.0	0.0	0.0	0.1	0.0	0.3
0.0	0.0	0.0	0.0	0.0	0.0	0.0	0.0	0.0	0.0	0.0	0.0	0.0	0.0	0.0	0.0	1.6	3.1
0.0	0.0	0.0	0.0	0.0	0.0	0.0	0.0	0.0	0.0	0.0	0.0	0.0	0.0	0.0	0.0	0.0	0.0
0.0	0.0	0.0	0.0	0.1	0.0	0.0	0.0	0.0	0.0	0.0	0.0	0.0	0.0	0.0	0.1	0.0	5.1
0.6	0.5	0.2	0.2	0.2	0.1	0.5	0.6	0.1	0.0	0.0	0.0	0.0	0.0	0.0	0.0	0.0	0.0
0.0	0.0	0.0	0.0	0.0	1.1	0.0	0.0	0.0	0.0	0.0	0.0	0.0	0.0	0.0	0.0	0.0	0.0
0.0	0.0	0.0	0.0	0.0	0.0	0.0	0.0	0.0	0.0	0.0	0.0	0.0	0.0	0.0	0.0	0.0	0.0
0.3	0.1	0.1	0.3	0.2	0.0	0.3	0.5	0.2	0.1	0.0	0.0	0.2	0.4	0.0	0.9	0.1	0.1
0.3	0.7	0.4	0.8	0.4	0.0	0.3	0.3	0.6	0.4	0.1	0.2	0.2	0.4	0.1	0.3	0.1	0.5
0.0	0.0	0.0	0.0	0.0	0.0	0.0	0.0	1.3	0.0	0.0	0.0	0.3	0.0	0.7	0.0	0.0	0.0
1.0	0.2	0.5	0.1	0.0	0.0	0.0	0.0	0.0	4.5	0.8	0.0	0.0	0.0	0.0	0.0	0.0	0.7
0.0	0.0	0.0	0.0	0.0	0.0	0.0	0.0	0.0	0.3	0.0	0.1	0.0	0.0	0.0	0.1	0.1	0.0
0.4	0.0	0.1	0.0	0.0	0.1	0.1	0.0	0.0	0.0	0.0	0.0	0.0	0.0	0.0	0.1	0.0	0.1
0.0	0.0	0.0	0.2	0.4	0.0	0.1	0.0	0.0	0.0	0.0	0.0	0.0	0.0	0.0	0.0	0.0	0.0
0.0	0.0	0.0	0.0	0.0	0.0	0.0	0.0	0.0	0.0	0.0	0.0	0.0	0.0	0.0	0.0	0.0	0.0
0.0	0.0	0.0	0.0	0.1	0.0	0.0	0.0	0.0	0.0	0.0	0.0	0.0	0.0	0.0	0.0	0.0	0.0
0.0	0.0	0.0	0.0	0.0	0.0	0.0	0.0	0.0	0.0	0.0	0.0	0.0	0.0	0.0	0.0	0.0	0.0
0.0	0.0	0.0	0.0	0.0	0.0	0.0	0.0	0.0	0.0	0.0	0.0	0.0	0.1	0.0	0.1	0.0	0.0
0.0	0.0	0.0	0.0	0.0	0.0	0.0	0.0	0.0	0.0	0.0	0.0	0.0	0.0	0.0	0.0	0.0	0.0
0.0	0.1	0.0	0.1	0.0	0.0	0.0	0.0	0.0	0.0	0.0	0.0	0.0	0.0	0.0	0.4	0.0	0.1
0.0	0.1	0.0	0.1	0.0	0.0	0.0	0.0	0.0	0.0	0.0	0.0	0.0	0.0	0.0	0.4	0.0	0.1
0.2	0.1	0.1	0.1	0.1	0.0	0.1	0.1	0.2	0.1	0.0	0.1	0.1	0.1	0.0	0.0	0.1	0.0
0.5	1.7	0.5	0.5	0.5	0.1	2.3	0.8	1.5	0.0	0.0	0.0	0.8	0.8	0.3	0.4	0.0	0.3
0.0	0.0	0.0	0.0	0.0	0.0	0.0	0.0	0.0	0.0	0.0	0.0	0.0	0.0	0.0	0.0	0.0	0.0
0.3	0.5	0.0	0.2	0.0	0.0	0.4	0.0	0.3	0.0	0.0	0.0	0.0	0.0	0.0	0.2	0.1	0.2
0.0	0.0	0.0	0.0	0.0	0.0	0.0	0.0	0.0	0.0	0.0	0.0	0.0	0.0	0.0	0.0	0.0	0.0
0.1	0.0	0.0	0.1	0.0	0.0	0.0	0.0	0.0	0.0	0.0	0.0	0.0	0.0	0.0	0.2	0.0	0.0
0.0	0.1	0.0	0.0	0.0	0.0	0.0	0.0	0.0	0.0	0.0	0.0	0.1	0.0	0.0	0.0	0.0	0.0
0.1	0.2	0.1	0.1	0.0	0.0	0.1	0.2	0.0	0.0	0.0	0.0	0.0	0.1	0.1	0.0	0.0	0.2
0.1	0.0	0.0	0.2	1.2	0.1	0.0	0.1	0.1	0.2	0.1	0.4	0.1	0.1	0.0	0.8	0.2	0.6
0.1	0.0	0.0	0.0	0.1	0.0	0.0	0.0	0.0	0.2	0.0	0.2	0.0	0.0	0.0	1.2	0.8	0.4
0.0	0.0	0.0	0.0	0.0	0.0	0.0	0.0	0.0	0.0	0.0	0.0	0.0	0.0	0.0	0.1	0.1	0.1
0.0	0.0	0.0	0.0	0.0	0.0	0.0	0.0	0.0	0.0	0.0	0.0	0.0	0.0	0.0	0.0	0.0	0.0
1.6	1.0	0.2	0.4	0.4	0.1	0.5	0.3	3.4	0.0	0.0	0.0	1.5	0.6	0.1	0.1	0.0	0.0
2.1	1.3	0.6	1.9	1.1	0.3	1.1	1.2	1.2	0.2	0.1	0.2	0.2	0.5	0.4	1.3	0.2	1.3
0.0	0.1	0.0	0.9	0.1	0.0	0.0	0.2	0.0	0.0	0.0	0.0	0.0	0.0	0.0	0.3	0.0	0.5
0.0	0.0	0.1	0.0	0.0	0.0	0.0	0.1	0.0	0.0	0.0	0.0	0.0	0.0	0.0	0.0	0.0	0.0
0.0	0.0	0.0	0.0	0.0	0.0	0.0	0.0	0.0	0.0	0.0	0.0	0.0	0.0	0.0	0.0	0.0	0.0
0.3	0.4	0.1	0.1	7.4	1.4	0.8	0.2	0.1	0.0	0.0	0.0	0.0	0.1	0.0	0.0	1.0	0.5
0.5	0.4	0.3	0.5	0.1	0.0	0.4	0.2	0.3	0.0	0.0	0.0	0.1	0.4	0.0	0.8	0.5	0.3
0.0	0.0	0.0	0.0	0.1	0.0	0.0	0.0	0.0	0.4	0.0	0.8	0.0	0.2	0.0	1.7	0.4	0.9

0.0	0.0	0.0	0.0	0.0	0.0	0.0	0.0	0.0	0.0	0.0	0.0	0.0	0.0	0.0	0.0	0.0	0.0
0.0	0.0	0.0	0.0	0.0	0.0	0.0	0.0	0.0	0.0	0.0	0.0	0.0	0.0	0.0	0.0	0.0	0.0
1.3	0.6	0.3	4.5	0.2	0.0	1.0	0.5	0.4	0.0	0.0	0.0	2.3	3.7	0.2	0.0	0.0	0.0
0.3	0.3	0.2	0.6	0.7	0.2	0.3	0.7	0.5	0.1	0.0	0.2	0.7	0.4	0.2	0.9	0.2	0.3
0.0	0.0	0.0	0.0	0.0	0.0	0.0	0.0	0.0	0.0	0.0	0.0	0.0	0.0	0.0	0.0	0.0	0.0
0.1	0.0	0.0	0.1	0.1	0.0	0.1	0.1	0.0	0.0	0.0	0.0	0.0	0.0	0.0	0.0	0.0	0.0
0.4	0.5	0.2	0.0	0.1	0.0	0.8	0.2	0.4	0.0	0.0	0.0	0.1	0.2	0.0	0.0	0.0	0.0
0.0	0.0	0.0	0.0	0.0	0.0	0.0	0.0	0.0	0.0	0.0	0.0	0.0	0.0	0.0	0.0	0.0	0.0
0.1	0.0	0.1	0.1	0.0	0.0	0.0	0.0	0.1	0.0	0.0	0.0	0.0	0.0	0.0	0.1	0.0	0.5
0.6	0.0	0.5	0.4	0.0	1.5	0.0	0.4	0.0	0.1	0.0	0.1	0.0	0.0	0.0	0.1	0.4	1.2
0.0	0.0	0.0	0.1	0.1	0.1	0.0	0.0	0.2	0.0	0.0	0.0	0.0	0.2	0.0	0.1	0.0	0.0
0.1	0.0	0.0	8.3	6.1	4.1	0.0	0.0	0.1	0.6	0.0	0.2	2.1	0.2	16.4	1.7	0.3	11.9
0.0	0.0	0.1	0.0	0.0	0.0	0.0	0.0	0.0	0.0	0.0	0.0	0.0	0.0	0.0	0.0	0.0	0.0
0.1	0.0	0.0	0.0	0.0	0.0	0.0	0.0	0.2	0.0	0.0	0.0	0.0	0.0	0.0	0.0	0.1	0.3
0.0	0.0	0.0	0.0	0.0	0.0	0.0	0.0	0.0	0.0	0.0	0.0	0.0	0.0	0.0	0.0	0.0	0.0
0.0	0.0	0.0	0.0	0.0	0.0	0.0	0.0	0.0	0.0	0.0	0.0	0.0	0.0	0.0	0.0	0.0	0.1
0.2	0.2	0.1	0.2	0.1	0.0	0.1	0.0	0.2	0.0	0.0	0.0	0.1	0.1	0.0	0.0	0.0	0.1
0.1	0.0	0.0	0.0	0.0	0.0	0.0	0.0	0.0	0.0	0.0	0.0	0.0	0.0	0.0	0.0	0.0	0.0
0.0	0.0	0.0	0.0	0.0	0.0	0.0	0.0	0.0	0.0	0.0	0.0	0.0	0.0	0.0	0.5	0.0	0.0
0.0	0.0	0.0	0.0	0.0	0.0	0.0	0.0	0.0	0.0	0.0	0.0	0.0	0.0	0.0	0.1	0.1	0.0
0.0	0.0	0.0	0.0	0.0	0.0	0.0	0.0	0.0	0.0	0.0	0.0	0.0	0.0	0.0	0.0	0.0	0.0
0.0	0.0	0.0	0.0	0.0	0.0	0.0	0.0	0.0	0.0	0.0	0.0	0.0	0.0	0.0	0.0	0.0	0.0
0.0	0.0	0.0	0.0	0.0	0.0	0.0	0.0	0.0	0.0	0.0	0.0	0.0	0.0	0.0	0.0	0.0	0.0
0.0	0.0	0.0	0.0	0.0	0.0	0.0	0.0	0.0	0.0	0.0	0.0	0.0	0.0	0.0	0.0	0.0	0.0
0.0	0.0	0.0	0.0	0.0	0.0	0.0	0.0	0.0	0.0	0.0	0.0	0.0	0.0	0.0	0.0	0.0	0.0
0.2	0.1	0.1	0.1	0.2	0.1	0.2	0.0	0.5	0.0	0.0	0.1	0.2	0.1	0.1	1.1	0.1	0.1
0.7	1.1	0.6	0.5	1.1	0.4	0.9	0.7	0.8	0.4	0.1	0.5	0.5	0.9	0.8	2.6	0.4	1.5
0.0	0.0	0.0	0.0	0.0	0.0	0.0	0.0	0.0	0.0	0.0	0.0	0.0	0.0	0.0	0.0	0.0	0.0
0.1	0.0	0.0	0.0	0.0	0.0	0.1	0.0	0.0	0.0	0.0	0.0	0.0	0.0	0.0	0.0	0.0	0.0
0.0	0.0	0.0	0.0	0.0	0.0	0.0	0.0	0.0	0.0	0.0	0.0	0.0	0.0	0.0	0.0	0.0	0.0
0.0	0.0	0.0	0.0	0.0	0.0	0.0	0.0	0.0	0.0	0.0	0.0	0.0	0.0	0.0	0.0	0.0	0.0
0.0	0.0	0.0	0.0	0.0	0.0	0.0	0.0	0.0	0.0	0.0	0.0	0.0	0.0	0.0	0.0	0.0	0.0
0.2	0.1	0.1	0.2	0.4	0.1	0.1	0.6	0.1	0.1	0.1	0.5	0.7	0.4	0.0	0.3	0.3	0.5
0.0	0.0	0.0	0.0	0.0	0.0	0.1	0.0	0.0	0.0	0.0	0.0	0.0	0.0	0.0	0.0	0.0	0.0
0.0	0.0	0.0	0.0	0.0	0.0	0.0	0.0	0.0	0.0	0.0	0.0	0.0	0.0	0.0	0.0	0.0	0.0
0.2	0.3	0.0	0.0	0.2	0.0	0.1	0.0	0.0	0.1	0.0	0.0	0.0	0.2	0.0	0.0	0.0	0.0
0.0	0.0	0.0	0.0	0.0	0.0	0.0	0.0	0.0	0.0	0.0	0.0	0.0	0.0	0.0	0.0	0.0	0.0
0.0	0.0	0.0	0.4	0.0	0.0	0.0	0.0	0.0	0.0	0.0	0.0	0.0	0.0	0.0	1.7	0.0	0.6
0.1	0.1	0.0	2.5	0.2	0.1	0.1	0.1	0.1	0.0	0.0	0.0	0.0	0.0	0.0	0.5	0.0	0.0
0.0	0.0	0.0	0.0	0.0	0.0	0.0	0.0	0.0	0.0	0.0	0.0	0.0	0.0	0.0	0.0	0.0	0.0
0.0	0.0	0.0	0.0	0.1	0.0	0.0	0.0	0.0	0.0	0.0	0.0	0.0	0.0	0.0	0.0	0.0	0.0
1.2	3.3	0.4	1.5	0.5	0.3	1.0	1.0	0.6	0.2	0.0	0.4	0.5	0.9	0.4	2.1	0.3	0.7
0.0	0.0	0.0	0.2	0.0	0.0	0.0	0.0	0.0	0.0	0.0	0.0	0.0	0.0	0.0	0.0	0.0	0.0
0.0	0.0	0.2	0.1	0.1	0.1	0.0	1.7	0.0	2.0	3.1	5.2	0.0	0.0	0.0	0.0	0.0	0.0
0.3	0.2	0.1	1.9	2.9	1.0	0.4	0.2	0.2	0.0	0.0	0.0	0.0	0.0	0.0	0.0	0.0	0.1
0.0	0.0	0.1	0.1	0.1	0.1	0.0	0.0	0.0	0.1	0.0	0.0	0.0	0.1	0.1	1.2	0.2	0.9
0.0	0.0	0.0	0.0	0.0	0.0	0.0	0.0	0.0	0.0	0.0	0.0	0.0	0.0	0.0	0.0	0.0	0.0
0.0	0.0	0.0	0.0	0.0	0.0	0.0	0.0	0.0	0.0	0.0	0.0	0.0	0.0	0.0	0.0	0.0	0.0
0.0	0.0	0.0	0.0	0.0	0.0	0.0	0.0	0.0	0.0	0.0	0.0	0.0	0.0	0.0	0.0	0.0	0.0
0.1	0.0	0.0	0.7	0.0	0.1	0.1	0.0	0.0	0.1	0.1	0.0	0.0	0.1	0.0	1.0	0.1	0.1
0.1	0.0	0.8	0.5	0.3	0.0	0.0	2.4	0.0	0.4	0.1	0.0	0.0	0.1	0.0	0.0	0.2	0.1
0.0	0.0	0.0	0.0	0.0	0.0	0.0	0.0	0.0	0.1	0.1	0.0	0.0	0.0	0.0	0.0	0.0	0.0
1.0	0.4	0.1	0.5	0.4	0.0	0.3	0.3	0.6	0.0	0.0	0.0	0.1	0.0	0.0	0.1	0.0	0.2
0.0	0.0	0.0	0.0	0.0	0.0	0.0	0.0	0.0	0.0	0.0	0.0	0.0	0.0	0.0	0.0	0.0	0.0
0.0	0.0	0.4	0.0	0.0	0.0	0.0	0.0	0.0	0.0	0.0	0.0	0.0	0.0	0.0	0.4	7.3	0.0
0.0	0.0	0.0	0.0	0.0	0.0	0.0	0.0	0.0	0.0	0.0	0.0	0.1	0.1	0.1	0.0	6.0	0.1
0.0	0.0	0.0	0.0	0.2	0.0	0.0	0.0	0.0	0.0	0.0	0.0	0.0	0.0	0.0	0.0	0.0	0.0
0.0	0.1	0.1	0.1	0.1	0.0	0.1	0.1	0.1	0.0	0.0	0.0	0.1	0.1	0.0	0.5	0.0	0.0
0.1	0.1	0.1	0.1	0.1	0.0	0.1	0.1	0.1	0.1	0.0	0.0	0.0	0.3	0.0	0.7	0.0	0.3
0.0	0.0	0.0	0.0	0.0	0.0	0.0	0.0	0.0	0.0	0.0	0.0	0.0	0.0	0.0	0.0	0.0	0.0
0.2	0.1	0.1	0.2	0.0	0.0	0.4	0.2	0.1	0.0	0.0	0.1	0.2	1.0	0.1	0.0	0.0	0.1
0.0	0.0	0.0	0.0	0.0	0.1	0.0	0.0	0.0	0.0	0.0	0.0	0.1	0.0	0.4	0.0	0.0	0.0
0.0	0.0	0.0	0.0	0.0	0.0	0.0	0.0	0.0	0.0	0.0	0.0	0.0	0.0	0.0	0.0	0.0	0.0
0.0	0.0	0.0	0.0	0.0	0.0	0.0	0.0	0.0	0.0	0.0	0.0	0.0	0.0	0.0	0.0	0.0	0.0
0.1	0.5	0.1	0.2	0.3	0.1	0.5	0.3	0.2	0.1	0.0	0.1	0.2	0.1	0.1	0.2	0.0	0.1
0.3	0.0	0.0	0.1	0.1	0.1	0.1	0.3	0.1	0.0	0.0	0.0	0.0	0.1	0.0	0.1	0.1	0.3
0.5	0.7	0.2	0.8	0.4	0.3	0.8	0.5	1.1	0.3	0.0	0.3	0.3	0.7	0.2	5.2	5.6	2.8
0.0	0.0	0.0	0.0	0.1	0.0	0.0	0.0	0.0	0.0	0.0	0.0	0.0	0.0	0.0	0.0	0.0	0.0
0.0	0.0	1.2	0.1	0.0	0.0	0.0	1.3	0.0	0.0	0.0	0.0	0.0	0.0	0.0	0.0	0.3	0.1
0.0	0.0	0.1	0.1	0.0	0.0	0.0	0.0	0.0	0.1	0.0	0.0	0.0	0.0	0.0	0.3	0.2	0.6
1.9	1.0	1.3	1.3	2.3	1.7	1.1	1.3	1.6	0.2	0.0	0.5	0.4	0.8	0.1	1.3	0.4	2.6

0.0	0.0	0.0	0.0	0.0	0.0	0.0	0.0	0.0	0.0	0.0	0.0	0.0	0.0	0.0	0.0	0.0	0.0
0.1	0.2	0.0	1.8	0.1	0.0	0.0	0.0	0.0	0.1	0.0	0.0	0.1	0.0	0.0	3.4	1.6	19.2
0.0	0.0	0.0	0.1	0.4	0.0	0.0	0.1	0.1	0.0	0.0	0.0	0.1	0.3	0.1	0.2	0.0	0.1
0.0	0.0	0.0	0.0	1.5	0.8	0.0	0.0	0.4	0.0	0.0	0.0	0.0	0.0	0.0	0.0	0.0	0.0
0.0	0.0	0.0	0.0	0.0	0.0	0.0	0.0	0.0	0.2	0.0	0.0	0.0	0.0	0.0	0.0	0.0	0.0
0.0	0.0	0.0	0.0	0.0	0.0	0.0	0.0	0.0	0.0	0.0	0.0	0.0	0.0	0.2	0.0	0.0	0.0
0.0	0.0	0.0	0.0	0.0	0.0	0.0	0.0	0.0	0.0	0.0	0.0	0.0	0.0	0.0	0.0	1.2	0.0
0.0	0.0	0.0	0.0	0.0	0.0	0.0	0.0	0.0	0.0	0.0	0.0	0.0	0.0	0.0	0.0	0.0	0.0
0.0	0.0	0.0	0.0	0.0	0.0	0.0	0.0	0.0	0.0	0.0	0.0	0.0	0.0	0.0	0.0	0.0	0.1
3.7	2.3	0.5	0.1	0.4	0.1	0.8	2.1	0.5	0.7	0.1	0.6	0.1	0.4	0.1	0.8	0.2	0.5
0.0	0.0	0.0	0.0	0.0	0.0	0.2	0.1	0.0	0.0	0.0	0.0	0.0	0.0	0.0	0.0	0.0	0.0
0.0	0.0	0.0	0.0	0.0	0.0	0.0	0.0	0.0	0.0	0.0	0.0	0.0	0.0	0.0	0.0	0.0	0.0
0.0	0.0	0.0	0.0	0.2	0.0	0.0	0.0	0.0	0.0	0.0	0.0	17.1	0.0	27.3	0.0	0.0	0.3
0.0	0.0	0.0	0.0	0.1	0.0	0.0	0.0	0.0	0.0	0.0	0.0	0.0	0.0	0.0	0.2	0.0	0.0
0.1	0.0	0.0	0.0	0.0	0.0	0.0	0.1	0.0	0.0	0.0	0.0	0.0	0.0	0.0	0.0	0.0	0.0
0.4	0.1	0.1	0.1	0.2	0.0	0.6	0.1	0.1	0.0	0.0	0.0	0.1	0.0	0.1	0.0	0.0	0.1
0.0	0.0	0.1	0.0	0.1	0.0	0.0	0.0	0.0	0.0	0.0	0.0	0.0	0.0	0.0	0.0	0.3	0.0
0.1	0.0	0.1	0.0	0.0	0.0	0.1	0.1	0.0	0.0	0.0	0.0	0.0	0.1	0.0	0.1	0.0	0.1
0.0	0.0	0.0	0.1	0.0	0.0	0.0	0.0	0.0	0.1	0.0	0.0	0.0	0.0	0.0	0.0	0.0	0.0
0.0	0.0	0.0	0.0	0.0	0.0	0.0	0.0	0.0	0.0	0.0	0.0	0.0	0.0	0.0	0.0	0.0	0.0
0.0	0.0	0.0	0.0	0.0	0.0	0.0	0.1	0.0	0.0	0.0	0.0	0.1	0.0	0.0	0.0	0.0	0.0
0.0	0.0	0.0	0.0	0.0	0.0	0.0	0.0	0.0	0.0	0.0	0.0	0.0	0.0	0.0	0.2	0.0	0.0
0.2	0.1	0.1	0.0	0.1	0.1	0.1	0.3	0.4	0.0	0.0	0.0	0.2	0.0	0.0	0.2	0.1	0.0
0.0	0.0	0.0	0.0	0.0	0.0	0.0	0.0	0.0	0.0	0.0	0.0	0.0	0.0	0.0	0.0	0.0	0.0
0.0	0.0	0.0	0.0	0.0	0.0	0.0	0.0	0.0	0.0	0.0	0.0	0.0	0.0	0.0	0.0	0.0	0.0
0.0	0.0	0.0	0.0	0.0	0.0	0.0	0.0	0.0	0.0	0.0	0.0	0.0	0.0	0.0	0.0	0.0	0.0
0.1	0.0	0.0	0.0	0.0	0.0	0.0	0.0	0.1	0.0	0.0	0.0	0.1	0.1	0.0	0.0	0.0	0.0
0.6	0.3	0.1	0.1	0.1	0.0	0.3	0.2	0.4	0.0	0.0	0.0	1.9	3.0	0.7	0.2	0.0	0.1
0.0	0.0	0.1	0.0	0.0	0.0	0.0	0.1	0.0	0.0	0.0	0.0	0.0	0.0	0.0	0.0	0.0	0.0
0.0	0.0	0.0	0.0	0.0	0.0	0.0	0.0	0.0	0.0	0.0	0.0	0.0	0.0	0.0	0.0	0.0	0.0
0.0	0.0	0.0	0.0	0.0	0.0	0.0	0.0	0.0	0.0	0.0	0.0	0.0	0.0	0.0	0.0	0.0	0.0
0.0	0.0	0.1	0.0	0.0	0.0	0.0	0.0	0.0	0.0	0.0	0.1	0.0	0.0	0.0	0.6	0.1	0.3
0.0	0.0	0.0	0.0	0.0	0.0	0.0	0.0	0.0	0.0	0.0	0.0	0.0	0.0	0.0	0.0	0.0	0.0
0.1	0.5	0.1	0.0	0.0	0.0	0.6	0.0	0.0	0.0	0.0	0.0	0.0	0.0	0.0	0.0	0.0	0.0
0.1	0.0	0.2	0.0	0.0	0.1	0.0	0.0	0.0	0.0	0.0	0.0	0.1	0.1	0.0	0.0	0.0	0.0
0.1	0.0	0.0	0.0	0.0	0.0	0.1	0.0	0.0	0.0	0.0	0.0	0.0	0.0	0.0	0.1	0.0	0.0
0.0	0.2	0.1	0.0	0.0	0.0	0.1	0.0	0.1	0.0	0.0	0.0	0.0	0.1	0.0	0.1	0.0	0.0
0.0	0.0	0.0	0.0	0.0	0.0	0.0	0.0	0.0	0.0	0.0	0.0	0.0	0.0	0.0	0.0	0.0	0.0
0.0	0.0	0.0	0.0	0.0	0.0	0.0	0.0	0.0	0.0	0.0	0.0	0.0	0.0	0.0	0.0	0.0	0.0
0.0	0.0	0.0	0.0	0.1	0.0	0.1	0.0	0.0	0.0	0.0	0.0	0.0	0.1	0.0	0.0	0.0	0.0
0.0	0.0	0.0	0.0	0.0	0.0	0.0	0.0	0.0	0.0	0.0	0.0	0.0	0.0	0.0	0.0	0.0	0.0
0.0	0.0	0.0	0.0	0.0	0.0	0.0	0.0	0.0	0.0	0.0	0.0	0.0	0.0	0.0	0.0	0.0	0.0
1.0	3.4	0.9	0.2	2.2	0.6	1.8	0.6	1.7	0.0	0.0	0.0	0.2	2.3	0.1	0.1	0.0	0.1
0.0	0.0	0.0	0.0	0.0	0.0	0.0	0.0	0.0	0.0	0.0	0.0	0.0	0.0	0.0	0.0	0.0	0.0
0.0	0.0	0.0	0.0	0.0	0.0	0.0	0.1	0.0	0.1	0.0	0.0	0.0	0.0	0.0	0.2	0.1	0.1
3.2	10.0	1.7	0.8	4.4	0.7	9.4	2.2	4.9	0.0	0.0	0.0	1.8	2.7	0.8	0.0	0.0	0.0
0.0	0.0	0.0	0.0	0.0	0.0	0.0	0.0	0.0	0.0	0.0	0.0	0.0	0.0	0.0	0.0	0.0	0.0
0.0	0.0	0.0	0.0	0.0	0.0	0.0	0.0	0.0	0.0	0.0	0.0	0.0	0.0	0.0	0.0	0.0	0.0
0.0	0.0	0.0	0.0	0.0	0.0	0.0	0.0	0.0	0.0	0.0	0.0	0.0	0.1	0.0	0.0	0.0	0.0
0.0	0.0	0.0	0.0	0.0	0.0	0.0	0.0	0.0	0.0	0.0	0.0	0.0	0.0	0.0	0.0	0.0	0.0
0.0	0.0	0.0	0.0	0.0	0.0	0.0	0.0	0.0	0.0	0.0	0.0	0.0	0.0	0.0	0.0	0.0	0.0
0.1	0.1	0.0	0.1	0.0	0.1	0.1	0.1	0.0	0.0	0.0	0.1	0.0	0.1	0.0	0.0	0.1	0.1
0.0	0.0	0.0	0.0	0.0	0.0	0.0	0.0	0.0	0.0	0.0	0.0	0.0	0.0	0.0	0.0	0.0	0.0
0.4	0.4	0.3	0.3	0.1	0.0	0.2	0.9	0.2	0.0	0.0	0.0	0.3	0.2	0.1	0.2	0.1	0.1
2.2	1.9	0.4	2.6	1.3	0.0	2.4	0.9	1.0	0.2	0.0	0.1	0.7	2.2	0.5	0.5	0.0	0.5
0.0	0.0	0.0	0.0	0.0	0.0	0.0	0.0	0.0	0.0	0.0	0.0	0.0	0.0	0.0	0.0	0.0	0.0
2.5	1.7	0.4	1.5	0.6	0.4	6.9	0.5	1.2	0.0	0.0	0.0	0.5	1.2	0.3	0.1	0.0	0.0
0.2	0.1	0.1	0.0	0.1	0.0	0.1	0.0	0.0	0.0	0.0	0.1	0.0	0.0	0.0	0.3	0.1	0.3
0.0	0.0	0.0	0.0	0.0	0.0	0.0	0.0	0.0	0.1	0.0	0.0	0.0	0.0	0.0	0.4	0.0	0.0
0.3	0.1	0.0	0.5	0.2	0.0	0.1	0.2	0.0	0.0	0.0	0.0	0.1	0.1	0.1	0.8	0.2	0.4
0.0	0.0	0.1	0.4	0.0	0.0	0.0	0.0	0.2	0.0	0.0	0.0	0.0	0.0	0.0	0.1	0.0	0.0
0.2	0.1	0.1	0.2	0.1	0.1	0.2	0.0	0.0	0.1	0.0	0.0	0.1	0.2	0.1	0.2	0.1	0.2
0.0	0.0	0.0	0.0	0.0	0.0	0.0	0.0	0.0	0.0	0.0	0.0	0.0	0.0	0.0	0.0	0.0	0.0
0.0	0.0	0.0	0.0	0.0	0.0	0.0	0.0	0.0	0.0	0.0	0.0	0.0	0.0	0.0	0.0	0.0	0.0
0.0	0.0	0.0	0.0	0.1	0.0	0.0	0.0	0.0	0.0	0.0	0.0	0.1	0.0	0.0	0.1	0.0	0.0
0.0	0.0	0.0	0.0	0.1	0.0	0.0	0.0	0.1	0.0	0.0	0.0	0.0	0.0	0.0	0.1	0.0	0.0
0.0	0.0	0.0	0.0	0.0	0.0	0.0	0.0	0.0	0.0	0.0	0.0	0.0	0.0	0.0	0.0	0.0	0.0
0.1	0.1	0.0	0.1	0.0	0.0	0.0	0.0	0.0	0.0	0.0	0.0	0.1	0.0	0.0	0.4	0.1	0.1
0.0	0.0	0.0	0.0	0.0	0.0	0.0	0.0	0.0	0.4	0.9	1.5	0.0	0.0	0.0	0.0	0.0	0.1
0.0	0.2	0.0	0.0	0.1	0.0	0.0	0.0	0.0	0.1	0.0	0.0	0.0	0.0	0.0	0.0	0.0	0.0
0.0	0.0	0.6	0.0	0.2	0.2	0.0	0.0	0.8	0.1	0.0	0.0	0.0	0.2	0.0	0.0	0.0	0.0

0.0	0.0	0.0	0.0	0.0	0.0	0.0	0.0	0.0	0.1	0.0	0.0	0.0	0.0	0.0	0.0	0.0	0.0
0.0	0.0	0.0	0.0	0.0	0.0	0.0	0.0	0.0	0.0	0.0	0.0	0.0	0.0	0.0	0.0	0.0	0.0
0.7	0.0	0.0	0.1	2.5	3.7	0.1	0.0	0.4	0.0	0.0	0.0	0.0	0.1	0.2	0.0	0.0	0.0
0.2	0.0	0.0	0.1	0.0	0.0	0.1	0.1	0.0	0.0	0.0	0.0	0.0	0.0	0.0	0.0	0.0	0.0
0.0	0.0	0.0	0.0	0.0	0.0	0.0	0.0	0.0	0.0	0.0	0.0	0.0	0.0	0.0	0.0	0.0	0.0
0.2	0.0	0.0	0.2	0.2	0.0	0.2	0.0	0.0	0.0	0.0	0.0	0.0	0.1	0.0	0.0	0.0	0.0
0.0	0.0	0.0	0.0	0.0	0.0	0.0	0.0	0.0	0.0	0.0	0.0	0.0	0.0	0.0	0.0	0.0	0.0
0.0	0.0	0.0	0.0	0.0	0.0	0.0	0.0	0.0	0.0	0.0	0.0	0.0	0.0	0.0	0.0	0.0	0.0
0.0	0.0	0.1	0.0	0.0	0.0	0.0	0.1	0.0	0.0	0.0	0.0	0.0	0.0	0.0	0.0	0.0	0.1
0.0	0.0	0.0	0.0	0.0	0.0	0.0	0.0	0.0	0.0	0.0	0.0	0.0	0.0	0.0	0.0	0.0	0.0
0.0	0.0	0.0	0.0	0.0	0.0	0.0	0.0	0.0	0.0	0.0	0.0	0.0	0.0	0.0	0.0	0.0	0.0
0.0	0.0	0.0	0.0	0.0	0.0	0.0	0.0	0.0	0.0	0.0	0.0	0.0	0.0	0.0	1.2	0.0	0.0
0.0	0.0	0.0	0.0	0.0	0.0	0.0	0.0	0.0	0.0	0.0	0.0	0.0	0.0	0.0	0.1	0.0	0.0
0.0	0.0	0.0	0.0	0.0	0.0	0.0	0.0	0.0	0.0	0.0	0.0	0.0	0.0	0.0	0.0	0.0	0.0
0.0	0.0	0.0	0.0	0.0	0.0	0.0	0.0	0.0	0.0	0.0	0.0	0.0	0.0	0.0	0.0	0.0	0.0
0.0	0.1	0.0	0.0	0.0	0.0	0.0	0.0	0.0	0.0	0.0	0.0	0.0	0.0	0.0	0.0	0.0	0.0
0.0	0.4	0.1	0.0	0.3	0.0	0.5	0.2	0.3	0.0	0.0	0.1	0.0	0.2	0.0	0.2	0.0	0.0
0.2	0.1	0.0	0.0	0.0	0.0	0.3	0.1	0.4	0.0	0.0	0.0	0.2	0.3	0.1	0.0	0.0	0.0
3.8	3.9	2.1	0.5	2.2	0.7	6.5	0.8	5.9	0.0	0.0	0.0	1.1	1.2	0.4	0.2	0.1	0.1
0.0	0.0	0.0	0.0	0.0	0.0	0.0	0.0	0.0	0.0	0.0	0.0	0.0	0.0	0.0	0.0	0.0	0.0
0.2	0.0	0.0	0.1	0.0	0.0	0.0	0.0	0.0	0.0	0.0	0.0	0.0	0.0	0.0	0.0	0.0	0.0
0.0	0.0	0.0	0.0	0.0	0.0	0.0	0.0	0.0	0.0	0.0	0.0	0.0	0.0	0.0	0.0	0.0	0.0
0.0	0.0	0.0	0.0	0.0	0.0	0.1	0.1	0.0	0.0	0.0	0.0	0.0	0.0	0.0	0.0	0.0	0.0
0.1	0.1	0.0	0.0	0.2	0.0	0.1	0.0	0.0	0.0	0.0	0.0	0.0	0.0	0.0	0.0	0.0	0.1
0.0	0.0	0.0	0.0	0.0	0.0	0.0	0.0	0.0	0.0	0.0	0.0	0.0	0.0	0.0	0.0	0.0	0.0
0.0	0.0	0.0	0.0	0.0	0.0	0.0	0.0	0.0	0.0	0.0	0.0	0.0	0.0	0.0	0.0	0.3	0.0
0.0	0.0	0.0	0.0	0.0	0.0	0.0	0.0	0.0	0.0	0.0	0.0	0.0	0.0	0.0	0.0	0.0	0.0
0.0	0.0	0.0	0.0	0.0	0.1	0.0	0.0	0.0	0.0	0.0	0.0	0.0	0.0	0.0	0.0	0.0	0.0
0.0	0.2	0.0	0.5	0.3	0.3	0.0	0.0	0.0	0.1	0.0	0.0	0.0	0.0	0.0	0.1	0.0	0.0
0.0	0.0	0.7	0.3	0.4	0.0	0.0	0.3	0.0	0.6	0.2	0.2	0.0	0.0	0.0	1.2	0.0	0.3
0.0	0.0	0.0	0.0	0.0	0.0	0.0	0.0	0.0	0.0	0.0	0.0	0.0	0.0	0.0	0.3	0.0	0.7
0.6	2.0	1.1	0.2	0.2	0.0	1.6	0.5	1.1	0.0	0.0	0.1	1.2	1.4	0.3	0.1	0.0	0.0
0.0	1.6	0.0	0.7	0.1	0.0	0.1	0.0	0.0	0.0	0.0	0.0	0.0	0.0	0.0	6.3	0.0	0.0
0.0	0.0	0.0	0.0	0.0	0.0	0.0	0.0	0.0	0.1	0.0	0.0	0.0	0.0	0.0	0.0	0.0	0.1
0.0	0.0	0.0	0.0	0.0	0.0	0.0	0.0	0.0	0.0	0.0	0.0	0.0	0.0	0.0	0.1	0.0	0.0
0.0	0.0	0.0	0.2	0.0	0.0	0.0	0.0	0.0	0.0	0.0	0.0	0.0	0.0	0.0	0.5	0.2	0.3
0.1	0.0	0.0	0.4	0.1	0.1	0.0	0.0	0.0	0.0	0.0	0.0	0.6	0.0	0.2	0.0	0.0	0.1
0.0	0.0	0.0	0.0	0.0	0.0	0.0	0.0	0.0	0.0	0.0	0.0	0.0	0.0	0.0	0.4	0.2	0.0
0.0	0.0	0.0	0.0	0.0	0.0	0.0	0.0	0.0	0.0	0.0	0.0	0.0	0.0	0.0	0.0	0.0	0.0
0.1	0.1	0.0	0.0	0.0	0.0	0.0	0.1	0.0	0.0	0.0	0.0	0.0	0.0	0.0	0.1	0.0	0.0
0.1	0.0	0.0	0.0	0.0	0.0	0.0	0.0	0.0	0.0	0.0	0.0	0.0	0.0	0.0	0.0	0.0	0.0
0.0	0.0	0.0	0.0	0.0	0.0	0.0	0.0	0.0	0.0	0.0	0.0	0.0	0.0	0.0	0.0	0.0	0.0
0.0	0.0	0.0	0.0	0.0	0.0	0.0	0.1	0.0	0.0	0.0	0.0	0.0	0.0	0.0	0.0	0.0	0.0
0.2	0.0	0.1	0.1	0.1	0.2	0.1	0.1	0.2	0.0	0.0	0.0	1.0	0.2	0.1	0.0	0.0	0.0
0.0	0.0	0.0	0.1	0.0	0.0	0.0	0.1	0.0	0.0	0.0	0.0	0.0	0.0	0.0	0.0	0.0	0.0
0.0	0.0	0.0	0.0	0.0	0.0	0.0	0.0	0.0	0.0	0.0	0.0	0.0	0.0	0.0	0.0	0.0	0.0
1.0	1.0	5.6	19.8	1.5	2.1	0.3	3.2	0.0	2.8	7.4	1.2	0.5	22.8	0.9	6.9	1.1	7.1
0.0	0.0	0.0	0.0	0.0	0.0	0.0	0.0	0.1	0.0	0.0	0.0	0.1	0.0	0.0	0.0	0.0	0.0
0.0	0.1	0.1	0.1	0.1	0.0	0.0	0.1	0.0	0.0	0.0	0.0	0.0	0.1	0.0	0.1	0.0	0.0
0.0	0.0	0.0	0.0	0.0	0.0	0.0	0.0	0.0	0.0	0.0	0.0	0.0	0.0	0.0	0.0	0.0	0.0
0.0	0.0	0.0	0.0	0.0	0.0	0.0	0.0	0.0	0.0	0.0	0.0	0.0	0.0	0.0	0.0	0.0	0.0
0.0	0.0	0.0	0.0	0.0	0.0	0.0	0.0	0.0	0.0	0.0	0.0	0.0	0.0	0.0	0.0	0.0	0.0
0.0	0.1	0.0	0.0	0.1	0.0	0.1	0.3	0.1	0.0	0.0	0.0	0.0	0.1	0.0	0.0	0.0	0.0
0.1	0.2	0.3	0.5	0.1	0.0	0.0	0.6	0.0	0.2	0.0	0.2	0.0	0.1	0.1	1.0	0.1	0.2
0.0	0.0	0.0	0.0	0.0	0.0	0.1	0.0	0.0	0.0	0.0	0.0	0.0	0.0	0.0	0.0	0.0	0.0
0.0	0.0	0.0	0.0	0.1	0.0	0.1	0.0	0.0	0.1	0.0	0.0	0.0	0.0	0.0	0.0	0.0	0.1
0.1	0.0	0.0	0.0	1.7	0.0	0.0	0.0	0.0	0.0	0.0	0.0	0.0	0.0	0.0	3.3	0.3	0.0
0.0	0.0	0.1	0.1	0.0	0.0	0.0	0.0	0.0	0.0	0.0	0.0	0.0	0.0	0.0	0.2	0.2	0.1
0.0	0.2	0.0	0.1	0.0	0.0	0.3	0.0	0.0	0.0	0.0	0.0	0.3	0.0	0.0	0.1	0.1	0.0
0.0	0.0	0.0	10.5	0.0	0.0	0.0	0.0	0.0	0.0	0.0	0.0	0.0	0.0	0.0	0.0	0.0	0.1
0.0	0.1	0.0	0.0	0.1	0.0	0.1	0.0	0.0	0.0	0.0	0.1	0.1	0.2	0.0	0.0	0.2	0.1
0.0	0.0	0.0	0.0	0.0	0.0	0.0	0.0	0.0	0.1	0.0	0.0	0.0	0.0	0.0	0.1	0.0	0.0
13.1	5.6	1.8	0.2	0.9	0.2	4.3	4.5	11.1	0.0	0.0	0.1	2.0	0.0	7.6	1.3	0.1	0.0
0.0	0.0	0.0	0.0	0.1	0.1	0.0	0.0	0.0	0.0	0.0	0.0	0.0	0.0	0.0	0.0	0.0	0.0
0.0	0.0	0.0	0.0	0.0	0.0	0.0	0.0	0.0	0.0	0.0	0.0	0.0	0.0	0.0	0.0	0.0	0.0
0.0	0.0	0.0	0.1	0.0	0.0	0.0	0.0	0.0	0.0	0.0	0.0	0.0	0.0	0.0	0.0	0.0	0.0
0.3	0.1	0.0	0.1	0.0	0.0	0.0	0.0	0.0	0.0	0.0	0.1	0.0	0.1	0.0	0.1	0.0	0.0
0.0	0.0	0.0	0.0	0.0	0.0	0.0	0.1	0.0	0.0	0.0	0.0	0.0	0.0	0.0	0.0	0.0	0.0
0.0	0.0	0.0	0.0	0.0	0.0	0.0	0.0	0.0	0.0	0.0	0.0	0.0	0.0	0.0	0.0	0.0	0.0
0.1	0.1	0.0	0.1	0.1	0.1	0.1	0.0	0.1	0.0	0.0	0.0	0.0	0.0	0.0	0.1	0.1	0.1
0.0	0.0	0.0	0.0	0.0	0.0	0.0	0.0	0.0	0.0	0.0	0.0	0.0	0.0	0.0	0.0	0.0	0.0

0.0	0.0	0.0	0.0	0.0	0.0	0.0	0.0	0.0	0.0	0.0	0.0	0.0	0.0	0.0	0.0	0.0	0.0
0.0	0.0	0.0	0.0	0.0	0.0	0.0	0.0	0.0	0.0	0.0	0.0	0.0	0.0	0.0	0.0	0.0	0.0
7.8	8.1	2.2	8.6	3.9	1.0	9.4	4.9	10.6	0.2	0.0	0.1	14.7	22.4	3.8	1.9	0.3	1.0
0.0	0.0	0.0	0.2	0.1	0.0	0.1	0.0	0.0	0.1	0.0	0.0	0.0	0.0	0.0	0.0	0.0	0.1
0.1	0.0	0.1	0.0	0.0	0.0	0.1	0.2	0.0	0.0	0.0	0.0	0.0	0.0	0.0	0.1	0.0	0.0
0.0	0.0	0.0	0.0	0.6	0.0	0.0	0.0	0.0	0.0	0.0	0.0	0.0	0.0	0.0	0.0	0.0	0.0
0.0	0.0	0.0	0.0	0.0	0.0	0.0	0.0	0.0	1.1	1.5	2.1	0.0	0.0	0.0	0.0	0.2	1.4
0.1	0.1	0.0	0.1	0.1	0.1	0.0	0.0	0.1	0.0	0.0	0.0	0.0	0.0	0.0	0.1	0.0	0.0
0.0	0.0	0.0	0.0	0.0	0.0	0.0	0.1	0.0	0.1	0.0	0.0	0.0	0.0	0.1	0.2	0.0	0.2
0.1	0.0	0.0	0.0	0.5	0.0	0.0	0.0	0.0	0.4	0.0	0.0	28.8	0.1	28.0	0.0	0.0	0.0
0.0	0.0	0.0	0.0	0.0	0.0	0.0	0.0	0.0	0.0	0.0	0.0	0.0	0.0	0.0	0.0	0.0	0.0
0.0	0.0	0.0	0.0	0.0	0.0	0.0	0.0	0.0	0.0	0.0	0.0	0.0	0.0	0.0	0.0	0.0	0.0
0.1	0.4	0.1	0.1	0.1	0.0	0.3	0.0	0.2	0.0	0.0	0.2	0.1	0.2	0.1	0.2	0.0	0.0
0.0	0.0	0.0	0.0	0.0	0.0	0.0	0.0	0.0	0.0	0.0	0.0	0.0	0.0	0.0	0.0	0.0	0.0
14.9	5.3	52.4	0.9	1.0	0.3	2.3	40.6	4.7	0.3	0.0	0.1	0.0	0.1	0.0	9.0	4.3	11.0
0.0	0.0	0.1	0.0	0.0	0.0	0.0	0.0	0.0	0.0	0.0	0.0	0.0	0.0	0.0	0.0	0.0	0.0
0.0	0.0	0.0	0.0	0.0	0.0	0.0	0.0	0.0	0.0	0.0	0.0	0.0	0.0	0.0	0.0	0.0	0.0
3.7	6.0	1.9	1.1	2.1	0.2	4.6	1.4	5.7	0.0	0.0	0.0	4.1	5.2	1.2	0.9	0.1	0.5
0.0	0.0	0.0	0.0	0.0	0.0	0.0	0.0	0.0	0.0	0.0	0.0	0.0	0.0	0.0	0.0	0.0	0.0
0.5	0.9	0.3	0.1	0.1	0.1	0.6	0.3	0.4	0.0	0.0	0.0	0.2	0.5	0.1	0.2	0.0	0.1
0.8	1.3	0.2	4.5	18.2	0.8	1.3	1.7	1.1	30.1	0.5	0.2	0.5	0.2	0.6	4.7	54.2	3.0
11.1	19.1	12.3	2.4	11.5	7.8	20.2	9.7	23.6	0.2	0.0	0.1	6.2	10.3	3.1	1.7	0.1	0.6
0.0	0.0	0.0	0.0	0.0	0.0	0.0	0.0	0.0	0.0	0.0	0.0	0.1	0.0	0.0	0.0	0.0	0.0
0.0	0.0	0.0	0.0	0.0	0.0	0.6	0.0	0.0	0.1	0.0	0.0	0.0	0.0	0.0	0.0	0.0	0.1
0.0	0.0	0.0	0.3	0.0	0.0	0.0	0.0	0.0	0.0	0.0	0.0	0.0	0.0	0.0	0.0	0.0	0.0
0.0	0.0	0.0	0.0	0.0	0.0	0.0	0.0	0.0	0.0	0.0	0.0	0.0	0.0	0.0	0.0	0.0	0.0
0.0	0.0	0.0	0.1	0.0	0.0	0.0	0.0	0.0	0.0	0.0	0.0	0.0	0.0	0.0	0.0	0.0	0.0
0.4	0.5	0.1	0.0	0.3	0.1	0.1	0.0	0.1	0.0	0.0	0.0	0.0	0.0	0.0	0.0	0.0	0.0
0.0	0.0	0.0	0.0	0.0	0.0	0.1	0.0	0.0	0.0	0.0	0.0	0.0	0.0	0.0	0.0	0.0	0.0
0.0	0.0	0.0	0.0	0.0	0.0	0.1	0.0	0.0	0.0	0.0	0.1	0.0	0.0	0.0	0.0	0.0	0.0
0.3	0.0	0.0	0.0	0.0	0.0	0.0	0.2	0.0	0.0	0.0	0.0	0.0	0.1	0.0	0.0	0.0	0.0
0.0	0.0	0.0	0.0	0.0	0.0	0.0	0.0	0.0	0.0	0.0	0.0	0.0	0.0	0.0	0.0	0.0	0.0
0.0	0.0	0.0	0.0	0.0	0.0	0.0	0.0	0.0	0.0	0.0	0.0	0.0	0.0	0.0	0.0	0.0	0.0
0.8	0.2	0.1	0.4	0.3	0.0	0.3	0.1	0.1	0.0	0.0	0.0	0.0	0.0	0.0	0.1	0.0	0.1
0.4	0.5	0.3	0.3	1.5	0.1	0.6	0.5	0.5	0.0	0.0	0.0	0.3	0.6	0.2	0.5	0.0	0.3

AF 2-5 cm 1	AF 2-5 cm 2	AF 2-5 cm 3	AF 5-10 cm 1	AF 5-10 cm 2	AF 5-10 cm 3	\ forest floor	\ forest floor	\ forest floor	A 2-5 cm 1	A 2-5 cm 2	A 2-5 cm 3
0.1	0.7	0.1	0.6	0.0	0.0	0.0	0.1	0.0	0.0	0.1	0.1
0.2	0.0	0.3	0.0	0.0	0.1	0.0	0.0	0.0	0.0	0.0	0.0
0.0	0.0	0.1	0.0	0.1	3.1	0.8	0.1	0.0	0.6	0.3	0.9
0.0	0.1	0.0	0.0	0.2	0.0	0.0	0.0	0.0	0.1	0.5	0.0
0.0	0.3	0.0	0.1	0.0	0.0	0.0	0.0	0.0	0.0	0.1	0.0
0.0	0.0	0.4	0.0	0.0	0.0	0.0	0.0	0.0	0.0	0.0	0.1
0.0	0.0	0.0	0.0	0.0	0.0	0.0	0.2	0.0	0.0	0.0	0.0
0.8	3.4	0.0	0.2	0.2	0.1	76.7	29.1	47.2	1.6	11.9	8.0
0.7	1.3	0.3	0.9	0.3	0.6	0.0	0.0	0.0	0.1	0.2	0.1
0.1	0.0	0.0	0.0	0.0	0.1	0.0	0.0	0.1	0.0	0.0	0.0
0.0	0.0	0.0	0.0	0.0	0.0	0.0	0.0	0.0	0.0	0.0	0.0
0.5	0.0	0.0	0.0	0.0	0.0	0.0	0.0	0.0	0.0	0.0	0.0
0.0	0.0	0.0	0.0	0.0	0.0	0.0	0.0	0.0	0.0	0.0	0.0
0.0	0.0	0.0	0.0	0.0	0.0	0.1	0.0	0.0	0.0	0.0	0.0
0.1	0.3	0.9	0.3	0.8	1.0	0.0	0.0	0.0	0.5	0.9	0.7
0.0	0.1	0.0	0.0	0.1	0.0	0.0	0.0	0.0	0.0	0.0	0.0
0.0	0.0	0.0	0.0	0.0	0.0	0.0	0.0	0.0	0.0	0.0	0.0
0.0	0.0	0.0	0.0	0.0	0.0	0.0	0.0	0.0	0.0	0.0	0.0
0.0	0.1	0.1	0.1	0.0	0.0	0.0	0.0	0.0	0.0	0.0	0.1
0.0	0.0	0.0	0.1	0.0	0.0	0.0	0.0	0.0	0.0	0.0	0.0
0.4	0.1	0.3	0.8	0.1	0.2	0.4	1.7	1.2	0.4	0.6	0.2
0.0	0.0	0.0	0.0	0.0	0.0	0.0	0.0	0.0	0.0	0.0	0.0
0.0	6.0	0.0	0.2	0.1	0.4	0.0	0.0	0.1	0.2	0.1	0.0
0.1	0.0	0.2	0.0	0.3	0.6	2.5	7.7	6.2	1.8	1.6	0.1
0.0	0.0	0.0	0.0	0.0	0.7	0.0	0.2	0.0	2.5	0.0	0.0
0.0	0.0	0.0	0.0	0.0	0.0	0.0	0.0	0.0	0.0	0.0	0.0
0.1	0.0	0.0	0.1	0.0	0.0	0.0	0.0	0.0	0.0	0.0	0.0
0.0	0.0	0.0	0.0	0.0	0.0	0.0	0.0	0.0	0.0	0.0	0.0
0.2	0.2	0.2	0.3	0.2	0.3	0.0	0.0	0.0	0.1	0.3	0.2
0.0	0.0	0.0	0.0	0.0	2.3	0.0	0.0	0.0	1.8	0.0	0.8
0.0	0.0	0.0	0.0	0.0	0.0	0.0	0.0	0.0	0.0	0.0	0.0
0.5	0.4	0.2	0.5	0.9	0.3	0.0	0.0	0.0	0.2	0.0	0.1
0.8	2.2	0.4	1.0	1.6	0.2	0.1	0.1	0.1	0.3	0.4	0.5
0.1	0.1	0.0	0.0	0.0	0.1	0.0	0.0	0.0	0.0	0.0	0.0
0.0	0.1	0.1	0.1	0.0	0.0	0.0	0.0	0.1	0.0	0.1	0.0
0.0	0.0	0.0	0.0	0.0	0.0	0.0	0.4	0.4	0.0	0.0	0.0
0.1	0.0	0.0	0.0	0.1	0.0	0.0	0.0	0.0	0.0	0.0	0.0
0.0	0.1	0.1	0.0	0.2	0.0	0.0	0.0	0.0	0.0	0.1	0.3
0.0	0.0	0.0	0.0	0.0	0.0	0.0	0.0	0.0	0.0	0.0	0.0
0.0	0.0	0.0	0.0	0.0	0.0	0.0	0.0	0.0	0.0	0.0	0.1
0.0	0.0	0.0	0.0	0.0	0.1	0.0	0.0	0.0	0.0	0.0	0.0
0.0	0.0	0.0	0.0	0.0	0.0	0.0	0.0	0.0	0.0	0.0	0.0
0.0	0.0	0.0	0.0	0.0	0.0	0.0	0.0	0.0	0.0	0.0	0.0
0.0	0.0	0.0	0.0	0.0	0.1	0.0	0.0	0.0	0.0	0.0	0.0
0.0	0.0	0.0	0.0	0.0	0.1	0.0	0.0	0.0	0.0	0.0	0.0
0.1	0.0	0.1	0.0	0.0	0.1	0.0	0.0	0.1	0.0	0.0	0.0
1.0	0.2	0.4	1.3	0.4	0.6	0.1	0.1	0.0	0.2	0.4	0.2
0.0	0.0	0.0	0.0	0.0	0.0	0.0	0.0	0.0	0.0	0.0	0.0
0.1	0.0	0.5	0.0	0.0	0.7	0.1	0.0	0.0	0.4	0.0	0.0
0.0	0.0	0.0	0.0	0.0	0.0	0.0	0.0	0.0	0.0	0.0	0.0
0.0	0.5	0.0	0.0	0.4	0.0	0.0	0.0	0.0	0.1	0.0	0.0
0.0	0.0	0.0	0.0	0.0	0.0	0.0	0.0	0.0	0.0	0.0	0.0
0.0	0.1	0.0	0.0	0.5	0.0	0.0	0.0	0.0	0.1	0.1	0.0
0.2	0.0	0.5	0.2	0.0	0.1	0.1	0.2	0.2	0.3	0.1	0.1
0.0	0.0	0.0	0.0	0.0	0.0	0.0	0.4	0.2	0.0	0.0	0.0
0.0	0.0	0.0	0.0	0.0	0.0	0.0	0.0	0.0	0.0	0.0	0.0
0.0	0.0	0.0	0.0	0.0	0.0	0.0	0.0	0.0	0.0	0.0	0.0
0.1	0.0	0.4	0.1	0.1	0.1	0.0	0.0	0.0	0.1	0.4	0.5
2.7	0.8	1.7	2.2	1.0	1.0	0.3	0.1	0.2	1.0	0.4	0.6
0.1	0.0	0.2	0.2	0.0	0.7	0.0	0.0	0.2	0.5	0.2	0.3
0.0	0.1	0.0	0.0	0.2	0.0	0.0	0.0	0.0	0.0	0.0	0.0
0.0	0.0	0.0	0.0	0.0	0.0	0.0	0.0	0.0	0.0	0.0	0.0
0.0	0.1	0.3	0.0	0.1	12.0	0.0	0.0	0.0	3.5	4.3	3.7
0.2	0.2	0.4	0.1	0.2	0.0	0.1	0.1	0.1	0.1	0.3	0.2
1.0	0.0	0.1	0.1	0.0	0.0	0.1	0.6	0.3	0.0	0.0	0.0

0.0	0.0	0.0	0.0	0.0	0.0	0.0	0.0	0.0	0.0	0.0	0.0
0.0	0.0	0.0	0.0	0.0	0.0	0.0	0.0	0.0	0.0	0.0	0.0
3.3	3.0	3.1	3.4	2.3	0.0	0.0	0.0	0.0	0.0	0.1	0.0
1.0	0.3	0.8	1.0	1.0	0.3	0.3	0.0	0.3	0.3	0.2	0.3
0.0	0.1	0.0	0.0	0.0	0.3	0.0	0.0	0.0	0.0	0.2	0.0
0.0	0.0	0.1	0.0	0.0	0.0	0.0	0.0	0.0	0.1	0.1	0.1
0.0	0.0	0.0	0.0	0.0	0.2	0.0	0.0	0.0	0.0	0.0	0.0
0.0	0.0	0.0	0.0	0.0	0.0	0.0	0.0	0.0	0.0	0.0	0.0
0.2	0.0	0.1	0.0	0.2	0.0	0.0	0.0	0.1	0.0	0.0	0.0
0.1	0.0	0.1	0.4	0.5	5.3	0.1	0.6	0.0	1.6	0.5	1.1
0.0	0.0	0.1	0.1	0.0	0.1	0.0	0.0	0.0	0.4	0.3	0.3
0.1	0.0	3.2	1.6	0.0	3.7	0.6	1.0	1.7	12.0	11.9	33.3
0.0	0.0	0.0	0.0	0.0	0.0	0.0	0.0	0.0	0.0	0.1	0.0
0.0	0.0	0.3	0.0	0.0	0.0	0.0	0.0	0.0	0.0	0.0	0.0
0.0	0.0	0.0	0.0	0.0	0.0	0.0	0.0	0.0	0.0	0.0	0.0
0.0	0.0	0.0	0.1	0.0	0.0	0.0	0.0	0.0	0.0	0.0	0.0
0.0	0.0	0.0	0.1	0.0	0.1	0.0	0.0	0.0	0.0	0.2	0.1
0.0	0.0	0.0	0.0	0.0	0.0	0.0	0.0	0.0	0.0	0.0	0.0
0.0	0.0	0.0	0.0	0.0	0.0	0.0	0.0	0.0	0.0	0.0	0.0
0.0	0.0	0.0	0.0	0.0	0.0	0.0	0.0	0.0	0.0	0.0	0.0
0.0	0.0	0.0	0.0	0.0	0.0	0.0	0.0	0.0	0.0	0.0	0.0
0.1	0.0	0.0	0.0	0.0	0.0	0.0	0.0	0.1	0.0	0.0	0.0
0.0	0.0	0.0	0.0	0.0	0.0	0.0	0.0	0.0	0.0	0.0	0.0
0.0	0.0	0.0	0.0	0.0	0.0	0.0	0.0	0.0	0.0	0.0	0.0
0.0	0.0	0.0	0.0	0.0	0.0	0.0	0.0	0.0	0.0	0.0	0.0
0.5	0.1	0.3	0.4	0.1	0.2	0.1	0.2	0.0	0.1	0.2	0.2
2.4	0.6	0.8	3.9	0.8	0.9	0.2	0.7	0.3	0.3	0.6	0.4
0.0	0.0	0.0	0.0	0.0	0.0	0.0	0.0	0.0	0.0	0.0	0.0
0.0	0.0	0.0	0.0	0.0	0.0	0.0	0.0	0.0	0.0	0.0	0.0
0.0	0.0	0.0	0.0	0.0	0.0	0.0	0.0	0.0	0.0	0.1	0.0
0.0	0.0	0.0	0.0	0.0	0.0	0.0	0.0	0.0	0.0	0.0	0.0
0.0	0.0	0.0	0.0	0.0	0.0	0.0	0.0	0.0	0.0	0.0	0.0
1.5	0.2	0.1	0.2	0.3	0.1	0.3	0.3	0.6	0.3	0.2	0.2
0.0	0.0	0.0	0.0	0.0	0.0	0.0	0.0	0.0	0.0	0.0	0.0
0.0	0.0	0.0	0.0	0.0	0.0	0.0	0.0	0.0	0.0	0.0	0.0
0.0	0.0	0.0	0.2	0.0	0.2	0.0	0.0	0.0	0.1	0.1	0.1
0.0	0.0	0.0	0.0	0.0	0.0	0.0	0.0	0.0	0.0	0.0	0.0
0.3	0.0	0.0	0.4	0.0	0.0	0.0	0.0	0.0	0.0	0.0	0.0
0.0	0.0	0.1	0.0	0.1	0.3	0.0	0.0	3.9	0.1	0.3	0.1
0.0	0.0	0.0	0.0	0.0	0.1	0.0	0.0	0.0	0.0	0.0	0.0
0.0	0.0	0.0	0.0	0.0	0.0	0.0	0.0	0.0	0.0	0.0	0.6
2.2	0.4	0.9	1.6	0.6	1.4	0.3	0.3	0.6	0.7	0.5	0.9
0.0	0.0	0.0	0.0	0.0	0.0	0.0	0.0	0.0	0.0	0.0	0.0
0.3	0.1	0.1	0.4	0.4	0.2	0.0	0.1	0.0	0.0	0.4	0.1
2.3	0.7	1.4	4.9	0.8	3.8	0.0	0.0	0.0	0.7	2.4	1.6
0.0	0.0	0.1	0.0	0.0	0.0	0.0	0.2	0.1	0.0	0.8	0.5
0.0	0.0	0.0	0.0	0.0	0.0	0.0	0.0	0.0	0.0	0.0	0.0
0.0	0.0	0.0	0.0	0.0	0.0	0.0	0.0	0.0	0.0	0.0	0.0
0.0	0.0	0.0	0.0	0.0	0.0	0.0	0.0	0.0	0.0	0.0	0.2
0.4	0.0	0.0	0.4	0.0	0.0	0.1	0.0	0.0	0.6	0.2	0.0
0.0	0.1	1.0	0.0	0.1	0.0	0.1	0.0	0.1	0.1	0.0	0.0
0.0	0.0	0.0	0.0	0.0	0.0	0.0	0.0	0.0	0.0	0.0	0.0
0.9	0.0	0.7	0.7	0.0	0.2	0.0	0.0	0.0	0.4	0.2	0.3
0.0	0.0	0.0	0.0	0.0	0.0	0.0	0.0	0.0	0.0	0.0	0.0
0.8	0.0	0.0	0.1	0.0	0.0	0.2	0.0	0.0	0.1	0.0	0.0
0.0	0.0	0.0	0.0	0.0	0.0	0.6	0.0	0.0	0.3	0.4	0.1
0.0	0.0	0.0	0.0	0.0	0.0	0.0	0.0	0.0	0.0	0.0	0.0
0.1	0.0	0.0	0.0	0.0	0.1	0.0	0.0	0.0	0.0	0.1	0.0
0.2	0.1	0.1	0.1	0.0	0.1	0.0	0.0	0.0	0.1	0.1	0.0
0.0	0.0	0.0	0.0	0.0	0.0	0.0	0.0	0.0	0.0	0.0	0.0
0.1	0.1	0.0	0.3	0.1	0.1	0.0	0.1	0.0	0.0	0.0	0.0
0.1	0.0	0.0	0.0	0.0	0.0	0.1	0.1	0.1	0.0	0.0	0.0
0.0	0.0	0.0	0.0	0.0	0.0	0.1	0.1	0.1	0.1	0.0	0.0
0.0	0.0	0.0	0.0	0.0	0.0	0.0	0.0	0.0	0.0	0.0	0.0
0.5	0.8	0.2	0.7	1.5	0.2	0.0	0.0	0.1	0.1	0.5	0.0
0.0	0.0	0.1	0.2	0.1	0.9	0.0	0.2	0.1	0.2	0.4	0.1
2.2	0.8	0.6	0.7	0.9	0.7	1.4	2.8	6.9	0.5	0.2	0.4
0.0	0.0	0.0	0.0	0.0	0.0	0.0	0.0	0.0	0.0	0.0	0.0
0.0	0.1	0.2	0.0	0.0	0.0	0.0	0.0	0.0	0.0	0.0	0.0
0.3	0.0	0.0	0.0	0.0	0.0	0.0	0.7	0.9	0.0	0.0	0.0
1.9	0.3	0.8	1.1	0.8	1.6	0.8	0.8	1.1	1.3	1.6	2.7

0.0	0.0	0.0	0.0	0.0	0.0	0.0	0.0	0.0	0.0	0.0	0.0
0.7	0.0	2.0	0.5	0.0	0.3	0.2	2.0	2.6	0.1	0.0	0.0
0.1	0.4	0.2	0.0	0.1	0.1	0.0	0.1	0.1	0.1	0.0	0.0
0.0	0.0	0.0	0.0	0.0	2.5	0.0	0.0	0.0	0.3	0.5	0.1
0.0	0.0	0.0	0.0	0.0	0.0	0.0	0.0	0.0	0.0	0.0	0.0
0.0	0.0	0.0	0.0	0.0	0.0	0.0	0.0	0.0	0.0	0.0	0.0
0.0	0.0	0.0	0.0	0.0	0.0	0.0	0.1	3.1	0.0	0.0	0.0
0.0	0.0	0.0	0.0	0.0	0.0	0.0	0.2	0.0	0.0	0.0	0.0
0.0	0.0	0.0	0.0	0.3	0.0	0.0	0.0	0.1	0.0	0.0	0.0
0.6	0.1	0.3	0.3	0.1	0.2	0.5	0.4	0.5	0.2	0.3	0.2
0.0	0.0	0.0	0.0	0.0	0.0	0.0	0.0	0.0	0.0	0.0	0.0
0.0	0.0	0.0	0.0	0.1	0.0	0.0	0.0	0.0	0.0	0.0	0.1
0.1	0.0	0.0	0.1	0.0	0.0	0.0	0.0	0.0	0.1	0.0	0.5
0.0	0.0	0.1	0.0	0.0	0.1	0.0	0.0	0.0	0.0	0.0	0.1
0.0	0.0	0.0	0.0	0.0	0.1	0.0	0.0	0.0	0.0	0.0	0.0
0.0	0.1	0.1	0.1	0.1	0.1	0.0	0.0	0.0	0.0	0.1	0.2
0.0	0.0	0.1	0.1	0.0	0.0	0.0	0.0	0.0	0.0	0.0	0.1
0.0	0.0	0.1	0.0	0.0	0.0	0.0	0.0	0.0	0.0	0.0	0.0
0.0	0.0	0.0	0.0	0.1	0.0	0.0	0.0	0.0	0.0	0.0	0.0
0.0	0.0	0.0	0.0	0.0	0.0	0.0	0.0	0.0	0.0	0.0	0.0
0.0	0.0	0.0	0.0	0.0	0.0	0.0	0.0	0.0	0.0	0.0	0.0
0.0	0.0	0.0	0.0	0.0	0.0	0.0	0.0	0.0	0.0	0.0	0.0
0.5	0.0	0.1	0.1	0.1	0.0	0.0	0.1	0.1	0.0	0.0	0.0
0.0	0.0	0.0	0.0	0.0	0.0	0.0	0.0	0.0	0.0	0.0	0.0
0.0	0.0	0.0	0.0	0.0	0.0	0.0	0.0	0.0	0.0	0.0	0.0
0.0	0.0	0.0	0.0	0.0	0.0	0.0	0.0	0.0	0.0	0.0	0.0
0.0	0.0	0.0	0.0	0.0	0.1	0.0	0.0	0.0	0.0	0.0	0.0
0.1	1.0	0.6	0.2	1.3	0.0	0.0	0.0	0.0	0.0	0.1	0.1
0.0	0.0	0.0	0.0	0.0	0.0	0.0	0.0	0.0	0.0	0.0	0.0
0.0	0.0	0.0	0.0	0.0	0.0	0.0	0.0	0.0	0.0	0.0	0.0
0.0	0.0	0.0	0.0	0.0	0.0	0.0	21.2	0.0	0.0	0.0	0.0
0.1	0.0	0.0	0.1	0.0	0.1	0.0	0.1	0.3	0.0	0.0	0.0
0.0	0.0	0.0	0.0	0.0	0.0	0.0	0.0	0.0	0.0	0.0	0.0
0.0	0.0	0.0	0.0	0.0	0.0	0.0	0.0	0.0	0.0	0.0	0.0
0.0	0.0	0.0	0.0	0.0	0.0	0.0	0.0	0.3	0.0	0.0	0.0
0.0	0.0	0.0	0.1	0.0	0.0	0.0	0.0	0.0	0.0	0.0	0.0
0.1	0.3	0.1	0.0	0.0	0.0	0.0	0.0	0.0	0.0	0.1	0.0
0.0	0.0	0.0	0.0	0.0	0.0	0.0	0.0	0.0	0.0	0.0	0.0
0.0	0.0	0.0	0.0	0.0	0.0	0.0	0.0	0.0	0.0	0.0	0.0
2.0	0.0	0.0	0.2	0.0	0.0	0.0	0.0	0.0	0.0	0.0	0.0
0.0	0.0	0.0	0.0	0.0	0.0	0.0	0.0	0.0	0.0	0.0	0.0
0.0	0.0	0.0	0.0	0.0	0.0	0.0	0.0	0.0	0.0	0.0	0.0
0.1	0.4	0.3	0.1	0.4	0.9	0.0	0.0	0.0	0.4	1.4	1.6
0.0	0.0	0.0	0.0	0.0	0.0	0.0	0.0	0.0	0.0	0.0	0.0
0.0	0.0	0.0	0.1	0.0	0.0	0.0	0.2	0.3	0.0	0.0	0.0
1.1	0.0	0.4	2.9	1.7	1.1	0.0	0.0	0.0	0.4	1.3	0.6
0.0	0.0	0.0	0.0	0.0	0.0	0.0	0.0	0.0	0.0	0.0	0.0
0.0	0.0	0.0	0.0	0.0	0.0	0.0	0.0	0.0	0.0	0.0	0.0
0.0	0.0	0.0	0.0	0.0	0.0	0.0	0.0	0.0	0.0	0.0	0.0
0.0	0.0	0.0	0.0	0.0	0.0	0.0	0.0	0.0	0.0	0.0	0.0
0.0	0.0	0.0	0.0	0.0	0.0	0.0	0.0	0.0	0.0	0.0	0.0
0.1	0.0	0.1	0.0	0.0	0.0	0.0	0.1	0.1	0.0	0.0	0.1
0.0	0.0	0.0	0.0	0.0	0.0	0.0	0.0	0.0	0.0	0.0	0.0
0.9	0.6	0.2	0.2	0.5	0.1	0.0	0.0	0.0	0.0	0.1	0.0
5.0	3.2	1.2	3.5	3.1	0.6	0.1	0.2	0.1	0.2	0.6	0.5
0.0	0.0	0.0	0.0	0.0	0.0	0.0	0.0	0.0	0.0	0.0	0.0
1.5	0.2	0.8	1.5	1.1	0.2	0.0	0.0	0.0	0.3	1.1	0.3
0.1	0.0	0.0	0.1	0.0	0.0	0.0	0.1	0.0	0.0	0.1	0.1
0.0	0.0	0.0	0.0	0.0	0.0	0.0	0.0	0.0	0.0	0.0	0.0
0.2	0.6	0.3	0.2	0.2	0.8	0.0	0.1	0.2	0.1	0.1	0.1
0.2	0.1	0.0	0.0	0.2	0.1	0.0	0.0	0.0	0.0	0.0	0.0
0.1	0.1	0.1	0.0	0.1	0.1	0.0	0.1	0.0	0.1	0.1	0.1
0.0	0.0	0.0	0.0	0.0	0.0	0.0	0.0	0.0	0.0	0.0	0.0
0.0	0.0	0.0	0.0	0.0	0.1	0.0	0.0	0.0	0.0	0.0	0.0
0.0	0.1	0.0	0.2	0.1	0.0	0.0	0.0	0.0	0.0	0.0	0.0
0.0	0.0	0.0	0.2	0.0	0.1	0.0	0.0	0.0	0.0	0.1	0.0
0.0	0.0	0.0	0.0	0.0	0.0	0.0	0.0	0.0	0.0	0.0	0.0
0.2	0.0	0.0	0.1	0.0	0.0	0.0	0.0	0.0	0.0	0.0	0.0
0.1	0.0	0.0	0.1	0.1	0.0	0.0	0.0	0.0	0.2	0.1	0.0
0.1	0.2	0.0	0.0	0.0	0.7	0.0	0.0	0.0	0.1	0.0	0.0
0.0	0.1	0.0	0.0	0.0	0.0	0.2	0.6	1.8	0.0	0.0	0.1

0.0	0.0	0.0	0.0	0.0	0.0	0.0	0.0	0.0	0.0	0.0	0.0
0.0	0.0	0.0	0.0	0.0	0.0	0.0	0.0	0.0	0.0	0.0	0.0
0.3	0.6	0.1	0.6	0.1	22.4	0.0	0.0	0.0	4.0	13.5	1.7
0.1	0.1	0.2	0.0	0.1	0.0	0.0	0.0	0.1	0.0	0.1	0.1
0.0	0.0	0.0	0.0	0.0	0.0	0.0	0.0	0.0	0.0	0.0	0.0
0.2	0.0	0.2	0.2	0.3	0.0	0.0	0.0	0.0	0.1	0.0	0.0
0.0	0.0	0.0	0.0	0.0	0.0	0.0	0.0	0.0	0.0	0.0	0.0
0.0	0.0	0.0	0.0	0.0	0.0	0.0	0.0	0.0	0.0	0.0	0.0
0.0	0.0	0.0	0.0	0.0	0.1	0.0	0.0	0.2	0.0	0.0	0.0
0.0	0.0	0.0	0.0	0.1	0.0	0.0	0.0	0.0	0.0	0.0	0.0
0.0	0.0	0.0	0.0	0.0	0.0	0.0	0.0	0.0	0.0	0.0	0.0
0.5	0.0	0.0	0.7	0.0	0.0	0.0	0.0	0.0	0.0	0.0	0.0
0.0	0.0	0.0	0.0	0.0	0.0	0.0	0.0	0.0	0.0	0.1	0.0
0.0	0.0	0.0	0.0	0.0	0.0	0.0	0.0	0.0	0.0	0.0	0.0
0.0	0.0	0.0	0.0	0.0	0.0	0.0	0.0	0.0	0.0	0.0	0.0
0.0	0.0	0.1	0.0	0.1	0.1	0.0	0.1	0.0	0.0	0.0	0.1
0.4	0.4	0.0	0.3	0.1	0.1	0.1	0.2	0.1	0.1	0.0	0.1
0.0	0.1	0.0	0.0	0.1	0.0	0.0	0.0	0.0	0.0	0.0	0.1
2.0	0.5	1.7	4.0	1.6	1.2	0.0	0.1	0.0	0.0	1.6	0.7
0.0	0.0	0.0	0.0	0.0	0.0	0.0	0.0	0.0	0.0	0.0	0.0
0.0	0.0	0.0	0.0	0.0	0.1	0.0	0.0	0.0	0.0	0.0	0.0
0.0	0.0	0.0	0.0	0.0	0.0	0.0	0.0	0.0	0.0	0.0	0.0
0.0	0.0	0.0	0.1	0.0	0.0	0.0	0.0	0.0	0.0	0.0	0.0
0.1	0.1	0.0	0.2	0.3	0.1	0.0	0.0	0.0	0.0	0.0	0.0
0.0	0.0	0.0	0.0	0.0	0.0	0.0	0.0	0.0	0.0	0.0	0.0
0.0	0.0	0.0	0.0	0.0	0.0	3.0	0.0	0.0	0.0	0.0	0.0
0.0	0.0	0.0	0.2	0.0	0.0	0.0	0.0	0.0	0.0	0.0	0.0
0.0	0.0	0.0	0.0	0.0	0.0	0.1	0.0	0.0	0.0	0.0	0.0
0.0	0.1	0.4	0.0	0.3	0.6	0.0	0.0	0.0	0.6	0.5	0.2
0.2	0.7	0.2	0.5	0.8	0.2	0.0	1.6	2.1	0.1	0.0	0.2
0.1	2.5	0.0	0.0	2.5	0.0	0.1	0.1	0.4	0.0	0.0	0.0
0.0	0.3	0.2	0.2	0.3	0.1	0.0	0.0	0.0	0.1	0.2	0.2
0.0	0.0	0.0	0.0	0.0	0.0	0.0	0.0	0.0	0.1	0.0	0.0
0.0	0.0	0.0	0.0	0.0	0.0	0.0	0.0	0.0	0.0	0.0	0.0
0.0	0.0	0.0	0.0	0.0	0.0	0.0	0.0	0.0	0.0	0.0	0.0
0.1	0.0	0.1	0.0	0.0	0.4	0.0	0.0	0.2	0.2	0.0	0.0
0.1	0.1	0.5	0.0	0.2	0.1	0.0	0.0	0.1	0.4	0.2	1.4
0.0	0.0	0.0	0.0	0.0	0.0	0.0	0.0	0.0	0.0	0.0	0.0
0.0	0.0	0.0	0.0	0.0	0.0	0.0	0.0	0.0	0.0	0.0	0.0
0.0	0.2	0.0	0.0	0.1	0.0	0.0	0.0	0.0	0.0	0.0	0.0
0.0	0.0	0.0	0.0	0.0	0.0	0.0	0.0	0.0	0.0	0.0	0.0
0.0	0.0	0.0	0.0	0.0	0.0	0.0	0.0	0.0	0.0	0.0	0.0
0.0	0.0	0.1	0.0	0.0	0.0	0.0	0.0	0.0	0.0	0.0	0.0
0.0	0.0	0.1	0.0	0.1	0.8	0.0	0.0	0.0	0.2	0.2	2.2
0.0	0.0	0.0	0.0	0.0	0.0	0.0	0.0	0.0	0.0	0.0	0.0
0.0	0.0	0.0	0.0	0.0	0.0	0.0	0.0	0.0	0.0	0.0	0.0
5.6	4.4	23.0	0.7	3.3	0.6	0.7	3.4	6.1	1.0	0.5	1.2
0.1	0.0	0.1	0.0	0.0	0.1	0.0	0.0	0.5	0.0	0.0	0.0
0.1	0.1	0.0	0.0	0.0	0.1	0.0	0.0	0.0	0.0	0.0	0.0
0.0	0.0	0.0	0.0	0.0	0.0	0.0	0.0	0.0	0.0	0.0	0.0
0.0	0.0	0.0	0.0	0.0	0.0	0.0	0.0	0.0	0.0	0.0	0.0
0.0	0.0	0.0	0.0	0.0	0.0	0.0	0.0	0.0	0.0	0.0	0.0
0.0	0.0	0.0	0.0	0.0	0.0	0.0	0.0	0.0	0.0	0.1	0.1
0.8	0.5	0.2	0.4	0.1	0.0	0.1	0.0	0.1	0.1	0.1	3.4
0.0	0.0	0.0	0.0	0.0	0.0	0.0	0.0	0.0	0.0	0.0	0.0
0.0	0.0	0.0	0.0	0.0	0.0	0.1	0.2	0.7	0.0	0.1	0.0
0.2	0.1	0.0	0.0	0.0	0.0	0.0	0.0	0.0	0.0	0.1	0.1
0.0	0.1	0.0	0.0	0.0	0.3	0.0	11.3	0.0	0.3	0.0	0.0
0.1	0.1	0.1	0.1	0.0	0.1	0.0	0.0	0.0	0.0	0.1	0.0
18.8	3.2	25.5	10.9	4.8	0.1	0.0	0.0	0.0	0.0	0.0	0.0
0.0	0.0	0.0	0.1	0.0	0.0	0.1	0.1	0.2	0.0	0.0	0.1
0.0	0.0	0.0	0.0	0.0	0.0	0.0	0.0	0.0	0.0	0.0	0.0
4.5	0.5	0.1	2.5	0.3	0.1	0.0	0.1	0.1	0.1	2.5	1.5
0.0	0.0	0.0	0.0	0.0	0.3	0.0	0.0	0.0	0.4	0.2	0.0
0.0	0.0	0.0	0.0	0.0	0.0	0.0	0.0	0.0	0.0	0.0	0.0
0.0	0.0	0.0	0.0	0.0	0.0	0.0	0.0	0.0	0.0	0.0	0.0
0.0	0.2	0.1	0.6	0.0	0.1	0.0	0.0	0.0	0.2	0.1	0.0
0.1	0.1	0.0	0.1	0.0	0.2	0.0	0.0	0.0	0.0	0.0	0.0
0.0	0.0	0.0	0.0	0.0	0.0	0.0	0.0	0.0	0.0	0.0	0.0
0.0	0.0	0.0	0.0	0.0	0.3	0.0	0.1	0.1	0.0	0.2	0.1
0.0	0.0	0.0	0.0	0.0	0.0	0.2	0.0	0.0	0.0	0.0	0.0

0.0	0.0	0.0	0.0	0.0	0.0	0.0	0.0	0.0	0.0	0.0	0.0
0.0	0.0	0.0	0.0	0.0	0.0	0.0	0.0	0.0	0.0	0.0	0.0
5.9	3.6	7.8	12.7	7.6	1.5	0.0	0.3	0.2	0.8	1.4	2.7
0.0	0.0	0.3	0.0	0.0	0.1	0.0	0.0	0.0	0.1	0.2	0.1
0.1	0.0	0.0	0.1	0.1	0.0	0.0	0.0	0.0	0.0	0.0	0.0
0.0	0.0	0.0	0.0	0.0	0.0	0.0	0.0	0.0	0.0	0.3	0.0
0.0	0.0	0.0	0.0	0.0	0.0	0.0	0.0	0.0	0.0	0.0	0.0
0.0	0.0	0.0	0.0	0.1	0.3	0.0	0.0	0.0	0.2	0.1	0.2
0.0	0.0	0.0	0.0	0.0	0.0	1.3	1.0	0.1	0.4	0.1	0.6
0.0	0.0	0.0	0.0	0.0	0.0	0.0	0.0	0.0	0.0	0.0	0.0
0.0	0.0	0.0	0.0	0.0	0.0	0.0	0.0	0.0	0.0	0.0	0.0
0.0	0.0	0.0	0.0	0.0	0.0	0.0	0.0	0.0	0.0	0.0	0.0
0.5	0.0	0.1	0.6	0.1	0.1	0.0	0.0	0.0	0.1	0.1	0.1
0.0	0.0	0.0	0.0	0.0	0.0	0.0	0.0	0.0	0.0	0.0	0.0
2.1	42.0	0.3	1.6	43.5	1.5	2.6	2.9	2.5	0.9	3.5	1.5
0.0	0.0	0.0	0.0	0.0	0.0	0.0	0.0	0.0	0.0	0.0	0.0
0.0	0.0	0.0	0.0	0.0	0.0	0.0	0.0	0.0	0.0	0.0	0.0
1.0	0.5	0.7	1.3	0.4	0.4	0.0	0.1	0.1	0.1	0.8	0.6
0.0	0.0	0.0	0.0	0.0	0.0	0.0	0.0	0.0	0.0	0.0	0.0
0.1	0.1	0.1	0.2	0.1	0.2	0.0	0.1	0.0	0.1	0.3	0.1
2.4	1.8	3.0	3.1	0.7	0.8	2.3	1.8	1.4	19.4	9.8	2.2
5.5	3.1	1.8	9.7	2.5	9.5	0.1	0.1	0.2	2.2	9.0	6.9
0.0	0.0	0.0	0.2	0.0	0.0	0.0	0.0	0.0	0.0	0.0	0.0
0.0	0.0	0.0	0.0	0.0	0.0	0.0	0.0	0.0	0.0	0.0	0.0
0.0	0.0	0.0	0.0	0.0	0.0	0.0	0.0	0.0	26.8	0.0	3.3
0.0	0.0	0.0	0.0	0.0	0.0	0.0	0.0	0.0	0.0	0.0	0.0
0.0	0.0	0.0	0.0	0.0	0.0	0.0	0.0	0.0	0.0	0.0	0.0
0.1	0.0	0.0	0.1	0.0	0.0	0.0	0.0	0.0	0.0	0.0	0.2
0.0	0.0	0.0	0.0	0.0	0.0	0.0	0.0	0.0	0.0	0.0	0.0
0.0	0.1	0.0	0.0	0.1	0.0	0.0	0.0	0.0	0.0	0.0	0.0
0.0	0.0	0.0	0.0	0.0	0.0	0.0	0.0	0.1	0.0	0.0	0.0
0.0	0.0	0.0	0.0	0.0	0.0	0.0	0.0	0.0	0.0	0.0	0.0
0.0	0.0	0.0	0.0	0.0	0.0	0.0	0.0	0.0	0.0	0.0	0.0
0.1	0.2	0.2	0.4	0.1	0.3	0.0	0.0	0.0	0.0	0.1	0.1
0.4	0.2	1.1	2.3	0.1	0.6	0.0	0.0	0.0	0.2	0.2	0.8
